# Network-based prioritization and validation of regulators of vascular smooth muscle cell proliferation in disease

**DOI:** 10.1038/s44161-024-00474-4

**Published:** 2024-06-06

**Authors:** Jordi Lambert, Sebnem Oc, Matthew D. Worssam, Daniel Häußler, Charles U. Solomon, Nichola L. Figg, Ruby Baxter, Maria Imaz, James C. K. Taylor, Kirsty Foote, Alison Finigan, Krishnaa T. Mahbubani, Tom R. Webb, Shu Ye, Martin R. Bennett, Achim Krüger, Mikhail Spivakov, Helle F. Jørgensen

**Affiliations:** 1 Section of Cardiorespiratory Medicine, Victor Phillip Dahdaleh Heart and Lung Research Institute, University of Cambridge, Cambridge, UK; 2 Functional Gene Control Group, MRC Laboratory of Medical Sciences, London, UK; 3 Institute of Clinical Sciences, Faculty of Medicine, Imperial College, London, UK; 4 TUM School of Medicine and Health, Institute of Experimental Oncology and Therapy Research, Technical University of Munich, Munich, Germany; 5 Department of Cardiovascular Sciences, University of Leicester, and National Institute for Health Research Leicester Biomedical Research Centre, Leicester, UK; 6 Collaborative Biorepository for Translational Medicine, Department of Surgery, University of Cambridge and NIHR Cambridge Biomedical Research Centre, Cambridge, UK; 7 Shantou University Medical College, Shantou, China; 8 Cardiovascular and Metabolic Disease Translational Research Programme, National University of Singapore, Singapore, Singapore; 9 Present Address: Division of Cardiovascular Medicine, Stanford University School of Medicine, Stanford, CA USA

**Keywords:** Epigenomics, Vascular diseases, Regulatory networks, Transcriptomics

## Abstract

Aberrant vascular smooth muscle cell (VSMC) homeostasis and proliferation characterize vascular diseases causing heart attack and stroke. Here we elucidate molecular determinants governing VSMC proliferation by reconstructing gene regulatory networks from single-cell transcriptomics and epigenetic profiling. We detect widespread activation of enhancers at disease-relevant loci in proliferation-predisposed VSMCs. We compared gene regulatory network rewiring between injury-responsive and nonresponsive VSMCs, which suggested shared transcription factors but differing target loci between VSMC states. Through in silico perturbation analysis, we identified and prioritized previously unrecognized regulators of proliferation, including RUNX1 and TIMP1. Moreover, we showed that the pioneer transcription factor RUNX1 increased VSMC responsiveness and that TIMP1 feeds back to promote VSMC proliferation through CD74-mediated STAT3 signaling. Both RUNX1 and the TIMP1–CD74 axis were expressed in human VSMCs, showing low levels in normal arteries and increased expression in disease, suggesting clinical relevance and potential as vascular disease targets.

## Abstract (web summary)

Lambert, Oc et al. reconstruct gene regulatory networks from single-cell transcriptomics and epigenetic profiling, compare mouse and human data, and report previously unrecognized regulators of vascular smooth muscle cell proliferation in disease.

## Main

Vascular smooth muscle cell (VSMC) proliferation underlies cell accumulation in atherosclerotic artery disease and vascular restenosis, which occurs following stenting and grafting. While genetic studies point to VSMCs as important hereditary determinants of cardiovascular disease^1–3^, their clinical targeting is presently unexplored. VSMC lesion investment in experimental atherosclerosis results from extensive clonal expansion of a small number of cells^4,5^. Similar VSMC oligoclonality has been shown in other vascular disease models using genetic VSMC lineage tracing^6,7^, and clonal VSMC contribution in human disease has been proposed^8,9^. Modification of VSMC clonality by altered cell–cell communication^10^ and aging^11^ suggests that changes in activation frequency could underlie increased vascular disease risk.

Single-cell RNA-sequencing (scRNA-seq) studies in mouse models and human vascular disease have shown the remarkable transcriptional heterogeneity of VSMC-derived cells^12^. VSMCs can adopt states that range from quiescence, with high expression of contractile genes (such as *MYH11* and *ACTA2*), in healthy arteries, to cells that have induced signatures of other cell types, including fibromyocytes (for example, *TNFRSF11B*), macrophages (*LGALS3*) and chondrocytes (*RUNX2*) in addition to extracellular matrix (ECM) proteins and remodellers that are characteristic of the so-called synthetic state generated by classical VSMC phenotypic switching^13–16^.

Functional genomic analyses in lineage-traced animals have shed light on genetic determinants and mechanisms regulating VSMC state changes in disease^1,2,17^. These experimental approaches have also revealed unexpected regulators of VSMC investment, such as the pluripotency factor OCT4 (ref. ^18^), and identified a mesenchymal VSMC-derived state marked by expression of vascular cell adhesion protein 1 (VCAM1) and stem cell antigen 1 (SCA1)^13,16^. SCA1-positive VSMCs are found infrequently in healthy arteries but more abundantly in disease models, and have been linked to VSMC priming and proliferation^14,16,19,20^. This molecular heterogeneity may explain the apparent distinct effects of VSMC regulators in different contexts^21,22^. However, the events governing activation of clonal VSMC proliferation are understudied^23^.

The integration of scRNA-seq data with epigenetic information led to the identification of factors regulating developmental processes and disease^24^. Here we use this approach to model gene regulatory networks upon acute vascular injury when VSMC proliferation initiates. We find differential use of transcription factors in distinct VSMC transcriptional states along a proliferation-associated trajectory, possibly explaining context-specific effects of VSMC regulators. The analysis identifies known and candidate regulators that are prioritized using in silico simulation analysis. We functionally implicate Runt-related transcription factor 1 (RUNX1) and Tissue Inhibitor of Metalloproteinases-1 (TIMP1) in driving the activation of VSMC proliferation and suggest that these factors also operate in human disease development.

## VSMC activation is associated with de novo chromatin opening at distal sites linked to vascular-disease-associated genes

**Fig. 1:**
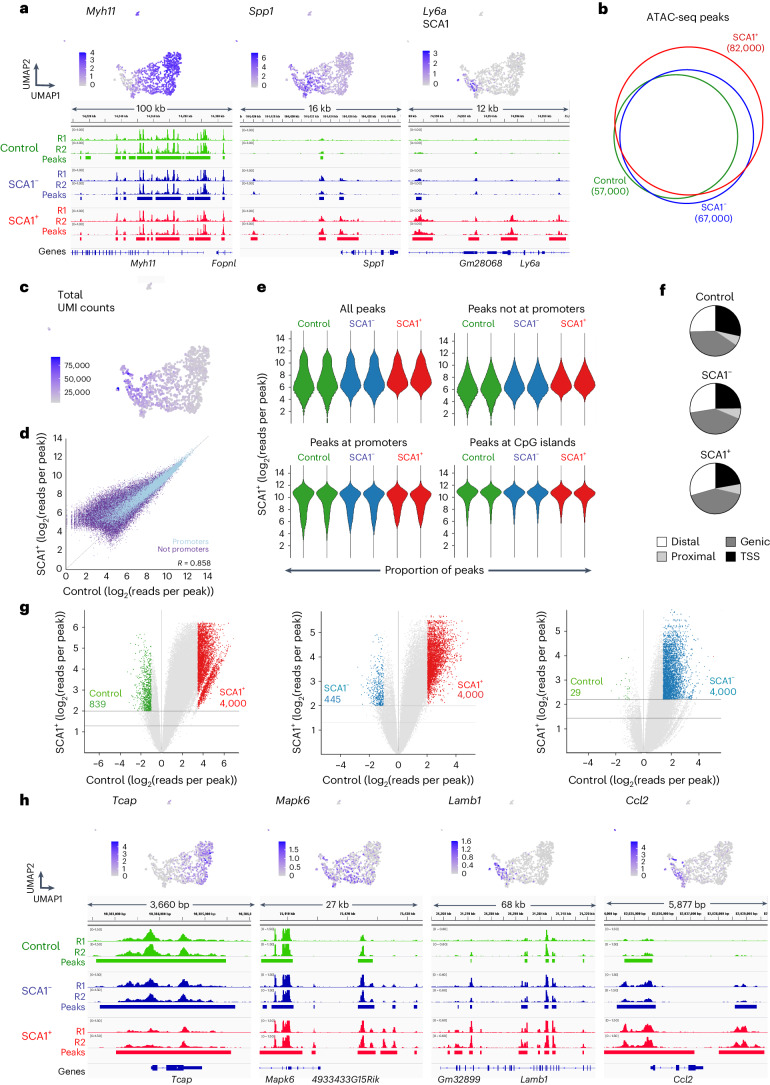
Activation of VSMCs results in widespread epigenetic activation of distal elements relevant for VSMC function and disease. **a**, ATAC-seq traces for markers of contractile VSMCs (*Myh11*), the synthetic phenotype (*Spp1*) and a VSMC transition state (*Ly6a*, encoding SCA1) in EYFP^+^ lineage-traced VSMCs isolated from healthy control Myh11–EYFP animals (green), and either EYFP^+^SCA1^−^ (SCA1^−^, blue) or EYFP^+^SCA1^+^ (SCA1^+^, red) cells isolated from injured arteries. Aligned reads for independent experiments with cells from different animals (R1 and R2) and reproducible peaks (horizontal bars) are shown. Uniform manifold approximation and projections (UMAPs, top) show associated normalized, log-transformed gene expression levels (counts/cell) in scRNA-seq profiles of VSMC-derived cells 7 days after injury (GSE162167). **b**, Venn diagram representing the overlap of ATAC-seq peaks in the three sample types. **c**, UMAP (as in **a**), showing the number of detected transcripts (unique molecular identifiers (UMI)) per cell (counts/cell). **d**, Scatterplot showing the read density in control (*x*-axis) and SCA1^+^ VSMCs after injury (*y*-axis) for ATAC-seq peaks at promoters (light blue) and peaks that are not at promoters (purple). **e**, Violin plots showing distribution of peak read densities in each sample for all peaks, or only non-promoter, promoter and CpG island peaks. **f**, Pie charts showing the proportion of peaks within 1 kb of TSSs, at promoter proximal regions (proximal), within genes (genic) or at intergenic elements (distal). **g**, Volcano plots showing the fold change of the peak intensity (differential accessibility (DA)) between indicated samples. Horizontal lines indicate significance thresholds of *P*_adj_ < 0.01 (top) and *P*_adj_ < 0.05 (bottom). Differentially accessible peaks used for pathway enrichment analysis are highlighted (fold change > 2 and of *P*_adj_ < 0.01, or the top 4,000 ranked by fold change for SCA1^+^ cells (LIMMA-modified *t*-test, two sided). The total number of peaks with increased accessibility in SCA1^+^ cells is 28,000 versus the control and 14,000 versus SCA1^−^). **h**, Normalized, log-transformed gene expression (counts/cell) and ATAC-seq data tracks (as in **a**) for genes showing higher accessibility in control samples (*Tcap*) or SCA1^+^ cells after injury (*Mapk6*, *Lamb1*, *Ccl2*). Source data

**Extended Data Fig. 1:**
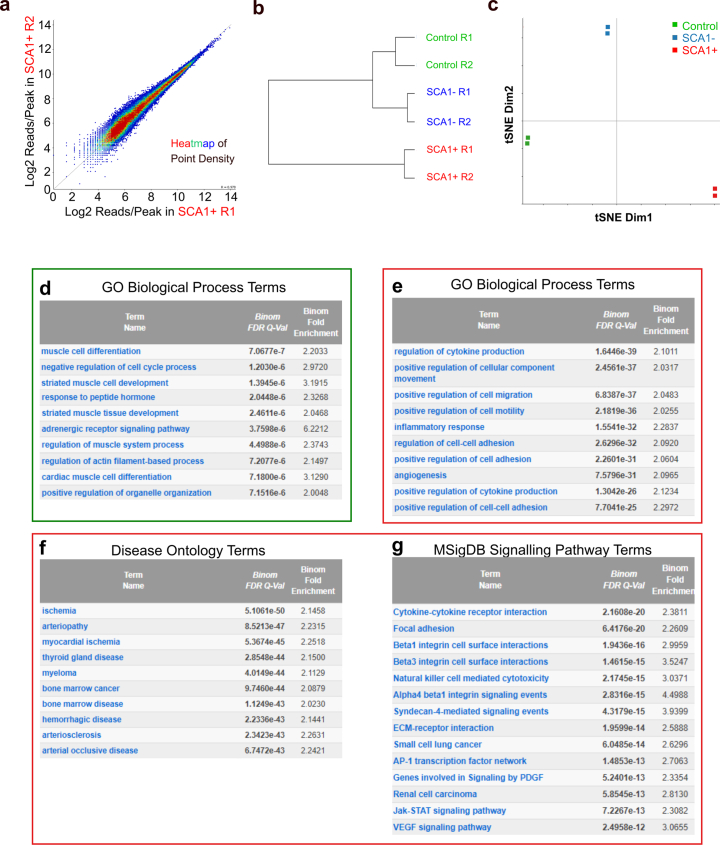
Quality assessment of ATAC-seq data and gene ontology analysis of differentially accessible genes. **a**, Scatter plot for two independent biological replicates of ATAC-seq profiling for SCA1+ cells showing high correlation between replicates. Colors show point density on a blue (low)-to-red (high) scale. **b-c**, Hierarchical clustering (**b**) and principal component analysis (**c**) of ATAC-seq samples, confirming similarity of replicate samples for all conditions. **d**, Significantly enriched gene ontology (GO) biological process terms for genes associated with peaks that are more accessible in the control samples from healthy animals, compared to SCA1+ cells isolated from injured arteries. **e-g**, Significantly enriched GO biological process (**e**), disease (**f**) and signaling pathway terms (**g**) for genes associated with peaks that are more accessible in SCA1+ cells isolated from injured arteries compared to the control samples from healthy animals. Significance was assessed with binomial testing (binom) in the GREAT package (http://great.stanford.edu/public/html/). Source data

To investigate the molecular regulation of VSMC proliferation, we elicited an injury response by carotid artery ligation, which leads to reproducible VSMC phenotypic switching and proliferation after 5–7 days^25^. Surgery was performed in Myh11–CreERt2, Rosa26–EYFP (Myh11–EYFP) animals after tamoxifen induction of heritable reporter-EYFP expression in VSMCs to overcome the rapid loss of VSMC marker expression after injury. VSMCs expressing SCA1 have increased proliferative capacity and represent cells with pronounced phenotypic switching at this stage of injury^13,16,20^. We therefore mapped chromatin accessibility using the assay for transposase-accessible chromatin with sequencing (ATAC-seq) in populations of SCA1^+^ lineage-traced (EYFP^+^) VSMCs from animals 7 days after surgery and compared it with the chromatin accessibility of EYFP^+^ VSMCs from no-injury littermate controls. We also profiled SCA1^−^ cells from injured arteries, which include VSMCs in remodeled areas that show less extensive phenotypic changes and cells in adjacent carotid artery regions without obvious injury responses. Samples from all three conditions (control, injury SCA1^+^ and injury SCA1^−^) had a high signal-to-noise ratio and strong replication of peak intensity (Fig. 1a and Extended Data Fig. 1a–c).

Accessibility changes were minor at *Myh11* and other contractile genes that are downregulated after injury (Fig. 1a), consistent with the documented retention of the active H3K4me2 marker at contractile genes in synthetic VSMCs^26,27^. Increased accessibility was found at, for example, *Spp1*, which is induced in both SCA1^+^ and SCA1^−^ VSMCs, whereas the chromatin at the SCA1-encoding *Ly6a* locus became more accessible selectively in SCA1^+^ cells (Fig. 1a). Of all peaks detected across the three conditions, 96% were accessible in SCA1^+^ cells (Fig. 1b). This genome-wide de novo chromatin opening in SCA1^+^ (82,000 peaks) compared with that in control samples (57,000) was consistent with the scRNA-seq transcript levels, which gradually increase across the VSMC population after injury (Fig. 1c).

ATAC-seq signal intensity was also generally higher in SCA1^+^ samples than in control VSMCs (Fig. 1d), in particular at regions with lower accessibility, which is a characteristic of enhancers. Consistently, the ATAC-seq peak intensity was shifted upward for non-promoter peaks, but not changed for promoters and CpG islands, and the proportion of peaks at distal elements was also higher in SCA1^+^ cells than in control samples (Fig. 1e,f). A significantly higher signal intensity was detected at 28,303 peaks in SCA1^+^ samples compared with control VSMCs, whereas only 839 peaks were higher in controls compared with SCA1^+^ (Fig. 1g). The SCA1^−^ sample showed intermediate results, possibly reflecting the heterogeneity of the cell population and the continuum of VSMC transcriptional states induced by injury^20^. Genes associated with regions selectively assessible in control cells were enriched for muscle contraction gene ontology (GO) terms (for example, *Tcap*; Fig. 1h and Extended Data Fig. 1d). By contrast, genes associated with the regions with the biggest chromatin accessibility increase in SCA1^+^ compared with control samples were enriched for vascular diseases, including atherosclerosis, and biological process ontology terms linked to VSMC proliferation, such as regulation of signal transduction (*Mapk6*), inflammation (*Ccl2*) and cell adhesion (*Lamb1*; Fig. 1h and Extended Data Fig. 1e–g). This analysis shows that injury-induced VSMC activation results in the opening of chromatin at enhancers of genes related to the functions of synthetic VSMCs and vascular disease, particularly in SCA1-expressing VSMCs.

## Rewiring of factors shared between injury-induced VSMC states

**Fig. 2:**
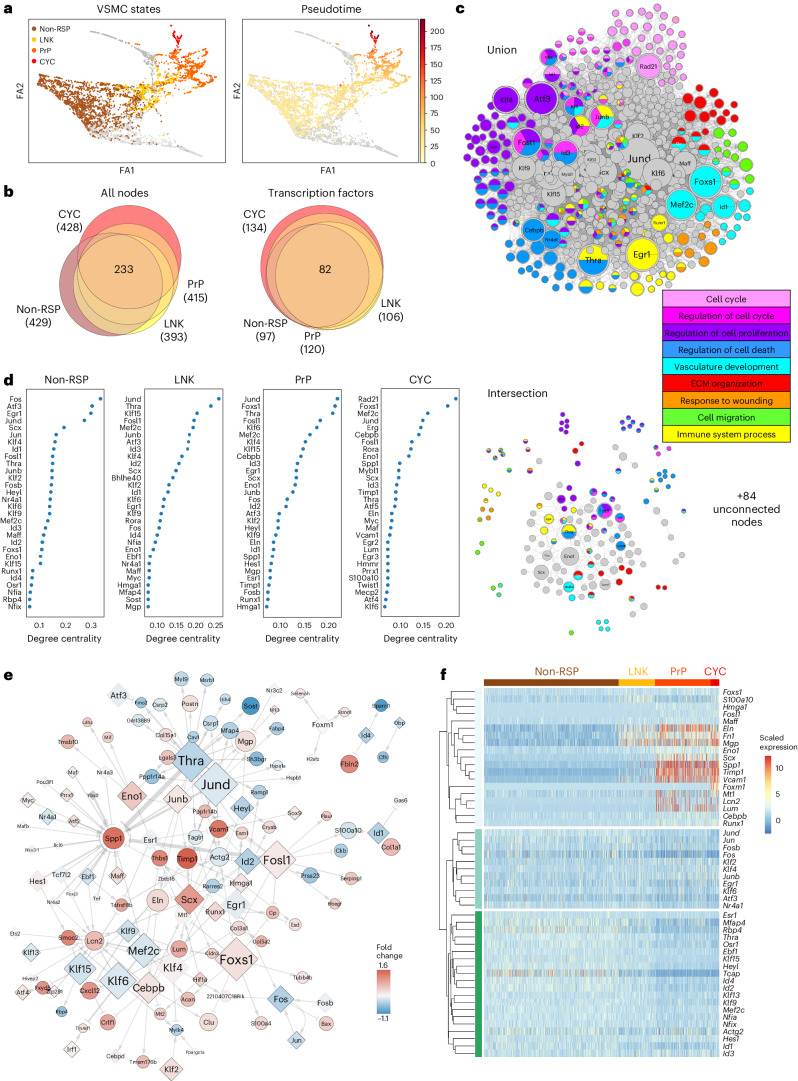
VSMC state-specific GRNs. **a**, Force-directed graph representation of scRNA-seq data from VSMC-derived cells isolated 5 days after injury (GSE162167) shaded by VSMC states (left; gray, non-assigned) or proliferation-associated pseudotime (right; yellow (low) to red (high) color scale, arbitrary units). FA: ForceAtlas2 dimension. **b**, Euler diagrams for all nodes and transcription factors in the four GRNs color coded as in **a**. The total number of nodes or transcription factors for each GRN is shown in brackets. **c**, The union and intersection of non-RSP, LNK, PrP and CYC networks colored by associated GO terms. The symbol size reflects node degree centrality scores separately in the union and intersection network. **d**, Nodes with top (30) degree centrality scores in each GRN. **e**, Network interactions in the PrP GRN with connectivity score > 0.1. Differential expression (fold change in log_2_ scale) between PrP and non-RSP cells is indicated by a blue (higher in non-RSP) to red (higher in PrP) color scale, white denotes 0. Black borders indicate significant differential expression (*P*_adj_ < 0.05, two-sided Wilcoxon rank-sum test). Edge widths reflect connectivity magnitude. **f**, Heat map showing scaled expression for the genes with the 50 highest rewiring scores for PrP versus non-RSP GRNs. Supplementary Fig. 4 shows panels **d**–**f** at enlarged size. Source data

**Extended Data Fig. 2:**
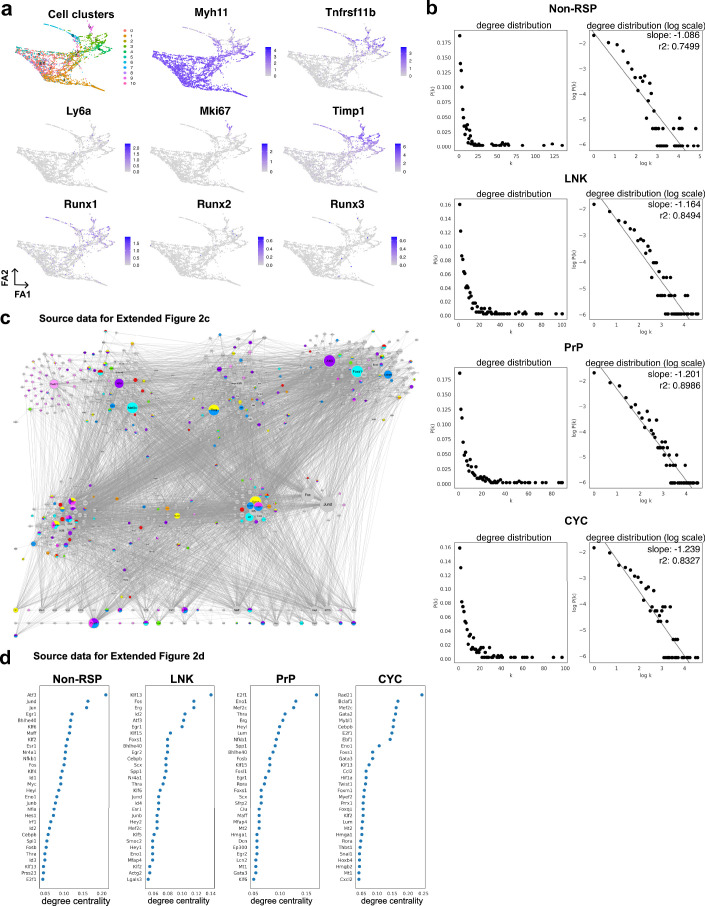
GRN analysis supplement. **a**, Force-directed graph of scRNA-seq profiles of VSMCs analyzed 5 days after injury showing the cell clusters and normalized, log-transformed expression of markers characteristic of different VSMC states and selected genes. **b**, Degree distributions for constructed GRNs. **c**, Community detection in the union of all four networks, coloured by gene ontology terms as in Fig. 2c. **d**, Nodes with top (30) degree centrality scores for the equivalent GRNs constructed with scRNA-seq profiles of VSMCs analyzed 7 days after injury. Supplementary Fig. 6 shows panel **d** at enlarged size. Source data

To identify factors controlling activation of VSMC proliferation, we compared how gene expression is orchestrated in VSMCs that respond to injury versus those that do not. We used scRNA-seq profiles of VSMCs analyzed at the very onset of VSMC proliferation (5 days after the injury), in which we previously identified a proliferation-associated trajectory^20^. VSMCs were classified based on their position along this trajectory as ‘non-responding’ (‘non-RSP’, defined by persistently high levels of contractile gene expression), a ‘linking’ cell population (‘LNK’), leading on to a pre-proliferative state that includes cells expressing SCA1 (‘PrP’) and, finally, actively cycling cells that express *Mki67* and have high G2/S cell cycle phase scores (‘CYC’; Fig. 2a and Extended Data Fig. 2a).

To construct gene regulatory networks (GRNs) for these four VSMC states, we first generated a VSMC-specific transcription factor–target interaction network based on all detected ATAC-seq peaks, which was combined with gene expression profiles. This was done for each of the VSMC states (non-RSP, LNK, PrP and CYC), using CellOracle^24^, and the resulting four GRNs had comparable centrality score distribution (Extended Data Fig. 2b). We found a substantial overlap between the networks, with 233 out of 597 (37%) shared nodes, including 82 out of 140 (59%) transcription factors (Fig. 2b and Supplementary Table 1a,b). Despite the high number of common nodes, few interactions were present in all GRNs (Fig. 2c, lower panel). Accordingly, abundant changes in the network topology rankings, such as degree centrality, were observed between networks (Fig. 2d and Supplementary Table 1c). GRN modeling using the scRNA-seq profiles of VSMCs analyzed 7 days after injury was conducted in parallel and produced comparable results (Extended Data Fig. 2d).

The network nodes were enriched for processes related to VSMC biology or vascular injury, including ECM organization, actin organization, response to stimuli and regulation of cell proliferation (Fig. 2c and Supplementary Table 1b). Overall, specific regulatory circuits driving biological processes were not detected (Extended Data Fig. 2c). However, the CYC network included a substructure consisting of cell-cycle-associated genes regulated by RAD21, a subunit of the cohesion complex that has roles in mitosis, genome stability and transcriptional regulation (Extended Data Fig. 2b,c). The CYC network, which had the most unique factors (14 transcription factors; Fig. 2b), was dominated by genes driving cell cycle progression, such as *Myc*, *Mybl1*, *Erg* and *Rad21* (Fig. 2d). To study processes and factors driving activation, rather than progression, of VSMC proliferation, we focused on comparison to the PrP network (Fig. 2e).

Genes with most network changes in the PrP relative to the non-RSP network were predominantly transcription factors (36 out of 50) and fell into three groups by expression pattern (Fig. 2f). Group 1 increased expression along the injury-associated trajectory, and genes included markers of VSMC phenotypic switching (*Spp1*, *Fn1*, *Mgp*), *Vcam1* (that marks activated VSMCs^13^), *Timp1* and transcription factors *Scx*, *Cebpb*, *Runx1* and *Eno1*. Group 2 genes, which showed reduced expression along the trajectory, included *Mef2c*; *Thra*; *Hes*; *Heyl*; Krüppel-like factors (KLF) 9, 13 and 15; and inhibitor of differentiation (Id) genes. Finally, group 3 genes showed little variation in expression between the non-responding and proliferation-associated states. This group contained factors reported to affect VSMC regulation, for example, *Klf4*, *Egr1*, *Atf3* and AP-1 subunits. We identified significant colocalization events between human VSMC expression quantitative trait loci (eQTLs) and signals in genome-wide association studies for coronary artery disease (CAD) for 13 human orthologs of the 50 genes with the highest rewiring scores (for example, *KLF2/4/13/15*, *HEYL*, *TCAP*, *S100A10*, *VCAM1*; Supplementary Table 1d,e), indicating causality of VSMC regulatory rewiring in disease.

## Injury-induced GRNs are relevant in atherosclerosis

**Fig. 3:**
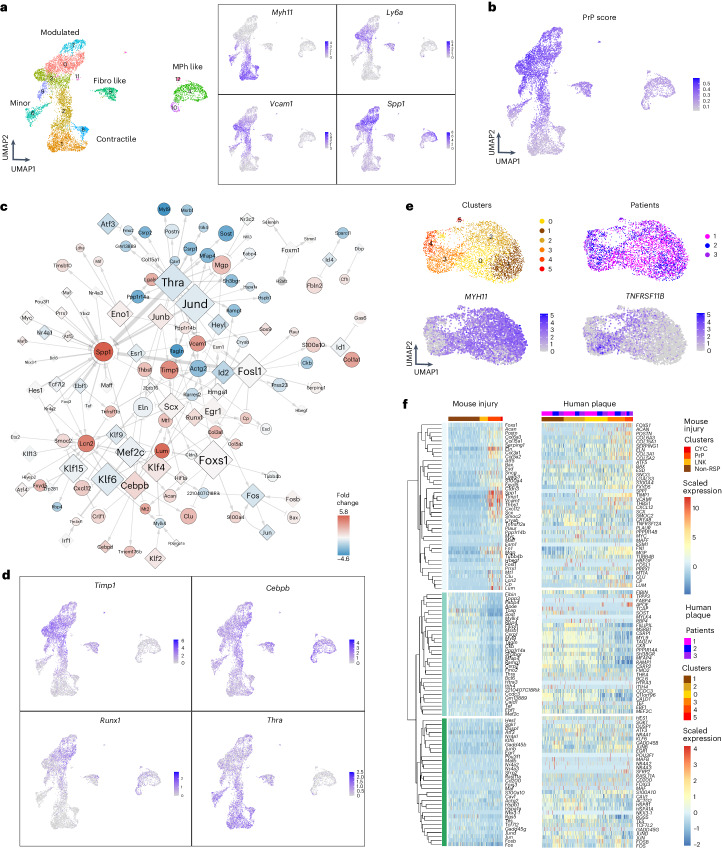
GRN rewiring after injury aligns with gene expression changes in human and mouse atherosclerosis. **a**–**d**, Analysis of GRN node expression in experimental atherosclerosis using scRNA-seq profiles of lineage-traced VSMCs from *Apoe*-null animals (GSE155513) fed a high-fat diet. **a**, UMAPs showing cell clusters and annotation (left) and normalized, log-transformed expression (counts/cell) of VSMC state marker genes (right). **b**, UMAP (as in **a**) showing the UCell enrichment scores (arbitrary units) of the PrP signature genes (increased expression in PrP versus non-RSP cells, log_2_ fold change (FC) > 0.5, *P*_adj_ < 0.05, two-sided Wilcoxon rank-sum test). **c**, The PrP network, as shown in Fig. 2e, but the shading of nodes represents differential expression (fold change in log_2_ scale) between modulated and contractile VSMCs in the atherosclerosis mouse model (blue, higher in contractile VSMCs (cluster 1); red, higher in modulated VSMCs (clusters 0 + 3); white denotes 0). Black borders indicate significant differential expression (*P*_adj_ < 0.05, two-sided Wilcoxon rank-sum test). **d**, UMAP (as in **a**) showing normalized, log-transformed expression (counts/cell) for selected genes with high rewiring scores. **e**,**f**, Analysis of GRN node expression in VSMCs from a human carotid plaque scRNA-seq dataset (GSE155512). **e**, UMAPs annotated with cell clusters, patients and normalized, log-transformed expression (counts/cell) of contractile (*MYH11*) and modulated VSMC genes (*TNFRSF11B*). **f**, Heat map showing scaled expression of genes ranked in the top 10 according to rewiring score (PrP versus non-RSP GRNs), and their direct strong interactors (connectivity score > 0.1), in human carotid plaque VSMCs (right) and in the mouse injury dataset (left). Genes are clustered based on correlated expression along the mouse injury VSMC trajectory (left). Supplementary Fig. 5 shows panels **c** and **f** at enlarged size. Source data

**Extended Data Fig. 3:**
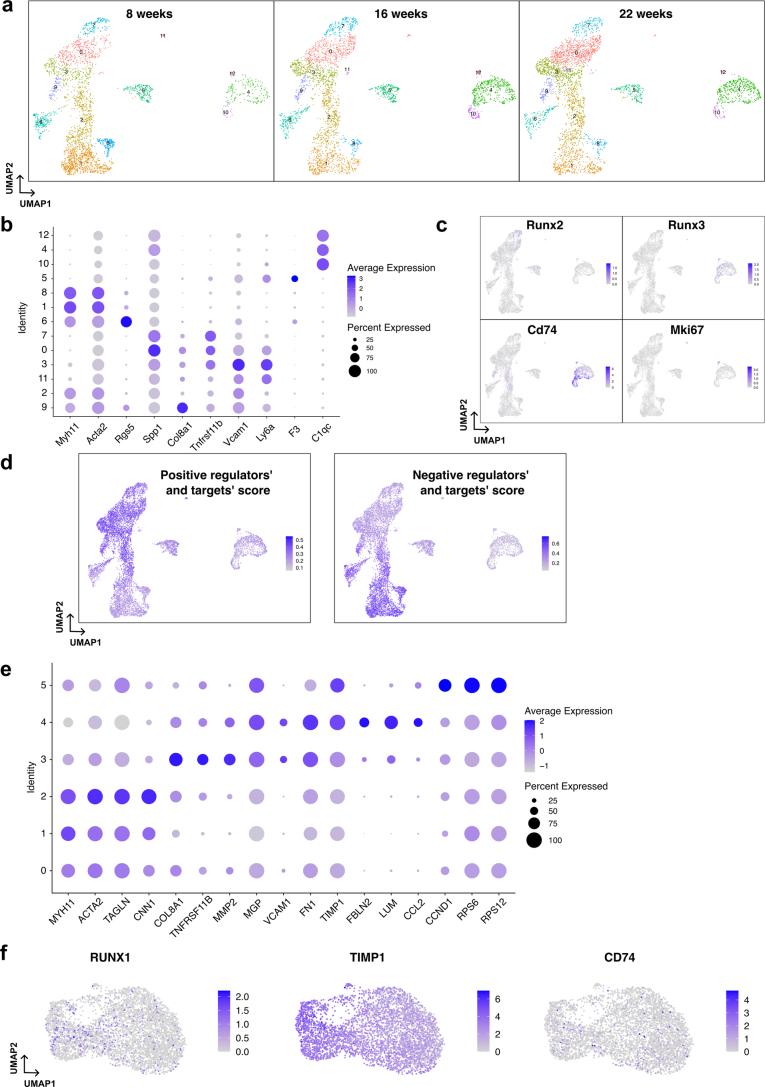
Gene expression changes of GRN nodes in human and murine atherosclerosis scRNA-seq datasets. **a-d**, Analysis of scRNA-seq profiles of VSMC lineage-traced cells from a murine atherosclerosis model (GSE155513). **a**, UMAP showing cluster annotation for cells isolated after 8, 16 or 22 weeks. **b**, Dot plot showing expression across VSMC clusters for contractile genes (*Myh11*, *Acta2*), markers of ‘minor SMCs’ (*Rgs5*, Pan et al., 2020), genes expressed by synthetic VSMCs (*Spp1*, *Col8a1*, *Tnfrsf11b*), a VSMC transition state (*Vcam1*, *Ly6a*) and fibroblast-like (*F3*), fibrochondrocyte (*Sox9*) or macrophage-like (*C1qc*) VSMCs. Scaled expression levels are shown on a gray-to-blue scale and dot size represent the percentage of expressing cells. **c-d**, UMAP showing normalized, log-transformed expression of indicated genes (**c**) or the UCell signature enrichment scores for the 10 top trajectory-stimulating (Positive regulators) or trajectory-blocking (Negative regulators) transcription factors (ranked by perturbation score), and associated direct target genes (positive interactions, connectivity score>0.1, **d**). **e-f**, Analysis of VSMC profiles from human carotid plaque scRNA-seq dataset (GSE155512). **e**, Dot plot showing gene expression across VSMC subclusters for contractile VSMC markers (*MYH11*, *ACTA2*, *TAGLN*, *CNN1*), genes associated with phenotypically modulated VSMCs (*COL8A1*, *TNFRSF11B*, *MMP2*, *MGP*, *VCAM1*) and genes associated with different VSMC clusters (*CCL2*, *RPS6*, *RPS12*). Scaled expression levels are shown on a gray to blue scale and dot size represent the percentage of expressing cells. **f**, Normalized, log-transformed expression of selected genes is shown on a gray-blue scale on a UMAP.

To assess the relevance of the regulation detected in injury to disease-associated VSMC changes, we used available datasets from VSMC-derived cells in experimental atherosclerosis^13^. First, we considered genes with higher expression in the PrP compared with the non-RSP state in injury. Expression of this ‘PrP signature’ was increased in VSMC subsets characterized by reduced expression of contractile genes (Fig. 3a,b and Extended Data Fig. 3a). Secondly, we found substantial overlap in transcript regulation between the two disease models for GRN nodes. Differential expression between contractile (cluster 1) and modulated (clusters 0 + 3) VSMC clusters in atherosclerosis was visualized on the PrP network (Fig. 3c), revealing a similarity to the observed changes in injury (Fig. 2e). In general, transcription factors with increased expression in modulated VSMCs in atherosclerosis had positive connections to upregulated target genes in the PrP-GRN (for example, *Sox9-Vcam1*, *Eno1-Tmsb10/Lgals3*, *Cebpb-Clu*). Vice versa, transcription factors with reduced expression in modulated versus contractile VSMCs in atherosclerosis, such as THRA, had positive GRN interactions with genes that were also downregulated in modulated cells (for example, *Csrp2*, *Mfap4*, *Sost*, *Myl9*; Fig. 3c). This analysis suggests similarity of the VSMC states in atherosclerosis and injury, and indicates that regulatory relationships mapped in injury are also relevant in atherosclerosis.

To compare with human disease, we isolated scRNA-seq profiles of mural cells from a published analysis of human carotid plaques^13^. The cells were clustered into six groups that were characterized by marker analysis (Fig. 3e and Supplementary Table 2b). Clusters 0–2 had abundant contractile gene expression, cluster 3 cells expressed *TNFRSF11B* that was identified as a fibromyocyte marker^14^, *VCAM1* was expressed mainly in clusters 3 and 4, and cluster 5 had increased levels of ribosomal proteins (Extended Data Fig. 3d,e). We then visualized the expression profiles for human orthologs of injury-rewired genes in the human cell clusters ordered by contractile gene expression. To capture regulatory relationships, we included the top 10 genes (by GRN rewiring score) and their direct interactors. Genes were visualized in the same order in human plaque and mouse injury cells (hierarchically clustered by their expression along the proliferation-associated trajectory; Fig. 3f). This side-by-side comparison showed similar expression patterns in mouse injury and carotid plaque VSMCs, indicating that the GRN analysis identified regulation that is relevant in human atherosclerosis.

## Prioritization of candidate regulators of VSMC activation

**Fig. 4:**
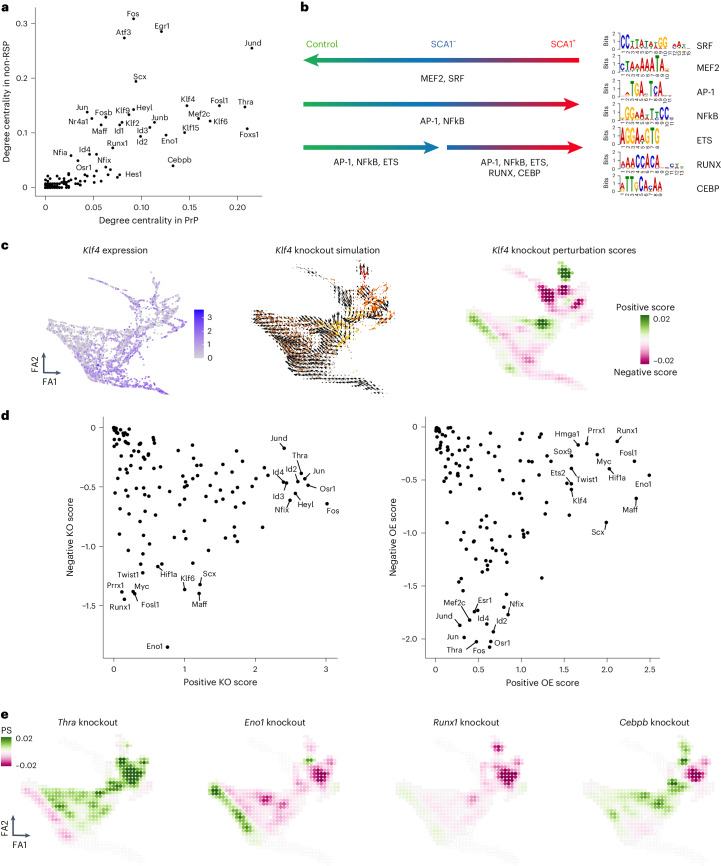
Rewiring of GRNs across cell states identifies candidate regulators of VSMC activation. **a**, Comparison of the out-degree centrality scores in PrP and non-RSP GRNs indicating top-scoring nodes. **b**, Summary of motif enrichment analysis for peaks showing higher accessibility relative to all peaks for the indicated comparisons (left). Detected motifs are shown on the right. **c**, Force-directed graph projections with shading showing normalized, log-transformed Klf4 expression (counts/cell) (left) and simulation vector field (middle; colors indicate the VSMC state as in Fig. 2a), or the PS values (right) as predicted by in silico knockout of *Klf4*. **d**, Sum of positive versus sum of negative PSs for systematic in silico KO (left) and OE (right) simulations. Top-ranked transcription factors are indicated. **e**, Force-directed graphs showing PSs from in silico simulation of knockout phenotypes for indicated transcription factors. Source data

**Extended Data Fig. 4:**
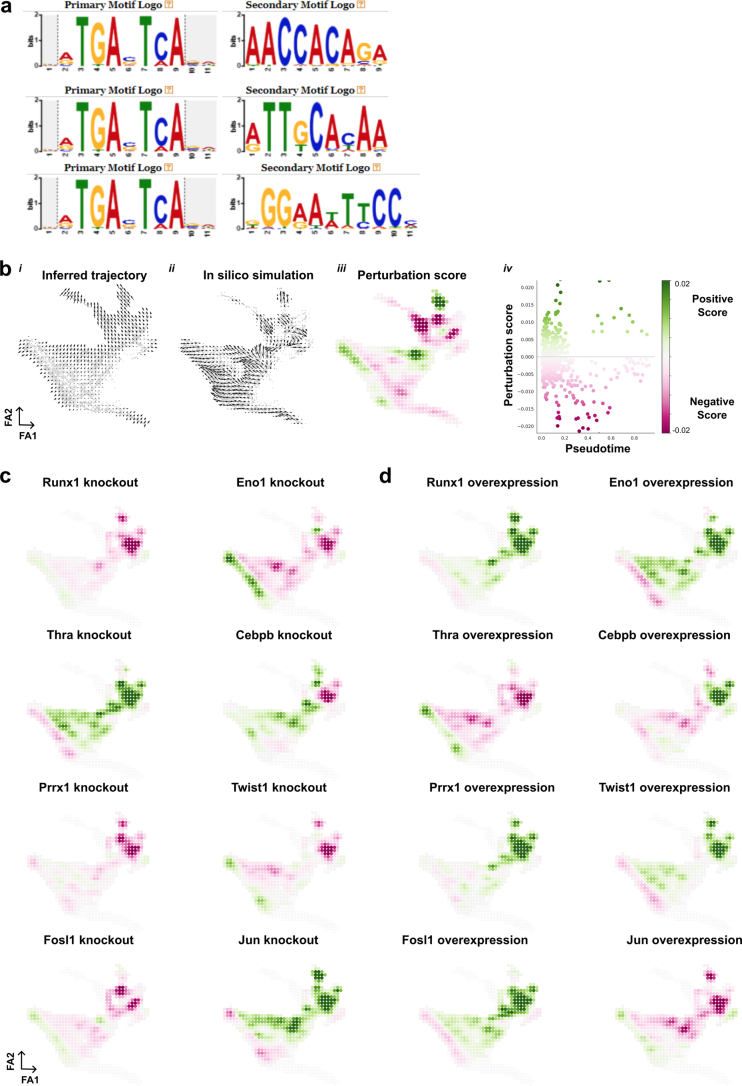
*In silico* network simulation results. **a**, The RUNX, CEBP and NFκB motifs were detected adjacent to the AP-1 motif in peaks showing increased accessibility in SCA1+ cells after injury compared to control VSMCs from no-surgery animals. **b**, Schematic explaining how perturbation scores were generated. Left panels show vector fields for inferred cell state trajectory (i) and results of *in silico* perturbation of transcription factor levels (ii) on a force directed graph representation. Right panels show the computed cell perturbation score on a force directed graph representation (iii) or as a function of pseudotime (iv). Negative (pink) and positive scores (green) were summarized separately for cells in the Non-RSP, LNK, PrP and CYC states. **(c, d)** Force-directed graph of VSMCs isolated 5 days after injury, showing perturbation scores from *in silico* knockout (KO, **c**) or overexpression (OE, **d**) simulation for the indicated transcription factors with the color-coding described in **b**.

Transcription factors changed topology scores between the GRNs (Fig. 4a), prompting a motif enrichment analysis for ATAC-seq peak regions showing differential accessibility between conditions (Fig. 4b). The MEF2 and SRF motifs were enriched for peaks showing higher accessibility in control samples compared with SCA1^+^ VSMCs from injured arteries. Peaks that gained de novo accessibility after injury were enriched for AP-1, NFκB, CEBP, ETS and RUNX binding sites. Interestingly, RUNX, NFκB and CEBP motifs were also found to colocalize with AP-1 binding sites (Extended Data Fig. 4a), suggesting co-regulatory interaction at some loci.

The generated GRNs enabled computational simulation of how perturbation of transcription factor levels would affect cell state^24^. KLF4, a known regulator of VSMC phenotypic switching that promotes a macrophage-like state in atherosclerosis^28^, had high centrality in all networks apart from that of proliferating cells (CYC). In silico simulation of KLF4 depletion predicted a general shift towards the non-responding cell state, concomitantly with promotion of CYC cells (Fig. 4c). Such context-specific effects of KLF4 depletion are consistent with the delayed downregulation of contractile genes, but accelerated neointima formation in VSMC-specific KLF4 knockout after injury animals^21^.

To systematically predict function, we generated in silico perturbation scores (PSs) for each transcription factor in the networks. Positive scores, reflecting stimulation of progression along the proliferation-associated trajectory^20^, and negative scores, indicating blocking effects, were calculated for loss of function (knockout, KO) and gain of function (overexpression, OE) separately and showed strong correlation (*R*^2^ > 0.9; Extended Data Fig. 4b and Supplementary Table 1g). Transcription factors scoring highly for both positive and negative PSs were predicted to have context-specific effects (Fig. 4d and Extended Data Fig. 4c,d). RUNX1, FOSL1, TWIST1 and PRRX1 were predicted to mainly stimulate trajectory progression (negative KO score < −1, positive KO score < 0.75 (Fig. 4d,e and Extended Data Fig. 4c,d)). The factors predicted to block trajectory progression (positive KO score > 1) were enriched for TGF-beta signaling (KEGG:04350), IL-17 signaling (KEGG:04657) and parathyroid hormone synthesis, secretion and action (KEGG:04928). Interestingly, thyroid hormone receptor alpha (THRA), which has been suggested to impact VSMC cholesterol metabolism^29^, was among trajectory-blocking factors (Fig. 4d). *Thra* is downregulated in PrP compared with non-RSP VSMCs and negatively interacts with synthetic genes (*Spp1*, *Vcam1* and *Mgp*) in the PrP network, while promoting cytoskeletal genes (*Actg2*, *Pdlim4*, *Cdc42ep3*) in the non-RSP GRN (Supplementary Table 1a). Simulation of THRA perturbation also suggested that THRA may safeguard the contractile state (Fig. 4e and Extended Data Fig. 4c–d).

Next, we used the GRN analysis to investigate how targets of this transcriptional rewiring may impact VSMC biology. To this end, we performed pathway analysis of the nodes with high target gene scores (‘in-degree centrality’) in the PrP or CYC GRNs that are also induced in PrP compared with non-RSP cells (Supplementary Table 1c,d). This highlighted GO terms including ECM organization (*Spp1*, *Vcam1*, *Cxcl2*, *Postn*) and regulation of cell adhesion (*Eln*, *Lum*, *Fbln2*). We also found modulators of proteolysis (*Timp1*, *Serpine1*, *Thbs1*) among the targets of rewired transcription factors, consistent with cellular niche remodeling by activated VSMCs.

Overall, this analysis highlights both known and putative VSMC regulators. To experimentally test factors highlighted by the GRN analysis, we selected RUNX1 as a candidate stimulating transcription factor, based on top ranking in the PS analysis combined with motif enrichment in de novo accessible chromatin, and TIMP1 as an example of a highly rewired target gene that, interestingly, was predicted to be indirectly induced by RUNX1.

## RUNX1 promotes VSMC proliferation

**Fig. 5:**
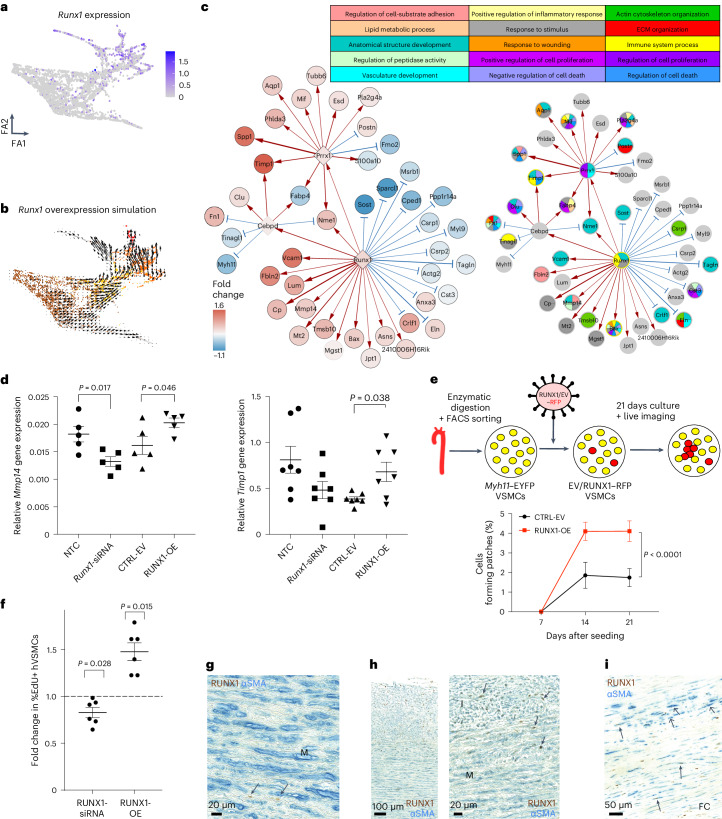
RUNX1-mediated regulation of VSMC proliferation. **a**,**b**, Force-directed graph of VSMCs isolated 5 days after injury showing normalized, log-transformed *Runx1* expression (counts/cell) (**a**) or the result of RUNX1 overexpression simulation (**b**; colors indicate VSMC states as in Fig. 2a). **c**, Direct and indirect targets of Runx1 in the PrP GRN showing differential expression between PrP and non-RSP cells on a blue (higher in non-RSP) to red (higher in PrP) scale where white denotes no change (left, black borders indicate significantly differential expression (fold change in log_2_ scale), *P*_adj_ < 0.05, two-sided Wilcoxon rank-sum test) or GO terms for nodes (right). Edge widths reflect connectivity magnitude, and colors show positive (red) and negative (blue) interactions. **d**, *Mmp14* and *Timp1* transcript levels detected by quantitative RT-PCR in lineage-labeled VSMCs transfected with non-targeting (NTC) or *Runx1*-targeting siRNA (*Runx1*-siRNA), or transduced with an empty vector (CTRL-EV) or RUNX1-overexpressing (RUNX1-OE) lentivirus. The symbols (circles, squares, triangles and inverted triangles) represent values from independent animals (*N* = 5), the lines represent means and the error bars represent s.e.m.; *P* value: two-tailed *t*-test or Mann–Whitney *U*. **e**, Schematic of clonal proliferation assay; RFP-expressing lentivirus is used to test the effect of RUNX1 cDNA relative to an empty control vector (EV) on the ability of lineage-labeled VSMCs from Myh11-EYFP animals to form colonies (left). The right panel shows the percentage of cells forming a clonal VSMC patch in RUNX1-OE and empty vector control cells (CTRL-EV). The points indicate mean values from individual animals (*N* = 4 animals analyzed in triplicate), the lines indicate means and the error bars represent s.e.m.; *P* = 1.97 × 10^−12^, generalized linear model**. f**, Fold change in the percentage of EdU^+^ cells after siRNA-mediated RUNX1 depletion (RUNX1-siRNA, relative to non-targeting siRNA-treated cells) and lentivirus-mediated RUNX1 overexpression in hVSMCs (RUNX1-OE, relative to cells transduced with empty vector virus). The points are the averages of quadruplicate replicates for independent hVSMC isolates (*N* = 6 donors), the lines indicate means and the error bars indicate s.e.m.; *P* value: two-tailed *t*-test. **g**–**i**, Representative immunostaining of non-plaque aorta (**g**,**h**, *N* = 10 donors) or carotid endarterectomy samples (**i**, *N* = 6 donors) for RUNX1 (brown) and αSMA (blue). FC, fibrous cap; I, intima; M, media. Examples of RUNX1^+^ cells are indicated by closed arrowheads, and examples of RUNX1^−^αSMA^+^ cells are indicated by open arrowheads. Source data

**Extended Data Fig. 5:**
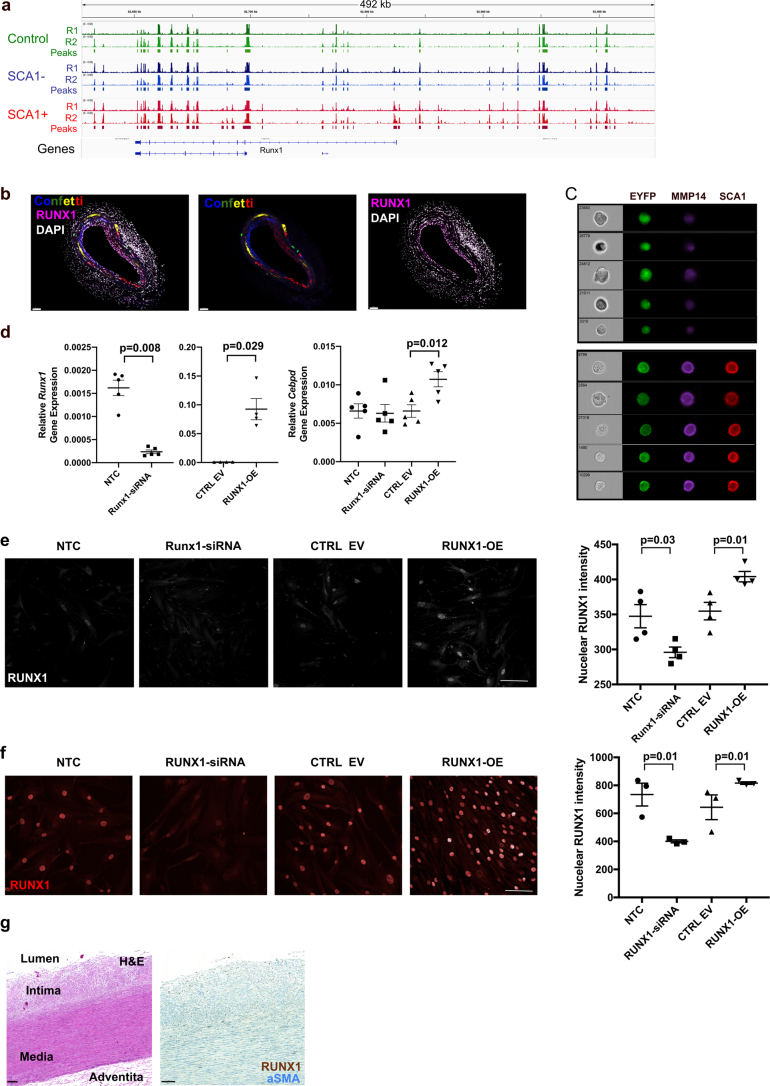
Analysis of RUNX1 as a candidate VSMC regulator. **a**, ATAC-seq data tracks for the *Runx1* locus, annotated as described in Fig. 1a. **b**, RUNX1 immunostaining in cryosections from the injured left carotid artery of a Myh11-Confetti animal, Scale bar= 50 µm. Representative of 2 animals. Signals for RUNX1 (magenta), nuclear DAPI (white) and confetti lineage-labeling are shown as indicated. **c**, Imagestream analysis of lineage-labeled EYFP+ (green) VSMCs from Myh11-EYFP animals stained for SCA1 (red) and MMP14 (magenta). Left columns show brightfield images. Representative of 3 animals. **d**, *Runx1* and *Cebpd* transcript levels detected by quantitative RT-PCR in mVSMCs transfected with non-targeting (NTC) or *Runx1*-targeting siRNA (Runx1-siRNA), or transduced with an empty vector (CTRL-EV) or RUNX1-overexpressing (OE) lentivirus. Dots show values for cells from independent animals (N = 5), lines mean, error bars SEM, p-value: two-tailed MWU (Runx1) or two-tailed *t*-test (Cebpd). **e-f**, Immunofluorescence staining of RUNX1 in mVSMCs (**e**) and hVSMCs (**f**) transfected with non-targeting (NTC) or Runx1-targeting siRNA (Runx1-siRNA), or transduced with an empty vector (CTRL-EV) or RUNX1-overexpressing (OE) lentivirus. Scale=100 µM. Graph shows quantification of nuclear RUNX1 signals. Dots show values for cells from individual animals (E, N = 4, p-value: two-sided MWU) or hVSMC isolates from different donors (F, N = 3, p-value: two-sided *t*-test), lines mean, error bars SEM, p-value: two-tailed *t*-test. **g**, Representative images (N = 5) of non-plaque aorta with intimal thickening showing sequential sections after H&E (left) or immunostaining for αSMA (blue) and RUNX1 (brown). Scale bars = 100 µm. Lumen, intima, media and adventitia are indicated. Source data

The GRN analysis highlighted RUNX1, which belongs to the Runt-related transcription factor family and plays a key role in hematopoiesis, as a potent stimulator of VSMC activation. Consistent with this, *Runx1* transcripts were increased in the PrP and CYC states compared with non-responding cells, increased chromatin accessibility was detected at both *Runx1* promoters in SCA1^+^ VSMCs compared with the control samples, and we detected RUNX1 in lineage-labeled VSMCs after injury (Fig. 5a and Extended Data Fig. 5a,b). By contrast, the RUNX family members *Runx2* and *Runx3* were lowly expressed at this stage of VSMC activation (Extended Data Fig. 2a). The RUNX motif was also highlighted in relation to VSMC regulation by studies in experimental atherosclerosis^1^, and *RUNX1* transcript levels were higher in modulated compared with contractile VSMCs in mouse and human atherosclerosis (Fig. 3d and Extended Data Fig. 3f).

GRN-based simulation of elevated RUNX1 expression predicted that RUNX1 promotes the transition towards VSMC proliferation (Fig. 5b), as expected from the limitation of proliferation observed when simulating *Runx1*-knockout (Fig. 4e and Extended Data Fig. 4c,d). The PrP GRN predicted that RUNX1 stimulates injury-induced genes, including *Vcam1*, *Lum* and *Mmp14*, and GO analysis of all direct and indirect RUNX1 target genes suggested that RUNX1 could affect cell-substrate adhesion (*Fbln2*, *Mmp14*), response to stimulus (*Cp*, *Mt2*) and ECM organization (*Eln*) (Fig. 5c). Interestingly, 4 of the 15 direct RUNX1 target genes were previously associated with activated, SCA1^+^ VSMCs in healthy arteries^16^. We also found that expression of the predicted RUNX1 target, matrix metalloproteinase 14 (MMP14), is a hallmark of SCA1^+^ VSMCs (Extended Data Fig. 5c), further indicating that RUNX1 supports a state that is primed for proliferation.

We experimentally tested how RUNX1 affects gene expression in freshly isolated lineage-traced VSMCs. Lentiviral-induced RUNX1 overexpression significantly increased the expression of predicted direct targets *Mmp14, Timp1* and *Cebpd*. Conversely, the depletion of *Runx1* mediated by short interfering RNA (siRNA) significantly reduced *Mmp14* and blunted *Timp1* expression (Fig. 5d and Extended Data Fig. 5d,e). To test whether RUNX1 affects activation of VSMC proliferation, we adapted an in vitro proliferation assay that reproduces the low-frequency clonal VSMC expansion observed in vascular disease models^20^. Lentivirus expressing red fluorescent protein (RFP) was introduced in lineage-labeled EYFP^+^ VSMCs at low titer and expanding RFP/EYFP double-positive clonal VSMC patches identified by repeated live cell imaging over 3 weeks. This showed a significantly increased clonal patch-forming ability of RUNX1-overexpressing versus control cells (Fig. 5e).

In human VSMCs (hVSMCs), RUNX1 depletion decreased the percentage of EdU^+^ cells and, vice versa, overexpression of RUNX1 increased the percentage of EdU^+^ cells, relative to their respective controls. This suggests that stimulation of VSMC proliferation by RUNX1 is conserved in humans (Fig. 5f and Extended Data Fig. 5f). To assess RUNX1 functions in human disease, we immunostained arteries without detectable plaques but showing varying degrees of intimal thickening and carotid plaques. RUNX1 protein was detected infrequently in alpha smooth muscle actin (αSMA)^+^ cells in arteries with a healthy, organized morphology of the medial layer, but a higher proportion of medial RUNX1^+^αSMA^+^ cells were observed in arteries with evidence of perturbation (Fig. 5g). For example, in arteries showing intimal thickening, many αSMA^+^ cells within the intima expressed RUNX1 (Fig. 5h and Extended Data Fig. 5g). Substantial heterogeneity in RUNX1 expression was observed in human atherosclerotic lesions; interestingly, however, αSMA^+^RUNX1^+^ cells were also observed in the fibrous cap (Fig. 5i). Jointly, these results show that RUNX1 regulates gene expression in VSMCs and promotes their proliferation, and indicates that RUNX1 is implicated in human disease development.

## TIMP1 promotes VSMC proliferation in an MMP-independent manner

**Fig. 6:**
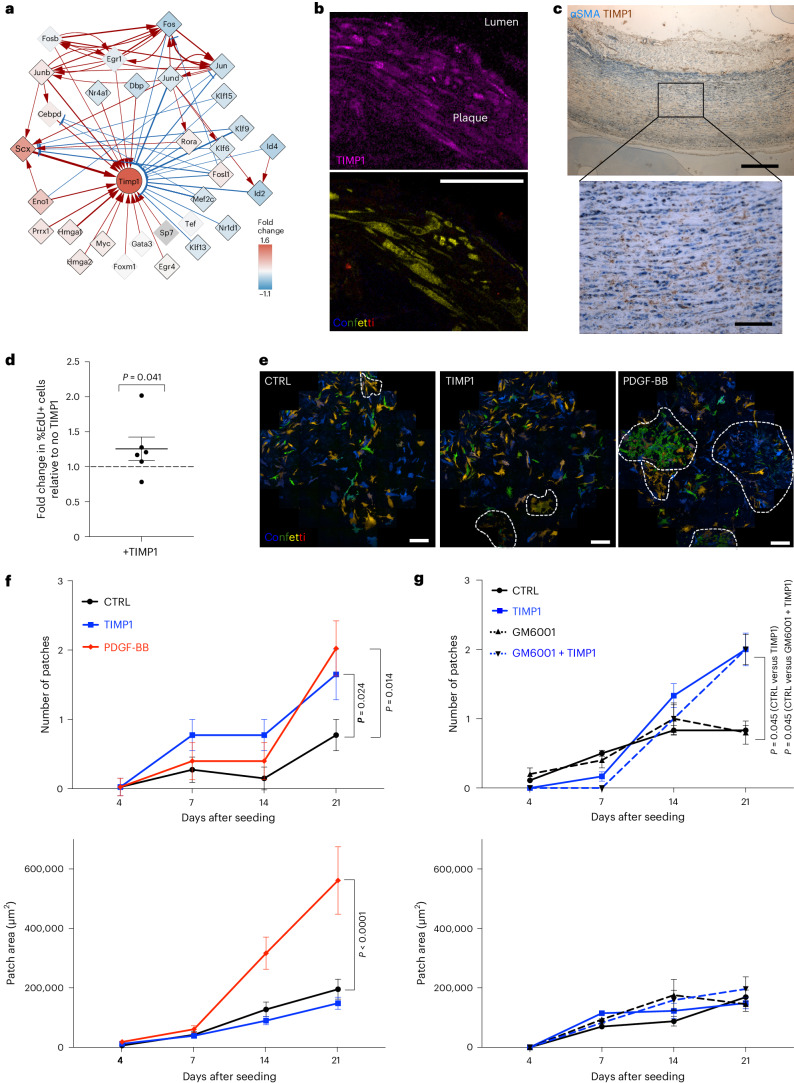
GRN analysis identifies TIMP1 as a functional gene target and driver of VSMC proliferation. **a**, TIMP1 connections in the PrP GRN annotated as in Fig. 5c. **b**, TIMP1 immunostaining of an atherosclerotic lesion from Myh11–Confetti/Apoe animals showing signals for fluorescent proteins (FP) in lineage-labeled VSMCs (Confetti: cyan (C)FP, blue; red (R)FP, red; yellow (Y)FP, yellow; green (G)FP, green), TIMP1 (magenta) in a single confocal z-section. Representative of three animals. Scale bar = 50 µm (applies to both panels). **c**, Representative immunohistochemistry image for αSMA (blue) and TIMP1 (brown) in non-plaque human aorta (*N* = 7 donors); scale bar = 500 µm (overview), 100 µm (zoomed view). **d**, Fold change in the percentage of EdU^+^ hVSMCs following 16 h of EdU incorporation in cells treated with 500 ng ml^−1^ rhTIMP1 relative to vehicle controls. Dots indicate the average of independent hVSMC isolates (*N* = 6 donors), the line indicates the mean and the error bars indicate the s.e.m.; *P* value: two-tailed *t*-test. **e**, Representative images of lineage-labeled VSMCs isolated from Myh11–Confetti aortas and cultured for 21 days in the presence of a vehicle control, 500 ng ml^−1^ recombinant mouse TIMP1 or 2 ng ml^−1^ PDGF-BB. Scale bars = 500 µm. **f**,**g**, Quantification of the number and size of clonally expanded patches of Confetti^+^ VSMCs over 21 days of culture. The points indicate the mean (*N* = 4 animals, triplicate analysis of cells from each animal), and the error bars indicate the s.e.m.; *P* = 5.2 × 10^−6^, generalized linear model. Source data

TIMP1 was among the most central nodes and was selected among the targets of regulatory rewiring as increased TIMP1 protein levels have been associated with cardiovascular disease and severity^30,31^. In the PrP GRN, TIMP1 was predicted to be induced by multiple factors including Cebpd, Myc, Prrx1 and Scx (Figs. 2e and 6a), and an indirect RUNX1 target (see above). TIMP1 transcripts were also upregulated by VSMCs in human and mouse atherosclerosis scRNA-seq datasets (Fig. 3d, Extended Data Fig. 3f and refs. ^1,13,16^).

**Extended Data Fig. 6:**
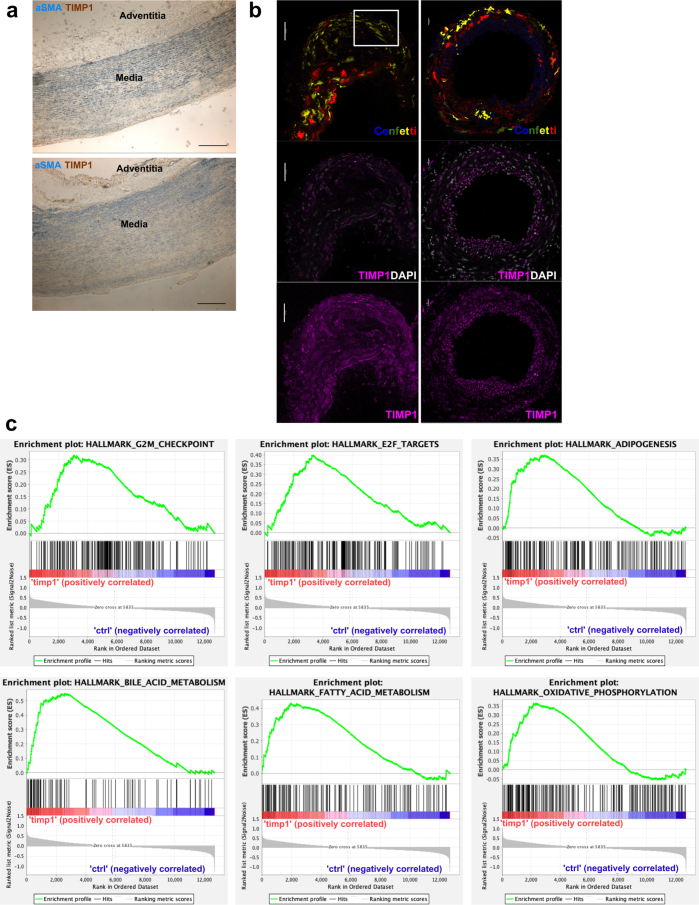
Impact of TIMP1 on VSMCs. **a**, Example immunohistochemistry images for α-smooth muscle actin (αSMA, blue) and TIMP1 (brown) in non-plaque human aorta. Scale bar=500 µm. N = 7 donors. **b**, Representative images of TIMP1 immunostaining in cryosections of an atherosclerotic plaque from Myh11-Confetti/Apoe animals fed a HFD (right, N = 3) or an injured Myh11-Confetti carotid artery (left, N = 3 animals). Signals for TIMP1 (magenta), nuclear DAPI (white) and confetti lineage-labeling (CFP: blue, RFP: red, YFP: yellow, GFP: green) are shown as indicated. White box indicate region shown in Fig. 6b. Scale bar = 50 μm (left), 20 μm (right). **c**, Enrichment plots from gene set enrichment analysis (GSEA) for E2F targets (p_adj_ = 0.005), G2M checkpoint genes (p_adj_= 0.04), adipogenesis (p_adj_ = 0.023) bile acid metabolism (p_adj_ = 0.00376), fatty acid metabolism p_adj_ = 0.00337) and oxidative phosphorylation (p_adj_ = 0.022) in bulk RNA-seq data from hVSMCs treated with 500 ng/mL recombinant human TIMP1 for 6 hours versus control cells (N = 6 independent human VSMC isolates/donors).

TIMP1 protein was expressed in clonally expanded VSMCs in both mouse atherosclerotic and injury-induced lesions, and also detected in αSMA^+^ cells located in the medial layer of non-plaque human arteries (Fig. 6b,c and Extended Data Fig. 6a,b). This suggested that TIMP1 could affect VSMCs at an early disease stage. To investigate the potential impact of TIMP1 protein on VSMC function, we performed bulk RNA-seq in TIMP1-treated versus control hVSMCs. This revealed a significant induction of cell cycle genes (E2F targets and G2/M checkpoint), fatty acid metabolism and oxidative phosphorylation gene sets in TIMP1-treated cells (Extended Data Fig. 6c), indicating an impact on metabolism and cell proliferation. We therefore tested the impact of TIMP1 on VSMC proliferation, as the role of TIMP1 is controversial^32–34^. We found a small, but significant, increase in the percentage of EdU^+^ cells in TIMP1-treated hVSMCs versus controls (Fig. 6d), similar to a recent study^34^. TIMP1 treatment also stimulated the clonal expansion of quiescent VSMCs isolated directly from aortas of lineage-labeled Myh11–Confetti animals (Fig. 6e,f). TIMP1 increased the frequency of VSMC clone formation to a similar degree as PDGF-BB treatment (Fig. 6f). However, TIMP1 did not change the size of clonal patches, in contrast to platelet-derived growth factor (PDGF)-BB (Fig. 6f, lower panel), suggesting that the establishment and growth of VSMC clones are regulated independently.

TIMP1 functions both as an MMP inhibitor and as a cytokine^35,36^. We found no effect of a broad-spectrum MMP inhibitor (GM6001) on clone dynamics, neither in control cells nor in TIMP1-treated cells (Fig. 6g). This suggested that the ability of TIMP1 to promote clonal VSMC proliferation is not linked to the inhibition of proteolysis, similar to established hVSMC cultures^32^. Taking all these results together, we conclude that TIMP1 stimulates clonal VSMC proliferation in an MMP-independent manner.

## TIMP1 induces STAT3 phosphorylation to induce VSMC proliferation

**Fig. 7:**
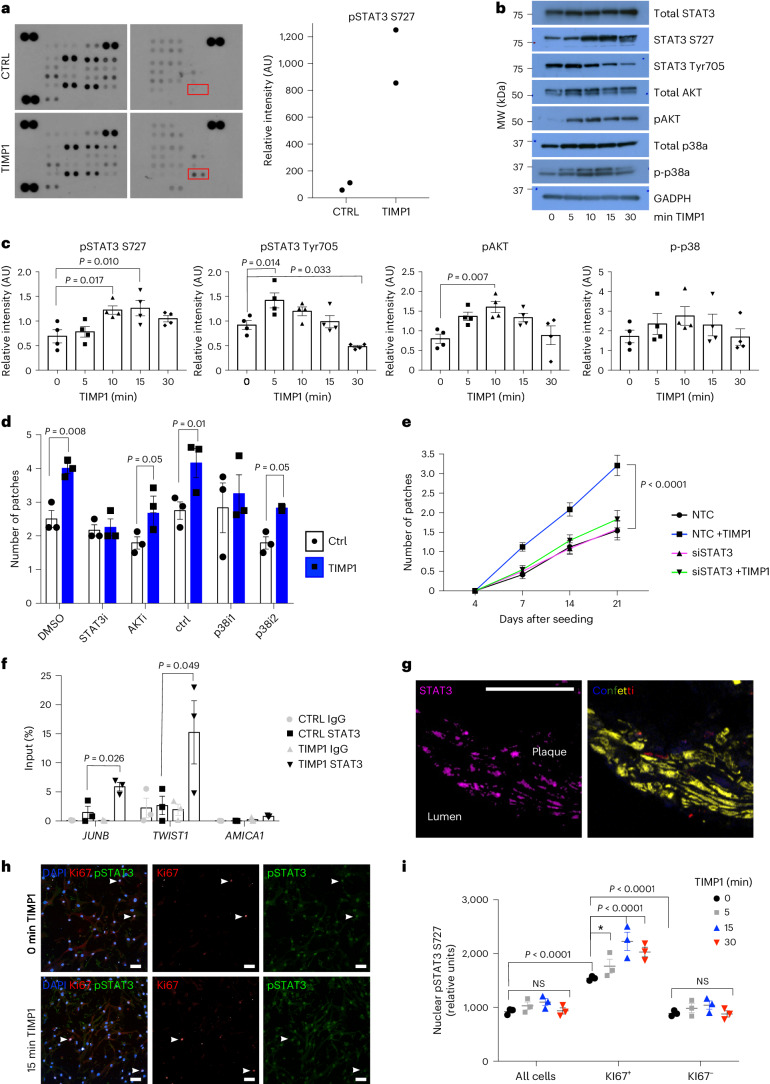
TIMP1 signaling induces STAT3 phosphorylation in human and mouse VSMCs. **a**, Phosphokinase array and densitometric quantification of serum-starved hVSMCs treated for 15 min with 500 ng ml^−1^ rhTIMP1 or vehicle control (*N* = 1 donor). **b**, Western blot of total STAT3, phospho-STAT3 (S727 or Tyr705), total AKT, phospho-AKT (pAKT), total p38a, phospho-p38a (p-p38a) and GAPDH in serum-starved hVSMCs after 0 min, 5 min, 10 min, 15 min and 30 min rhTIMP1. **c**, Quantification of relative western blot band intensity, normalized to GAPDH. The points show independent hVSMC isolates (*N* = 4), the bars indicate the means, and the error bars indicate s.e.m.; *P* value: one-way ANOVA. **d**, Number of clonally expanded patches formed by lineage-labeled VSMCs following 21 days of culture, without (white, circles) or with 500 ng rmTIMP1 (blue, squares) in samples treated with vehicle (DMSO), 10 µM TT101 (STAT3i), 100 nM MK-2206 (AKTi), 10 µM SB202474 (control inhibitor, ctrl), 10 µM SB203580 (p38i1) or 10 µM SB202190 (p38i2). The points show averages (*N* = 3 Myh11–Confetti animals analyzed in triplicate), the bars indicate means, the error bars indicated s.e.m.; *P* value: two-tailed *t*-test. **e**, Quantification over time of clonally expanded patches formed by lineage-labeled VSMCs, treated with non-targeting control (NTC) or *Stat3*-targeting siRNA (siSTAT3) ±500 ng rmTIMP1. The points indicate means (*N* = 3 Myh11–Confetti animals analyzed in triplicate), and the error bars indicated s.e.m.; *P* = 2.2 × 10^−7^, generalized linear model. **f**, ChIP–qPCR analysis at STAT3 targets (*TWIST* and *JUNB*) and negative control (*AMICA1*), in serum-starved control and rhTIMP1-treated hVSMCs (15 min, 500 ng ml^−1^), showing anti-STAT3 and control-IgG precipitated DNA as a percentage of the input. The bars show the means of independent hVSMC isolates (*N* = 3 donors), and the error bars indicate the s.e.m.; *P* value: two-tailed *t*-test. **g**, pSTAT3 S727 immunostaining (magenta) in cryosections from carotid plaque in an Myh11–Confetti/Apoe animal (11 weeks HFD) with Myh11–Confetti signals (CFP, blue; RFP, red; YFP, yellow; GFP, green). Representative of three animals. Scale bar = 50 µm (applies to both images in panel). **h**,**i**, pSTAT3 S727 and KI67 immunostaining (**h**) and quantification (**i**) of serum-starved control hVSMCs ±500 ng ml^−1^ rhTIMP1 treatment (15 min, **h**) or indicated time points (**i**). The arrowheads indicate KI67^+^ cells. Scale bars = 50 µm. The symbols show average values for independent hVSMC isolates (*N* = 3 donors, analyzed in triplicate), the lines represent means and the error bars represent s.e.m.; *P* value: two-way ANOVA, *P = 0.0498; NS, not significant. Source data

**Extended Data Fig. 7:**
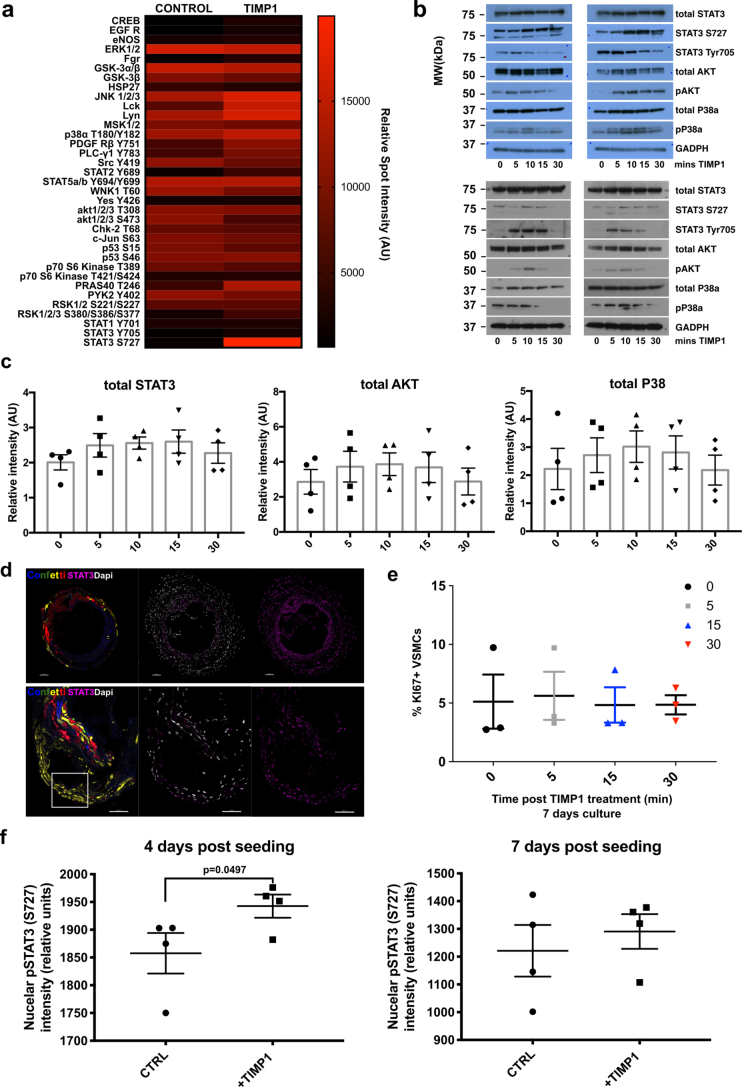
TIMP1 induces phosphorylation of STAT3 in VSMCs. **a**, Heatmap showing relative spot intensity of phosphokinase array analysis of hVSMCs following 15 minutes 500 ng/mL rhTIMP1 or vehicle control treatment on a black (low) to red (high) scale [AU]. N = 1. **b**, Western blots of hVSMCs from 4 different donors showing total, pS727 and pTyr705 STAT3, total and phospho AKT, total and phospho-p38 (p-p38), and GAPDH in serum-starved cells after 0, 5 10, 15 or 30 minutes rhTIMP1 treatment. **c**, Quantification of relative band intensity in (**b**) for total STAT3, total AKT and total p38 protein levels, normalized to GAPDH. Points show independent hVSMC isolates (N = 4 donors), bars mean, error bars SEM. **d**, pSTAT3 S727 immunostaining (magenta) in cryosections from injured carotid artery of Myh11-Confetti (top, N = 3 animals) or atherosclerotic plaque from Myh11-Confetti/Apoe animals fed a HFD (lower panel, N = 3). Signals for nuclear DAPI (white) and confetti lineage-label are also shown. White box indicate region shown in Fig. 6g. Scale bar= 50 μm. **e**, %KI67+ hVSMCs in samples treated with 500 ng/mL rhTIMP1 following serum-starvation for indicated timepoint. Symbols show average values for independent hVSMC isolates (N = 3, analyzed in triplicate), lines the mean, error bars SEM. **f**, Nuclear pSTAT3 (S727) intensity in FACS-isolated EYFP+ mVSMCs without (CTRL, dots) or with 500 ng/mL rmTIMP1 (squares) either 4 (left) or 7 days post seeding (right). Symbols show average values from independent (N = 4 Myh11-EYFP animals analyzed in quadruplicate), lines mean, error bars SEM, p-value: two-tailed *t*-test. Source data

The signaling pathways activated by TIMP1 show context dependency^34,36,37^, so we screened for TIMP1-mediated phosphokinase activation in hVSMCs. This suggested that signal transducer and activator of transcription (STAT3) phosphorylation at serine 727 (S727) is strongly increased in TIMP1-treated cells, which was confirmed by western blotting in independent hVSMC isolates (Fig. 7a,b and Extended Data Fig. 7a). Increased phosphorylation was observed at both S727 and Tyr705, while total STAT3 protein levels were unchanged (Fig. 7b,c and Extended Data Fig. 7b,c). The rapid and transient induction of STAT3 phosphorylation suggests a direct response to TIMP1 (Fig. 7b,c). We also analyzed the effect of TIMP1 on signal transducers known to regulate VSMC growth. Similar to STAT3, phosphorylation of AKT (Ak strain transforming) was induced by TIMP1, whereas phosphorylation levels of p38 mitogen-activated protein kinase (p38) were variable between hVSMCs from different individuals (Fig. 7b,c and Extended Data Fig. 7b,c).

The role of these signaling pathways in TIMP1-mediated stimulation of clonal VSMC proliferation was investigated by addition of small-molecule inhibitors in the clonal VSMC proliferation assay. Inhibition of STAT3 using TT-101 abolished the increased clone number in response to TIMP1, whereas the impact of AKT and p38 inhibition was less obvious (Fig. 7d). To further test the specificity, we depleted STAT3 using siRNA, which also prevented the TIMP1-mediated increase in clone formation (Fig. 7e). Phosphorylation of STAT3 leads to dimerization, nuclear import and binding at target gene promoters^38^. Consistent with its activation, STAT3 chromatin immunoprecipitation showed increased binding in TIMP1-treated cells, specifically at promoters of canonical STAT3 target genes (JUNB, TWIST1), compared with control cells (Fig. 7f).

VSMC-derived cells in atherosclerotic plaques and neointimal lesions expressed high levels of pSTAT3 compared with medial cells (Fig. 7g and Extended Data Fig. 7d), indicating its involvement in clonal VSMC expansion. To further assess whether STAT3 phosphorylation is linked with VSMC proliferation, we co-stained hVSMCs for KI67 and pSTAT3 (S727) after TIMP1 treatment. Overall, KI67^+^ cells had higher nuclear pSTAT3 levels compared with KI67^−^ cells and there was significantly increased pSTAT3 expression 15 min after TIMP1 stimulation (Fig. 7h,i). There was no effect of TIMP1 on hVSMC proliferation within this time frame (Extended Data Fig. 7e). We next analyzed VSMCs isolated from aortas of lineage-labeled mice and cultured to mimic phenotypic switching and activation of proliferation. STAT3 phosphorylation was also induced by TIMP1 after 4 days of culture, but this effect was less pronounced in cells analyzed at day 7 (Extended Data Fig. 7f). Interestingly, baseline pSTAT3 intensity was higher in cells cultured for 4 days after isolation, compared with day 7 cells, suggesting the existence of a ‘window of opportunity’ with higher responsiveness to TIMP1. Overall, these data link TIMP1-mediated STAT3 phosphorylation to the activation of VSMC proliferation.

## TIMP1 signals through CD74 to induce VSMC proliferation

**Fig. 8:**
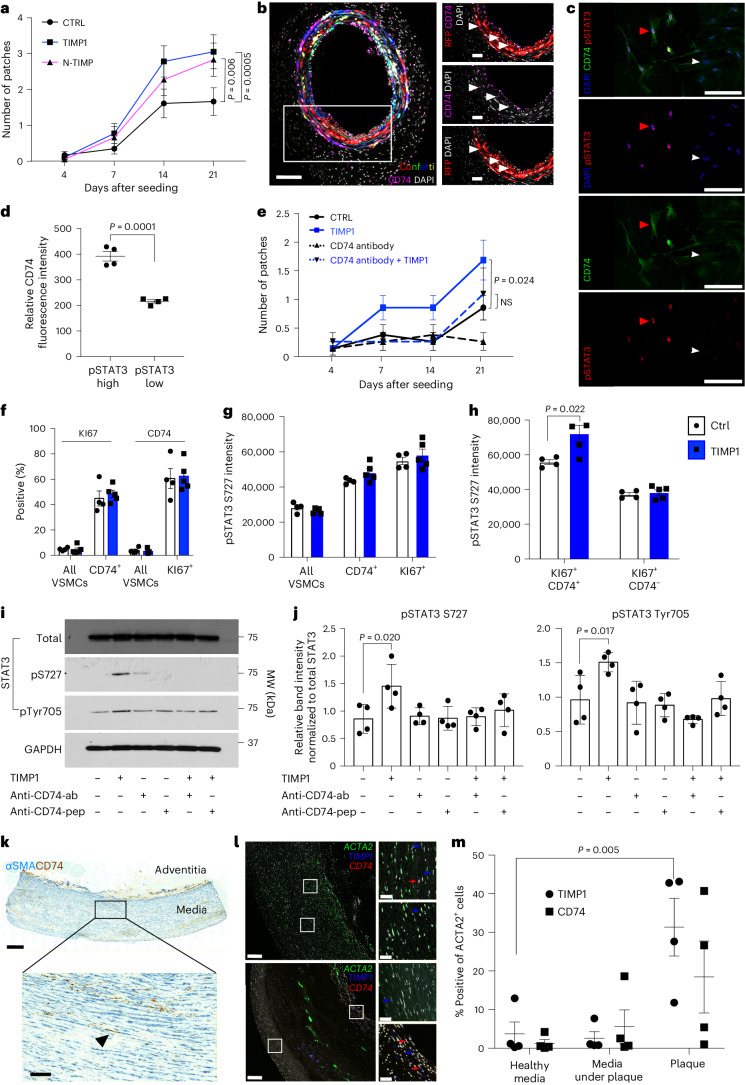
TIMP1 signals to STAT3 via CD74 in a disease-relevant mechanism. **a**, Number of clonal patches formed by lineage-labeled VSMCs, treated with 500 ng ml^−1^ recombinant TIMP1 or equimolar N-TIMP1 over 21 days of culturing. The points indicate means (*N* = 4 Myh11–Confetti animals analyzed in triplicate), and the error bars indicate s.e.m.; *P* value: generalized linear model. **b**, CD74 immunostaining (magenta), Myh11–Confetti signal and DAPI nuclear staining (white) in carotid arteries 10 days after ligation. The magnified view of the boxed region shows only the RFP Confetti reporter. White pointers mark CD74/RFP double-positive cells. *N* = 5 Myh11–Confetti animals. Scale bars = 100 µm (overview), 30 µm (zoom). **c**, Immunostaining for pSTAT3 S727 (red) and CD74 (green) with nuclear DAPI (blue) in mVSMCs treated with 500 ng ml^−1^ rmTIMP1 4 days after isolation. The arrowheads mark STAT3-high (red) and STAT3-low cells (white). Scale bars = 50 µm. **d**, Quantification of cellular CD74 levels in **c**, stratified by nuclear pSTAT3 S727 intensity. The dots show the average per animal (*N* = 4 animals analyzed in quadruplicate), the lines indicate means and the error bars indicate s.e.m.; *P* value: two-tailed *t*-test. **e**, Number of clonal patches formed by lineage-labeled VSMCs ±500 ng ml^−1^ rmTIMP1 and/or CD74-blocking antibody or peptide. The points indicate means (*N* = 4 Myh11–Confetti animals analyzed in triplicate), and the error bars indicated s.e.m.; *P* value: generalized linear model (CTRL as base variable). Independent generalized linear modeling (TIMP1 as base variable) showed a significant difference between TIMP1 and CD74-antibody^+^ TIMP1 (*P* = 0.049). **f**–**h**, Quantification of imaging flow cytometry of lineage-labeled aortic VSMCs from 13-week-old, HDF-fed (4 weeks) Myh11–EYFP/Apoe animals treated with rmTIMP1 or vehicle control. The symbols show values for individual animals (*N* = 4 (ctrl), 5 (TIMP1)), the bars indicate means and the error bars indicate s.e.m.; *P* value: two-tailed *t*-test. **f**, Percentages of all VSMCs, or indicated subpopulations, expressing KI67 and CD74. **g**–**h**, pSTAT3 S727 median fluorescence intensity. **i**,**j**, Representative western blot (**i**) and quantification (**j**) of serum-starved control and rhTIMP1-treated hVSMCs (5 min) ± pretreatment with CD74-blocking antibody (CD74-ab) or peptide (CD74-pep). The points show independent hVSMC isolates (*N* = 4 donors), the lines indicate means and the error bars indicate s.e.m.; *P* value: one-way ANOVA. **k**, CD74 (brown) and αSMA (blue) immunohistochemistry in non-plaque human aorta. Scale bars = 500 µm (overview), 100 µm (zoom). *N* = 7 donors. **l**, RNA in situ hybridization for *ACTA2* (green), *TIMP1* (blue) and *CD74* (red) in a human healthy aorta and a plaque-containing carotid artery. The arrows indicate *TIMP1*/*ACTA2*^+^ (red) and *CD74*/*ACTA2*^+^ cells (blue). Scale bars = 250 µm (overview), 50 µm (zoom). **m**, Quantification of *TIMP1* or *CD74* expression in *ACTA2*^+^ cells in non-plaque aortas (Healthy media) or carotid endarterectomy regions (Media under plaque, Plaque). The symbols indicate different donors (*N* = 4 donors per condition), the lines indicate means and the error bars indicate s.e.m.; *P* value: one-way ANOVA. Source data

Signaling by TIMP1 can be executed via several surface receptors that bind to different TIMP1 protein domains^37^. We found that the ability to induce clonal VSMC proliferation was retained by the N-terminal part of TIMP1 (Fig. 8a), which binds the HLA class II histocompatibility antigen gamma chain, CD74 (ref. ^39^), that has also been detected in modulated or ‘macrophage-like’ VSMCs in experimental atherosclerosis^40^.

**Extended Data Fig. 8:**
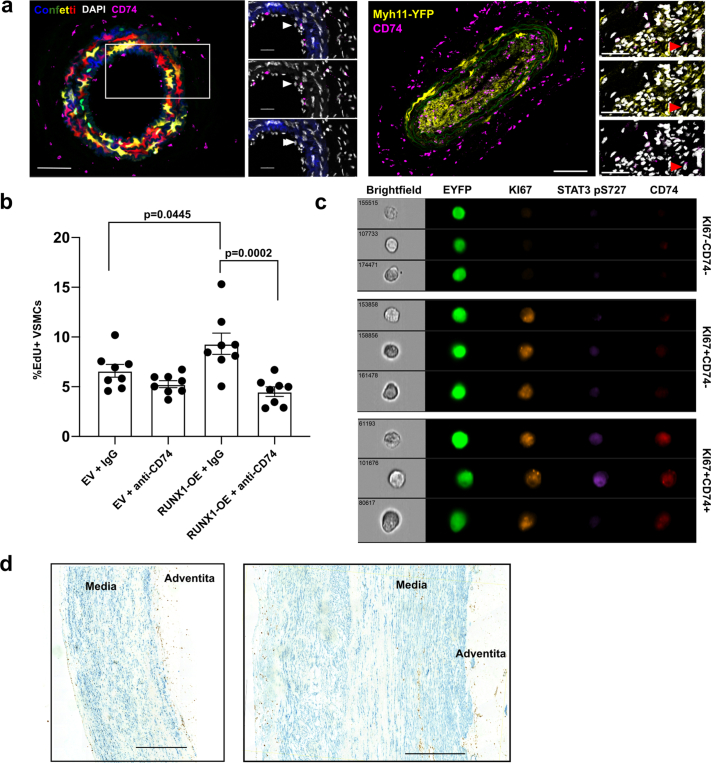
CD74 expression and function in VSMCs. **a**, Example images of immunostaining for CD74 (magenta) in cryosections of Myh11-Confetti lineage-labeled carotid arteries, analyzed 28 days (left) or 10 days post carotid ligation (right). Arrows mark CD74+ lineage labeled VSMCs. Scale bars: 100 µm (overview), 30 µm (zoomed). N = 5 animals. **b**, Bar graph showing the percentage of EdU+ lineage-labeled VSMCs in samples infected with lentivirus carrying an empty vector (EV) or overexpressing RUNX1 (OE) in the presence of a control (IgG) or a CD74 blocking antibody (CD74). VSMC-lineage labeled medial cells were isolated from mouse aorta. Points show values for different animals (N = 8), bars the mean, error bars SEM, p-value: one-way ANOVA**. c**, Example imagestream analysis of lineage-labeled EYFP+ (green) VSMCs from Myh11-EYFP/Apoe animals stained for KI67 (orange), pSTAT3 S727 (magenta) and CD74 (red). Left columns show brightfield images. **d**, Example images of CD74 (brown) and αSMA (blue) immunostaining in non-plaque human aorta. Scale bar=500 µm. N = 7 tissue samples from different donors. Source data

Immunostaining revealed that Myh11-lineage-labeled VSMCs also express CD74 protein after vascular injury in vivo (Fig. 8b and Extended Data Fig. 8a), providing evidence that TIMP1 could function via CD74 in VSMCs, as suggested^34^. CD74 was also detected in cultured lineage labeled mouse VSMCs (mVSMCs) (Fig. 8c); we found an association between high pSTAT3 and greater CD74 expression (Fig. 8c,d), indicating that CD74 signaling results in phosphorylation of STAT3. To directly test whether TIMP1 acts via CD74 in VSMCs, we used a CD74-blocking antibody, which abolished TIMP1-augmented VSMC clone formation (Fig. 8e). Interestingly, anti-CD74 also reduced the frequency of clone formation in untreated cells, which could be due to blocking the effect of TIMP1 secreted by VSMCs after culture-induced phenotypic switching, or other CD74 ligands such as MIF. Blocking CD74 in VSMCs overexpressing RUNX1 also abolished the stimulation of proliferation (Extended Data Fig. 8b), suggesting that the observed increase in *Timp1* expression could be important for the RUNX1-OE phenotype.

To assess whether the TIMP1–CD74–pSTAT3 axis is active in atherosclerosis, we investigated lineage-labeled *Apoe*^−/−^ animals after a 4-week exposure to a high-fat diet, at a timepoint before robust VSMC contribution in lesions^10,20^. Imaging flow cytometry showed a low frequency of KI67^+^ lineage-labeled VSMCs, as expected, and this varied between animals. We found a striking enrichment of CD74 expression in KI67^+^ cells (Fig. 8f), and the levels of phospho-STAT3 were higher in proliferating (KI67^+^) and in CD74^+^ VSMCs, compared with all lineage-labeled cells (Fig. 8g). This suggests that CD74 and phosphorylation of STAT3 are linked to VSMC proliferation. TIMP1 treatment further increased pSTAT3 levels in CD74^+^, but not in CD74^−^, proliferating VSMCs (Fig. 8h and Extended Data Fig. 8c), providing in vivo evidence linking this signaling pathway to the initiation of VSMC proliferation in atherosclerosis.

Next, we explored the TIMP1–CD74–pSTAT3 axis in humans. As shown in Fig. 8i, TIMP1-induced STAT3 phosphorylation was reduced in cells co-treated with the anti-CD74 antibody and a similar effect was observed using a CD74-blocking peptide^41^, showing that TIMP1-mediated induction of STAT3 phosphorylation is downstream of CD74 (Fig. 8i,j). We found that CD74 was detected by immunostaining in αSMA^+^ cells in non-plaque arteries from human organ donors and in samples taken after carotid endarterectomies (Fig. 8k and Extended Data Fig. 8d). To quantify expression, we performed multi-probe in situ hybridization (Fig. 8l,m). *TIMP1* and *CD74* transcripts were detected in *ACTA2*^+^ VSMCs in non-plaque arteries, but were observed at low frequency. Compared with medial cells in arteries without lesions, the frequency of CD74 and TIMP1 expressing cells was elevated in samples with obvious plaques. Quantification of positive cells revealed substantial co-expression of TIMP1 and CD74 with ACTA2 (12–43% of ACTA2^+^ plaque cells expressed TIMP1; 1–41% for CD74). Altogether, these findings suggest that the induced expression of TIMP1 upon VSMC activation could stimulate VSMC proliferation by binding to CD74 via activation of STAT3 phosphorylation.

## Discussion

We here used GRN modeling after acute vascular injury to identify factors governing the activation of VSMC proliferation, which could be important to reduce vascular disease susceptibility and for disease prevention strategies. Our analysis indicates that differential use of shared transcription factors plays an important role in this process. This also suggests that VSMC state-dependent mechanisms may underlie observed context-specific functions, such as the dichotomous function of monocyte chemoattractant protein-1 (MCP-1), encoded by *Ccl2* (ref. ^22^). Using in silico simulation experiments, we prioritize both factors known to control VSMCs in disease and candidate regulators proposed to safeguard the contractile state (for example, THRA) or promote cell activation (for example, RUNX1, CEBP/D, ENO1). We experimentally validate RUNX1 and the TIMP–STAT3–CD74 axis in vivo and in vitro as VSMC regulators with relevance to human diseases. Genetic evidence linking these factors to vascular disease includes a variant in the RUNX1 locus associated with stroke (rs116262092-A) and colocalization of a STAT3 VSMC eQTL with a CAD GWAS signal (Supplementary Table 1g). Furthermore, vascular abnormalities and increased aneurysm risk have been identified in patients with missense STAT3 variants causally implicating this pathway in vascular regulation^42,43^.

RUNX1 has well-known functions in hematopoiesis and also impacts differentiation and proliferation in other contexts, including cardiovascular cells^44,45^. We here show that RUNX1 increases the frequency of VSMCs that start proliferating, possibly by directly inducing the expression of VSMC ‘transition state’ genes, such as *MMP14*. It is therefore tempting to speculate that this pioneer transcription factor could drive the activation of alternative transcriptional programs in VSMCs. The expression of RUNX1 protein in human arteries with intimal thickening is higher compared with those with a healthy morphology, suggesting that RUNX1 may indeed play a role in vascular changes predisposing to disease.

We show that TIMP1, a target of transcriptional rewiring, promotes the establishment of clonal VSMC proliferation itself, suggesting a positive feedback loop that might impact vascular disease susceptibility. TIMP1 also stimulates proliferation in hVSMC cultures^34^, and higher TIMP1 serum levels have been associated with elevated cardiovascular disease risk in humans^31^. Future studies are needed to assess whether increased TIMP1 in serum is a cause or consequence of activated VSMCs within diseased arteries. In experimental atherosclerosis, *Apoe*^−/−^, *Timp1*^−/−^ animals had smaller plaques with reduced VSMC content compared with *Apoe*^−*/*−^ controls^30,46^. By contrast, femoral artery injury yielded larger neointimal lesions and increased MMP activity in *Timp1*^−/−^ animals^47^. As VSMC lineage tracing was not performed, the impact on VSMC proliferation was not assessed in these studies. Interpreting the effects of global TIMP1 manipulation on VSMC function is further complicated by, firstly, the dual effect of TIMP1 on MMP activity and cell signaling and, secondly, the ability of TIMP1 signaling to impact multiple cell types in vascular disease^34^. Whereas TIMP1 depends on AKT in macrophages^34^, CD74 signaling via STAT3 is required for TIMP1 stimulation of VSMC clonal proliferation in vitro, and this axis is also active in vivo.

The regulatory interactions observed here are consistent with gene expression changes in atherosclerosis and include factors impacting on cultured VSMCs and VSMC-derived plaque cells. For example, enriched motifs in single cell ATAC-seq analysis of human CAD patients include AP-1, RUNX and CEBP, and also highlighted the importance of STAT3 in human vascular disease^2^. Similarly, AP-1 and STAT motifs were highlighted by analysis of lineage-traced VSMCs that adopt a more fibroblastic phenotype during mouse atherogenesis^1^. We also detected RUNX1 and CD74 within αSMA^+^ cells in developed human atherosclerotic lesions. However, whether enrichment of these motifs in atherosclerosis is caused by ongoing VSMC activation in disease or implies that these factors also function at later disease states remains to be tested. Interestingly, studies in zebrafish heart regeneration showed that RUNX1 promotes expression of smooth muscle cell genes in mesenchymal cells^45^, suggesting that in addition to regulating VSMC proliferation, it could also impact fibrous cap formation. The potential links between mechanisms regulating VSMC proliferation and VSMC-derived atherosclerotic plaque cell phenotypes are an exciting topic for future research.

## Methods

Detailed methods are available in Supplementary Information; antibodies are listed in Supplementary Table 4 and primer sequences in Supplementary Table 5.

### Animals and procedures

All experiments were done according to UK Home Office regulations (project licenses P452C9545 and PP7513347) and were approved by the Cambridge Animal Welfare and Ethical Review Body. Mice (C57Bl/6) were housed on a 12 h dark and light cycle at 19–21 °C and 45–65% humidity. Alleles were previously generated; Myh11–CreERt2 (RRID:IMSR_JAX:019079) is a Y-linked transgene that confers expression of a tamoxifen-inducible Cre recombinase in smooth muscle cells, Rosa26–Confetti (RRID:IMSR_JAX:013731) and Rosa26–EYFP (RRID:IMSR_JAX:006148) are Cre-recombination reporter alleles, KI67–RFP (RRID:IMSR_JAX:029802) is an insertion in the Mki67 locus resulting in expression of a KI67–RFP fusion protein, and the mutant Apoe allele (RRID:IMSR_JAX:002052) sensitizes mice to atherosclerosis induced by a high-fat diet (HFD). VSMC lineage labeling was achieved by administration of tamoxifen (10 intraperitoneal injections of 1 mg ml^−1^ over 2 weeks). Note that Confetti–GFP induction occurs at low frequency^4^. Myh11–CreERt2 is Y linked, so males were used for VSMC lineage tracing. The animals were allowed to rest for >1 week after the last injection to allow tamoxifen washout before tissue harvest, HFD feeding (21% fat and 0.2% cholesterol, Special Diets Services) and vascular injury, or randomized into groups receiving intraperitoneal injections of recombinant mouse TIMP1 protein (200 μg kg^−1^, *N* = 5) or vehicle (phosphate-buffered saline (PBS), *N* = 4) daily for 9 days at the end of a 4 week HFD protocol. Pre-operative analgesic was given subcutaneously (~0.1 mg per kg body weight, buprenorphine) and the left carotid artery was tied off with a silk suture just under the bifurcation point under isoflurane anesthesia (inhalation, 2.5–3%; 1.5 l min^−1^ induction, maintained at 1.5%). The animals were euthanized (cervical dislocation or CO_2_ asphyxiation) 5–28 days after surgery and perfused with cold PBS before tissue removal.

### Human tissue

Human arteries were collected after informed consent and approval by the Cambridgeshire 1 or East of England—Cambridge South Research Ethics Committee (REC ref 15/EE/1052, H00/514) were obtained. Non-plaque aortas were obtained from organ donors via the Cambridge Collaborative Biorepository for Translational Medicine. Carotid endartectomy samples were obtained from the Royal Papworth Hospital research tissue bank. Cell isolates from a total of 13 donors (age (years) and sex: 27, male; 20, male; 70, male; 75, female; 45, female; 65, male; 62, female; 51, male; 68, female; 69, male; 72, female; 68, male; 61, male) and histological analysis of 9 carotid artery samples (72, female; 82, male; 80, female; 67, male; 67, female; 58, female; 74, male; 54, male; and 50, female) and 10 aortas (60, male; 24, male; 84, female; 65, male; 49, male; 35, male; 41, male; 75, female; 18, male; 59, female) were included. Both male and female tissue and cell isolates were included in all analyses. The experiments with human samples conformed to the principles outlined in the Declaration of Helsinki.

### VSMC isolation, culture and treatment

The hVSMCs were cultured from the aortas of patients undergoing cardiac transplantation or aortic valve replacement in an hVSMC-specific medium (Promocell, SMC-GM2, C22062) supplemented with antibiotics. The hVSMCs were serum starved in media supplemented with 0.1% BSA before TIMP1 treatment. Single-cell suspensions of mVSMCs were generated from freshly isolated aortas of wild-type or VSMC lineage-labeled animals (Myh11–Confetti or Myh11–EYFP) and cultured in DMEM supplemented with 10% FCS and antibiotics (complete medium).

The RUNX1 overexpression lentiviral vector was generated by inserting full-length mCherry and Runx1 cDNA (*Runx1–202*) linked by T2A into a pLentiGFP backbone (Addgene, catalog number 17448). VSMCs were transduced with lentivirus in media containing 10 µg ml^−1^ protamine sulfate (Sigma, P3369). Transfection with siRNA (50 nM ON-TARGETplus SMART Pool, Dharmacon, targeting human *RUNX1* (L-003926-00-0005), mouse *Runx1* (L-048982-00-0005), mouse *Stat3* (L-040794-01-0005) or non-targeting control siRNA, Control Pool, D-001810-10-05) was done using Lipofectamine RNAiMAX transfection reagent (13778030, Invitrogen).

Cells were treated with recombinant (r) mouse (m) or human (h) protein, peptide or small-molecule inhibitors as detailed in the figure legends and Supplementary Table 3. Transfection with siRNA was performed the day before TIMP1 addition, and cells were pre-treated for 1 h (CD74 antibody, GM6001, STAT3i, AKTi, p38i1, p38i2, p38 control i) or 6 h (CD74 peptide) before TIMP1 treatment.

### Clonal VSMC proliferation assay

Cells (EYFP^+^ VSMCs from aortas of Myh11–EYFP isolated by flow-cytometry-assisted cell sorting or medial cells from Myh11–Confetti animals mixed with medial cells from wild-type animals in a 1:3 ratio) were seeded in 96-well imaging plates (5,000 cells per well, CellCarrier-96 Ultra, Perkin Elmer) in complete medium. Low-titer lentiviral transduction with RUNX1 and control virus was performed 3 days after seeding, or cells were treated as indicated in complete medium from day 4 after seeding. Medium with fresh reagents was added twice weekly. Cells were imaged 4, 7, 14 and 21 days after plating using an Opera Phenix high-content screening system (Perkin Elmer). Image analysis was done using Harmony software v5 (Perkin Elmer), and quantification was performed in Fiji v2.15.1. Patches were defined as three or more contiguous EYFP^+^RFP^+^ (RUNX1 experiment) or same-color lineage-traced Confetti^+^ cells (TIMP1 assays).

### ATAC-seq

Single-cell suspensions of carotid arteries from 4 to 5 lineage-labeled Myh11–EYFP/Mki67–RFP animals (11 weeks old) were generated 7 days after carotid ligation surgery, stained for SCA1 and EYFP^+^SCA1^+^ or EYFP^+^SCA1^−^ cells isolated by cell sorting (BD FACSAria III, BD Bioscience). In parallel, lineage-traced (EYFP^+^) VSMCs were isolated from non-ligated carotid arteries of a non-ligated littermate. The Omni-ATAC protocol^48^ was used to process 5,000 cells from each sample type in parallel (two independent replicates generated on separate days), using 10–13 PCR amplification cycles and libraries sequenced with a 50 bp paired-end run cycle (Illumina HiSeq or HiSeq2500-RapidRun, 40–60 million reads per sample).

The details of ATAC-seq data analysis are provided in Supplementary Information. Condition-specific peak lists include peaks overlapping at least 50% between biological replicates, and the pan-VSMC peak list is the union of condition-specific lists. Peaks were associated with genomic features using ChIPseeker v.1.24.0. Differential accessibility was scored in LIMMA (SeqMonk v.1.47.2) using a >2-fold change threshold and a Benjamini–Hochberg adjusted *P* < 0.01. Peaks were annotated to genes and GO term enrichment analysis performed using GREAT v.4.0.4 (ref. ^49^; http://great.stanford.edu/public/html/index.php) with default settings and all genes as a background. For the SCA1^+^ condition, the top 4,000 peaks ranked by fold change were used. Motif enrichment analysis was done on 500 bp genomic sequences centered on ATAC-seq peak summits using MEME-ChIP v.5.4.1 (ref. ^50^) for differentially accessible peaks using HOCOMOCO mouse v.11.

### GRN analysis

The scRNA-seq profiles of VSMCs isolated from mouse carotid arteries 5 days after carotid ligation surgery (GSE162167) and the bulk ATAC-seq data generated here (accession number below) were used for GRN modeling using CellOracle v.0.10.5 (ref. ^24^). A ‘BaseGRN’, including a list of all potential transcription factor-target gene interactions, was constructed using pan-VSMC ATAC-seq peaks. Transcriptional start site (TSS) annotation and motif scan were performed with this union peak data using the default settings of CellOracle. Cells from the day 5 carotid ligation injury scRNA-seq data, clustered as described^20^, were annotated with VSMC states (cluster 9 as CYC; clusters 4 and 3 as PrP; cluster 2 as LNK; clusters 0, 5, 7 and 8 as non-RSP; cluster 1 as stress; and clusters 6 and 10 as path 2 (Fig. 2a and Extended Data Fig. 2a)) and processed using Scanpy v.1.9.1 (ref. ^51^). For GRN modeling, the top 4,000 highly variable genes were supplemented with transcription factors showing pseudotime-dependent expression (*Eno1*, *Id3*, *Mef2c*, *Hif1a*, *Nfia*, *Hmga1*, *Ebf1*, *Rora*, *Klf9*, *Jund*, *Foxp1*, *Thra*, *Prrx1*, *Nfix*), and *Stat3*, *Twist1* and *Runx2*.

GRNs were constructed with the top 2,000 interactions for all VSMC states. Topological analyses of GRNs were performed with CellOracle, and GRNs were visualized with Cytoscape v.3.7.2 (ref. ^52^). GRNs for the stress and path 2 states (colored gray in Fig. 2a) were not investigated further. Venn and Euler diagrams were created using CRAN R package eulerr v.7.0.0. GO term analysis and visualization were performed with BiNGO v.3.0.4 and GOlorize v.1.0.0.beta1 with the default settings, hypergeometric test, Benjamini–Hochberg false discovery rate correction and a significance level of 0.05. Communities were detected using clusterMaker2 v.1.3.1 (ref. ^53^) with the MCODE algorithm^54^ (fluff option on).

A rewiring score was calculated for each node as the sum of absolute change in connectivity score between the PrP and non-RSP networks in R v.4.2.2 using igraph v.1.4.2 (https://igraph.org) functions to obtain adjacency matrices. Differential gene expression values for cells in PrP versus non-RSP cell states were calculated using the Wilcoxon rank-sum test (with Bonferroni correction as the method for multiple testing correction), and gene expression values were plotted using Seurat v.4.3.0 (ref. ^55^) based on sctransform-normalized values^20^.

In silico simulations were performed with the default settings and top 10,000 interactions in all GRNs with an expression value of zero for the knockout simulations or of 1.5 times the maximum gene expression value for the overexpression simulations. Systematic knockout and overexpression simulations were performed for all TFs present in the GRNs with the same parameters along the proliferation-associated trajectory including cells from the non-RSP, LNK, PrP and CYC populations. TFs were scored based on the sum of positive or negative PSs, which were calculated as the inner product of developmental flow and simulation vectors as described^24^. Root cell selection for pseudotime calculation was based on *Myh11* expression and cell clustering.

Before GRN modeling with the post-carotid ligation day 7 dataset, these data were integrated with the day 5 dataset with sctransform-based normalization using Seurat v.4.3.0 (ref. ^55^) and further clustered (24 principal components and 1.9 resolution) to identify cell clusters corresponding to equivalent cell states. This information was then used for the GRN modeling.

### Analysis of published scRNA-seq datasets

Filtered scRNA-seq profiles (GSE155513) of VSMC-lineage labeled cells from mouse atherosclerotic arteries from 3 time points (8, 16 and 22 weeks) were integrated with sctransform-based normalization (Seurat v.4.3.0)^55^ and clustered (20 principal components at 0.3 resolution). Differential expression testing for each cell cluster versus all other VSMCs, or for modulated (clusters 3 and 0) versus contractile (cluster 1) cells, was done with the Wilcoxon rank-sum test (adjusted *P*-value (*P*_adj_ ) < 0.05, Bonferroni corrected).

Gene set signatures were assessed using UCell v.2.2.0 (ref. ^56^). PrP-state signature genes include those showing differential expression between PrP and non-RSP cells (log_2_ fold change (FC) > 0.5, *P*_adj_ < 0.05; Supplementary Table 1e). ‘Positive regulator’ signatures were defined as TFs with the top 10 highest positive PSs for overexpression simulation or negative PSs for knockout simulation plus the target genes of these TFs in the PrP GRN (edge connectivity > 0.1). ‘Negative regulator’ signatures contained TFs with the top 10 highest negative PSs for overexpression simulation or positive PSs for knockout simulation plus the target genes of these TFs in the PrP GRN (edge connectivity > 0.1).

The scRNA-seq profiles (GSE155512) of human atherosclerotic carotid plaques^13^ were filtered to include only cells with a minimum of 200 and a maximum of 4,000 genes, total counts >500 and mitochondrial reads <10%, and data from three different patients integrated. Data integration and clustering were repeated after subsetting VSMCs (17 principal components; Seurat v.4.3.0)^55^. Differential expression testing was performed for each cell cluster against all other VSMCs as above, with log_2_ fold change (FC) > 0.25 threshold. Heat maps were created with the CRAN R package pheatmap v.1.0.12 with complete linkage and correlation as the distance metric.

### Colocalization analysis

Colocalization analyses were done for human orthologs of genes ranking in the top 50 for the rewiring score or top 10 for a positive or a negative PS, plus factors associated with TIMP1 signaling (STAT3, CD74). Summary statistics for CAD GWAS^57^ were converted from hg19 to hg38 build with CrossMap^58^ and colocalized with VSMC *cis*-eQTL from smooth muscle cells isolated from human umbilical cord (*N* = 1,499)^59^ and human aortic smooth muscle cell *cis*-eQTL for quiescent (*N* = 139) and proliferative (*N* = 145) cells^60^. For eCAVIAR, variants with *P* < 0.001 within 1 mb of the top *cis*-eQTL association for each gene were overlapped with corresponding GWAS variants (*P* < 0.001) in the same locus. An eCAVIAR colocalization test was performed for genes that had five or more overlapping variants with GWAS summary data. Linkage between overlapping variants was estimated from individuals of European ancestry in the 1000 Genomes Phase 3 using PLINK^61,62^. Analysis based on Summary-based Mendelian Randomization (SMR) was done with SMR v1.3.1 (ref. ^63^) with default settings and peqtl-smr set to 0.001. European samples in the 1000 Genomes project served as reference for linkage disequilibrium estimation. Colocalization events with eCAVIAR colocalization posterior probability (CLPP) > 0.01 or *P*_SMR_ < 0.05 was considered significant.

### Bulk gene expression

RNA was extracted using an RNeasy Mini kit (Qiagen, 74104), and cDNA was generated using QuantiTect Reverse Transcriptase (Qiagen, 205311). Bulk RNA-seq was conducted on RNA isolated from of six different hVSMC isolates following 3 h of serum starvation in serum-free media containing 0.1% BSA, followed by 6 h of TIMP1 (or vehicle) treatment in serum-free media containing 0.1% BSA. Libraries were prepared from oligo-dT-purified mRNA and sequenced (150 bp paired-end reads, Illumina Novaseq 6000). Raw data reads were trimmed with Trim Galore v.0.6.7 and aligned to the human genome (GRCh38) with Kallisto v.0.46.2. Gene set enrichment analysis^64^ was performed after trimmed mean of *M* values (TMM) normalization. Quantitative real-time PCR was performed using SsoAdvanced Universal SYBR Green Supermix (Biorad, 1725270).

### Protein detection

Whole-cell protein lysates were prepared in RIPA buffer freshly supplemented with proteinase inhibitors (Millipore) and phosphatase inhibitors (Millipore) and separated on gradient (4–12%) polyacrylamide gels. Protein concentration was determined using the BCA method (23227, Pierce BCA protein assay kit, Thermo Fisher). Primary antibodies were detected by HRP-labeled secondary antibodies using chemiluminescence detection (Amersham ECL detection reagent, GE Healthcare) and exposure to photographic film. Phosphokinase array (R&D Systems, ARY003C) was incubated with cell lysates according to the manufacturer’s instructions. Densitometry values of spots were calculated in ImageJ and expressed as arbitrary units.

### Immunostaining, EdU incorporation and imaging

Cryosections (14 μm) of ligated left carotid arteries from lineage-labeled Myh11–Confetti animals and plaque-containing arteries from Myh11–Confetti/Apoe animals after high-fat feeding were permeabilized, stained and mounted in RapiClear 1.52 (Sunjin Lab). Confocal imaging was done with a Leica SP8 scanning laser microscope (Leica) using a ×20 lens, and image analysis was done in Imaris v9.2.

Human arteries were formaldehyde fixed and paraffin embedded, and sections (4 μm) were dewaxed and processed for antigen retrieval; sequential sections were either H&E stained, or co-stained for αSMA and either RUNX1, TIMP1 or CD74.

Cells cultured in 96-well plates and treated as indicated in figure legends were fixed and stained. Cells seeded in 96-well imaging plates (10,000 per well) were incubated with EdU (10 µM) for 16 h, and EdU incorporation was detected using the Click-iT EdU kit (C10340, Thermo Fisher Scientific), stained with DAPI for 10 min. Cells were imaged using an Opera Phenix high-content screening system, with a ×20 water objective (Perkin Elmer). Image analysis was done using Harmony software (Perkin Elmer), quantification based on intensity values with nuclei defined by DAPI staining, and for mouse VSMCs from Myh11–EYFP animals, within cell borders (EYFP). Quantification of EdU^+^ cells was done by thresholding intensity values measured in detected nuclei.

### Chromatin immunoprecipitation

Chromatin immunoprecipitation (ChIP) was performed on 3 × 10^6^ hVSMCs using a SimpleChIP kit according to the manufacturer’s instructions (Cell Signaling Technology, 56383). Cells were cross-linked with formaldehyde (1% final concentration, 15 min). Chromatin (5 μg) sheared using a Diagenode bioruptor (15 pulses of 30 s with a 30 s rest) was immunoprecipitated with 10 μl STAT3 antibody (Clone D3Z2G, Cell Signaling Technology, 12640) or an equal amount of control IgG (Cell Signaling Technology, 2729). Antibody-bound protein–DNA complexes were captured using protein-G-coated magnetic beads, reversed cross-linked and digested with proteinase K. DNA was purified and quantified using quantitative (q) PCR.

### Flow cytometry

Single-cell suspensions were incubated with 5 µg ml^−1^ TruStain FcX anti-mouse CD16/32 antibody (Biolegend) in FACS buffer (0.5% (w/v) BSA in PBS) for 15 min on ice, stained with primary antibody for 30 min at room temperature and washed twice (FACS buffer). Samples were then incubated with secondary antibody (in FACS buffer, 30 min at room temperature) and washed twice (FACS buffer), for non-conjugated antibodies. Intracellular targets were stained using the Foxp3 staining buffer set (eBioscience). Cells were analyzed using an Imagestream system (Amnis ImageStream XMk II, Luminex) using IDEAS v6.2 or sorted (BD FACSAria III, BD Bioscience). Gating was done using cells stained with control IgG or non-expressing cell populations.

### RNA scope

RNA in situ hybridization was performed using RNA Scope Multiplex Fluorescent v2 kits, according to the manufacturer’s instructions (ACD). Experiments used Hs-TIMP1-C2 coupled to opal 570, Hs-CD74-C3 coupled to opal 620 and Hs-ACTA2-C1 coupled to opal 690 (ACD). Imaging was done using a ZEISS Axioscan slide scanner. Analysis was performed in QuPath using the subcellular detection feature.

### Statistical analysis

Group size and replicate numbers were decided on following power calculations based on observed or expected variation using a power of 90% and a 0.05% type I error rate. Where technical repeats were conducted of the same cell line or animal, these have been averaged and biological repeats used for statistical analysis. Data visualization and analysis were conducted using R, the IGV browser and GraphPad Prism V8. Statistical testing information is provided in Supplementary Table 6. Data meeting normality (*P* > 0.05, Shapiro–Wilk test) and equal variance criteria were subjected to two-sided Student’s *t*-tests (with *F*-tests for equal variance) or ANOVA (Brown Forsythe and Bartlett testing for equal variances), unless otherwise stated. When these assumptions were not met, appropriate nonparametric tests were chosen. ANOVA was followed by post hoc testing (Dunnett’s test for comparisons against the control; Tukey’s test for all pairwise comparisons) to calculate multiplicity-adjusted *P* values. For the analysis of clonal patch formation over time, generalized linear modeling (Poisson regression) was used. Model suitability was assessed by ensuring residual deviance was smaller than the degrees of freedom and checking for over-dispersion, where the variance of the response variable is not greater than the mean as assumed by the Poisson distribution. Diagnostic checks, including examination of deviance residuals and consideration of alternative models, were performed to assess goodness of fit and model adequacy. Clonal patch sizes were analyzed using linear modeling. In cases where data were not normally distributed, log transformation was applied to achieve normality. Normality of residuals was assessed using quantile-quantile (QQ) plotting and the Shapiro–Wilk test. Differential expression and accessibility analyses were done using default tests in LIMMA (two-sided modified *t*-test, Benjamini–Hochberg correction for multiple testing) and Seurat v.4.3.0 (two-sided nonparametric Wilcoxon rank-sum test, Bonferroni correction). UCell is based on the Mann–Whitney *U* statistic.

### Reporting summary

Further information on research design is available in the Nature Portfolio Reporting Summary linked to this article.

### Supplementary information

Supplementary Information Supplementary Discussion, Methods, References (also included in the main text) and Figs. 1–6.

Reporting Summary

Supplementary Tables Supplementary Tables 1–6.

Supplementary Data 1 List of sequences used for filtering ATAC-seq data.

### Source data

Source Data Fig. 1 Zipped folder with peak information and statistical source data.

Source Data Fig. 2 Panels 2d+e+f (font size).

Source Data Fig. 3 Panels 3c+f (font size).

Source Data Fig. 4 Zipped folder with motif analysis output.

Source Data Fig. 5 Zipped folder with statistical source data and Fig. 5c (font size).

Source Data Fig. 6 Statistical source data.

Source Data Fig. 7 Zipped folder with statistical source data and unprocessed western blots.

Source Data Fig. 8 Zipped folder with statistical source data and unprocessed western blots.

Source Data Extended Data Fig. 1 GO analysis results (GREAT).

Source Data Extended Data Fig. 2 Panel ExtData 2d (font size).

Source Data Extended Data Fig. 5 Statistical source data.

Source Data Extended Data Fig. 7 Statistical source data.

Source Data Extended Data Fig. 8 Statistical source data.

## Data Availability

The ATAC-seq datasets (GSE246646) and bulk RNA-seq data from TIMP1-treated hVSMCs and controls (GSE246647) have been deposited to the NCBI gene expression omnibus (GEO). The scRNA-seq datasets of VSMCs from mouse arteries after injury (GSE162167 (ref. ^20^)), mouse atherosclerosis (GSE155513 (ref. ^13^)) and human carotid plaque cells (GSE155512 (ref. ^13^)) are available from GEO. CAD GWAS^57^ was downloaded from the GWAS catalog. The *cis*-eQTL data are VSMC *cis*-eQTL from smooth muscle cells isolated from human umbilical cord (*N* = 1,499)^59^ and human aortic smooth muscle cell *cis*-eQTL for quiescent (*N* = 139) and proliferative (*N* = 145) cells^60^. Source data are provided with this paper.

## Code availability

Data analysis was done using available packages, as listed in Methods. Scripts used for GRN inference, rewiring calculation and analysis of published scRNA-seq datasets are available in GitHub (https://github.com/hfj22/Lambert_Oc_et_al_2024_NCVR).
