# Network-based prioritisation and validation of regulators of vascular smooth muscle cell proliferation in disease

**DOI:** 10.1038/s44161-024-00474-4

**Published:** 2024-06-06

**Authors:** Jordi Lambert, Sebnem Oc, Matthew D Worssam, Daniel Häußler, Charles U Solomon, Nichola L Figg, Ruby Baxter, Maria Imaz, James C K Taylor, Kirsty Foote, Alison Finigan, Krishnaa T Mahbubani, Tom R Webb, Shu Ye, Martin R Bennett, Achim Krüger, Mikhail Spivakov, Helle F Jørgensen

**Affiliations:** 1 Section of Cardiorespiratory Medicine, University of Cambridge, VPD Heart and Lung Research Institute, Papworth Road, Cambridge Biomedical Campus, Cambridge, CB2 0BB, UK; 2 Functional Gene Control Group, MRC Laboratory of Medical Sciences, Du Cane Road, London, W12 0NN, UK; 3 Institute of Clinical Sciences, Faculty of Medicine, Imperial College, Du Cane Road, London, W12 0NN, UK; 4 TUM School of Medicine and Health; Institute of Experimental Oncology and Therapy Research, Technical University of Munich, 81675 Munich, Germany; 5 Department of Cardiovascular Sciences, University of Leicester, and National Institute for Health Research Leicester Biomedical Research Centre, UK; 6 Collaborative Biorepository for Translational Medicine, Department of Surgery, University of Cambridge and NIHR Cambridge Biomedical Research Centre, Cambridge CB2 0QQ, UK; 7 Shantou University Medical College, Shantou, China; 8 Cardiovascular and Metabolic Disease Translational Research Programme, National University of Singapore, Singapore

## Abstract

Aberrant vascular smooth muscle cell (VSMC) homeostasis and proliferation characterise vascular diseases causing heart attack and stroke. Here, we elucidate molecular determinants governing VSMC proliferation by reconstructing gene regulatory networks from single-cell transcriptomics and epigenetic profiling. We detect wide-spread activation of enhancers at disease-relevant loci in proliferation-predisposed VSMCs. We compared transcriptional network rewiring between injury responsive and non-responsive VSMCs, which suggested shared transcription factors but differing target loci between VSMC states. Through *in silico* perturbation analysis, we identified and prioritised previously unrecognised regulators of proliferation, including RUNX1 and TIMP1. Moreover, we demonstrated that the pioneer transcription factor RUNX1 increased VSMC responsiveness and show that TIMP1 feeds back to promote VSMC proliferation through CD74-mediated STAT3 signalling. Both RUNX1 and the TIMP1-CD74 axis were expressed in human VSMCs, showing low levels in normal arteries and increased expression in disease, suggesting clinical relevance and potential as vascular disease targets.

Vascular smooth muscle cell (VSMC) proliferation underlies cell accumulation in atherosclerotic artery disease and vascular restenosis, that occurs following stenting and grafting. While genetic studies point to VSMCs as important hereditary determinants of cardiovascular disease^1–3^, their clinical targeting is presently unexplored. VSMC lesion investment in experimental atherosclerosis results from extensive clonal expansion of a small number of cells^4,5^. Similar VSMC oligoclonality has been demonstrated in other vascular disease models using genetic VSMC lineage-tracing^6,7^, and clonal VSMC contribution in human disease has been proposed^8,9^. Modification of VSMC clonality by altered cell-cell communication^10^ and ageing^11^ suggests that changes in activation frequency could underlie increased vascular disease risk.

Single cell RNA-sequencing (scRNA-seq) studies in mouse models and human vascular disease have shown remarkable transcriptional heterogeneity of VSMC-derived cells^12^. VSMC can adopt states that range from quiescence, with high expression of contractile genes (such as *MYH11* and *ACTA2*), in healthy arteries, to cells that have induced signatures of other cell types, including fibromyocytes (e.g. *TNFRSF11B*), macrophages (*LGALS3*), and chondrocytes (*RUNX2*) in addition to extracellular matrix (ECM) proteins and remodellers that are characteristic of the so-called "synthetic" state generated by classical VSMC phenotypic switching^13–16^.

Functional genomics in lineage-traced animals have shed light on genetic determinants and mechanisms regulating VSMC state changes in disease^1,2,17^. These experimental approaches have also revealed unexpected regulators of VSMC investment, such as the pluripotency factor OCT4^18^, and identified a mesenchymal VSMC-derived state marked by expression of VCAM1 and SCA1^13,16^. SCA1-positive VSMCs are found infrequently in healthy arteries, more abundantly in disease models and have been linked to VSMC priming and proliferation^14,16,19,20^. This molecular heterogeneity may explain the apparent distinct effects of VSMC regulators in different contexts^21,22^. However, the events governing activation of clonal VSMC proliferation are understudied^23^.

The integration of scRNA-seq data with epigenetic information led to identification of factors regulating developmental processes and disease^24^. Here we use this approach to model regulatory networks upon acute vascular injury when VSMC proliferation initiates. We find differential use of transcription factors in distinct VSMC transcriptional states along a proliferation-associated trajectory, possibly explaining context-specific effects of VSMC regulators. The analysis identifies known and candidate regulators that are prioritised using *in silico* simulation analysis. We functionally implicate RUNX1 and TIMP1 in driving activation of VSMC proliferation and suggest these mechanisms also operate in human disease development.

## VSMC activation is associated with de novo chromatin opening at distal sites linked to vascular disease-associated genes

**Figure 1:**
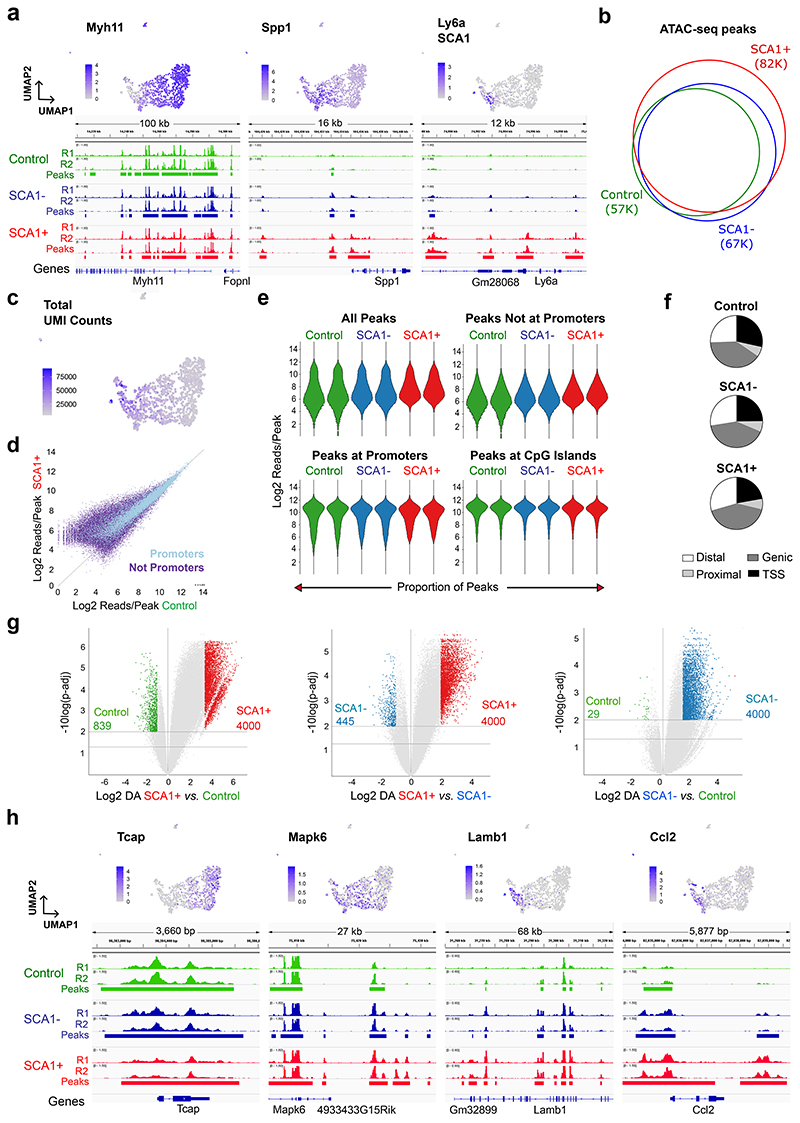
Activation of VSMCs results in widespread epigenetic activation of distal elements relevant for VSMC function and disease. **a**, ATAC-seq traces for markers of contractile VSMCs (*Myh11*), the synthetic phenotype (*Spp1*) and a VSMC transition state (*Ly6a*, encoding SCA1) in EYFP+ lineage-traced VSMCs isolated from healthy control Myh11-EYFP animals (green), and either EYFP+SCA1- (SCA1-, blue) or EYFP+SCA1+ (SCA1+, red) cells isolated from injured arteries. Aligned reads for independent experiments with cells from different animals (R1, R2) and reproducible peaks (horizontal bars) are shown. UMAPs (top) show associated normalised, log-transformed gene expression levels in scRNA-seq profiles of VSMC-derived cells 7 days after injury (GSE162167). **b**, Venn diagram representing overlap of ATAC-seq peaks in the three sample types. **c**, UMAP (as in **a**), showing the number of detected transcripts (unique molecular identifiers, UMI) per cell. **d**, Scatterplot showing read density in control (x-axis) and SCA1+ VSMCs after injury (y-axis) for ATAC-seq peaks at promoters (light blue) and peaks that are not at promoters (purple). **e**, Violin plots showing distribution of peak read densities in each sample for all peaks, or only non-promoter, promoter and CpG island peaks. **f**, Pie charts showing the proportion of peaks within 1 kb of transcriptional start sites (TSS), at promoter proximal regions (proximal), within genes (genic) or at intergenic elements (distal). **g**, Volcano plots showing fold change of peak intensity (differential accessibility, DA) between indicated samples. Horizontal lines indicate significance thresholds of p-adj<0.01 (top) and p-adj<0.05 (bottom). Differentially accessible peaks used for pathway enrichment analysis are highlighted (fold change>2 and of p-adj<0.01, or top 4000 ranked by fold change for SCA1+ cells (LIMMA-modified *t*-test, two-sided). The total number of peaks with increased accessibility in SCA1+ cells is 28K *vs*. Control and 14K *vs*. SCA1-). **h**, Gene expression and ATAC-seq data tracks (as in **a**) for genes showing higher accessibility in control samples (*Tcap*) or SCA1+ cells after injury (*Mapk6, Lamb1, Ccl2*).

To investigate the molecular regulation of VSMC proliferation, we elicited an injury response by carotid artery ligation, which leads to reproducible VSMC phenotypic switching and proliferation after 5-7 days^25^. Surgery was performed in Myh11-CreERt2, Rosa26-EYFP (Myh11-EYFP) animals after tamoxifen-induction of heritable reporter-EYFP expression in VSMCs to overcome the rapid loss of VSMC marker expression after injury. VSMCs expressing SCA1 have increased proliferative capacity and represent cells with pronounced phenotypic switching at this stage of injury^13,16,20^. We therefore mapped chromatin accessibility using bulk ATAC-seq in SCA1+ lineage-traced (EYFP+) VSMCs from animals 7 days after surgery and compared to EYFP+ VSMCs from no-injury littermate controls. We also profiled SCA1- cells from injured arteries, which include VSMCs in remodelled areas that show less extensive phenotypic changes and cells in adjacent carotid artery regions without obvious injury-responses. Samples from all three conditions (Control, injury SCA1+ and injury SCA1-) had high signal-to-noise ratio and strong replication of peak intensity (Fig. 1a, Extended Data Fig. 1a-c).

Accessibility changes were minor at *Myh11* and other contractile genes that are downregulated after injury (Fig. 1a), consistent with the documented retention of the active H3K4me2 marker at contractile genes in synthetic VSMCs^26,27^. Increased accessibility was found at e.g., *Spp1*, which is induced in both SCA1+ and SCA1- VSMCs, whereas the chromatin at the SCA1-encoding *Ly6a* locus became more accessible selectively in SCA1+ cells (Fig. 1a). Of all peaks detected across the three conditions, 96% were accessible in SCA1+ cells (Fig. 1b). This genome-wide *de novo* chromatin opening in SCA1+ (82K peaks) compared to control samples (57K) was consistent with the scRNA-seq transcript levels, which gradually increase across the VSMC population after injury (Fig. 1c).

ATAC-seq signal intensity was also generally higher in SCA1+ samples compared to control VSMCs (Fig. 1d), in particular at regions with lower accessibility, which is a characteristic of enhancers. Consistently, ATAC-seq peak intensity was shifted upwards for non-promoter peaks, but not changed for promoters and CpG islands, and the proportion of peaks at distal elements was also higher in SCA1+ cells, compared to control samples (Fig. 1e-f). Significantly higher signal intensity was detected at 28,303 peaks in SCA1+ samples compared to control VSMCs, whereas only 839 peaks were higher in controls compared to SCA1+ (Fig. 1g). The SCA1- sample showed intermediate results, possibly reflecting the heterogeneity of the cell population and the continuum of VSMC transcriptional states induced by injury^20^. Genes associated with regions selectively assessible in control cells were enriched for muscle contraction gene ontology (GO) terms (e.g. *Tcap*, Fig. 1h, Extended Data Fig. 1d). In contrast, genes associated with the regions with biggest chromatin accessibility-increase in SCA1+ compared to control samples were enriched for vascular diseases, including atherosclerosis, and biological process ontology terms linked to VSMC proliferation, such as regulation of signal transduction (*Mapk6*), inflammation (*Ccl2*) and cell adhesion (*Lamb1*)(Fig. 1h, Extended Data Fig. 1e-g). This analysis demonstrates that injury-induced VSMC activation results in opening of chromatin at enhancers of genes related to the functions of synthetic VSMCs and vascular disease, particularly in SCA1-expressing VSMCs.

## Rewiring of factors shared between injury-induced VSMC states

**Figure 2:**
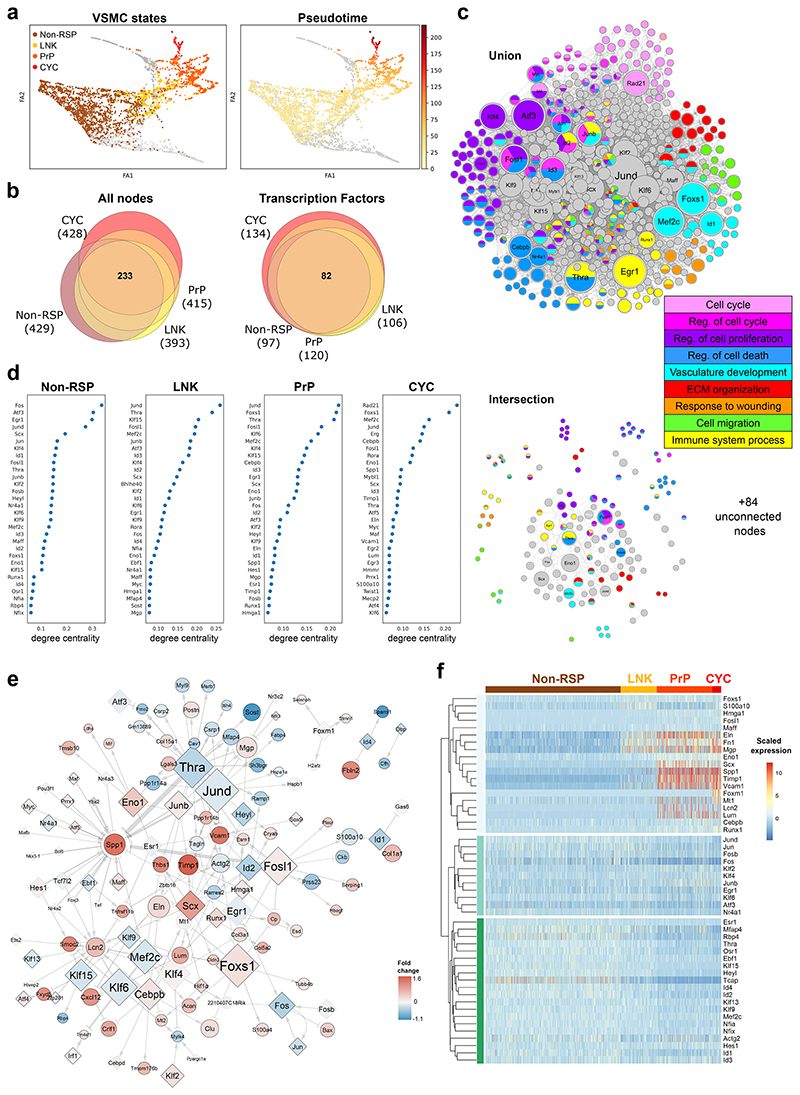
VSMC state-specific gene regulatory networks. **a** Force-directed graph representation of scRNA-seq data from VSMC-derived cells isolated 5 days after injury (GSE162167) shaded by VSMC states (left; Non-RSP: brown, LNK: yellow, PrP: orange and CYC: red, non-assigned: grey) or proliferation-associated pseudotime (right, yellow (low)-to-red (high) colour scale). **b**, Euler diagrams for all nodes (left) and transcription factors (right) in the four gene regulatory networks (GRNs) colour-coded as in panel **a**. The total number of nodes or transcription factors for each GRN is shown in brackets. **c**, The union (top) and intersection (lower panel) of Non-RSP, LNK, PrP, and CYC networks coloured by associated GO terms. The symbol size reflects node degree centrality scores separately in the union and intersection network. **d**, Nodes with top (30) degree centrality scores in each GRN. **e**, Network interactions in the PrP GRN with connectivity score>0.1. Differential expression between PrP and Non-RSP cells is indicated by a blue (higher in Non-RSP) to red (higher in PrP) colour scale. Black borders indicate significantly differential expression (p-adj<0.05, 2-sided Wilcoxon rank-sum test). Edge-widths reflect connectivity magnitude. **f**, Heatmap showing scaled expression for the genes with 50 highest rewiring scores for PrP *vs*. Non-RSP GRNs.

To identify factors controlling activation of VSMC proliferation, we compared how gene expression is orchestrated in VSMCs that respond to injury versus those that don't. We used scRNA-seq profiles of VSMCs analysed at the very onset of VSMC proliferation (5 days post injury), where we previously identified a proliferation-associated trajectory^20^. VSMCs were classified based on their position along this trajectory as "non-responding" ("*Non-RSP*", defined by persistent high levels of contractile gene expression), a "linking" cell population ("*LNK*"), leading on to a pre-proliferative state that includes cells expressing SCA1 ("*PrP*") and, finally, actively cycling cells that express *Mki67* and have high G2/S scores ("*CYC*") (Fig. 2a, Extended Data Fig. 2a).

To construct gene regulatory networks (GRNs) for these four VSMC states, we first generated a VSMC-specific transcription factor-target interaction network based on all detected ATAC-seq peaks, which was combined with gene expression profiles. This was done for each of the VSMC states (Non-RSP, LNK, PrP and CYC), using CellOracle^24^ and the resulting four GRNs had comparable centrality score distribution (Extended Data Fig. 2a). We found a substantial overlap between the networks, with 233/597 (37%) shared nodes, including 82/140 (59%) transcription factors (Fig. 2b, Supplementary Table 1a-b). Despite the high number of common nodes, few interactions were present in all GRNs (Fig. 2c, **lower panel**). Accordingly, abundant changes in the network topology rankings, such as degree centrality, were observed between networks (Fig. 2d, Supplementary Table 1c). GRN modelling using the scRNA-seq profiles of VSMCs analysed 7 days after injury was conducted in parallel and produced comparable results (Extended Data Fig. S2d).

The network nodes were enriched for processes related to VSMC biology or vascular injury, including ECM organisation, actin organisation, response to stimuli and regulation of cell proliferation (Fig. 2c, Supplementary Table 1b). Generally, specific regulatory circuits driving biological processes were not detected (Extended Data Fig. 2c). However, the CYC network included a substructure consisting of cell cycle-associated genes regulated by RAD21, a subunit of the cohesion complex that has roles in mitosis, genome stability and transcriptional regulation (Extended Data Fig. 2bc). The CYC network, which had the most unique factors (14 transcription factors, Fig. 2b), was dominated by genes driving cell cycle progression, such as *Myc, Mybl1, Erg* and *Rad21* (Fig. 2d). To study processes and factors driving activation, rather than progression, of VSMC proliferation we focussed on comparison to the PrP network (Fig. 2e).

Genes with most network changes in the PrP relative to the Non-RSP network were predominantly transcription factors (36/50) and fell into three groups by expression pattern (Fig. 2f). Group 1 increased expression along the injury-associated trajectory, and genes included markers of VSMC phenotypic switching (*Spp1, Fn1, Mgp*), *Vcam1* (that marks activated VSMCs^13^), *Timp1* and transcription factors *Scx, Cebpb, Runx1* and *Eno1*. Group 2 genes, which showed reduced expression along the trajectory, included *Mef2c, Thra, Hes, Heyl*, Kruppel like factors (KLF) 9, 13, and 15, and inhibitor of differentiation (Id) genes. Finally, group 3 genes showed little variation in expression between the non-responding and proliferation-associated states. This group contained factors reported to affect VSMC regulation, e.g. *Klf4, Egr1, Atf3*, and AP-1 subunits. We identified significant colocalization events between human VSMC eQTLs and genes with coronary artery diseases (CAD) GWAS signals for 13 human orthologues of the 50 genes with highest rewiring score (e.g. *KLF2/4/13/15, HEYL, TCAP, S100A10, VCAM1*, Supplementary Tables 1d-e), indicating causality of VSMC regulatory rewiring in disease.

## Injury-induced GRNs are relevant in atherosclerosis

**Figure 3:**
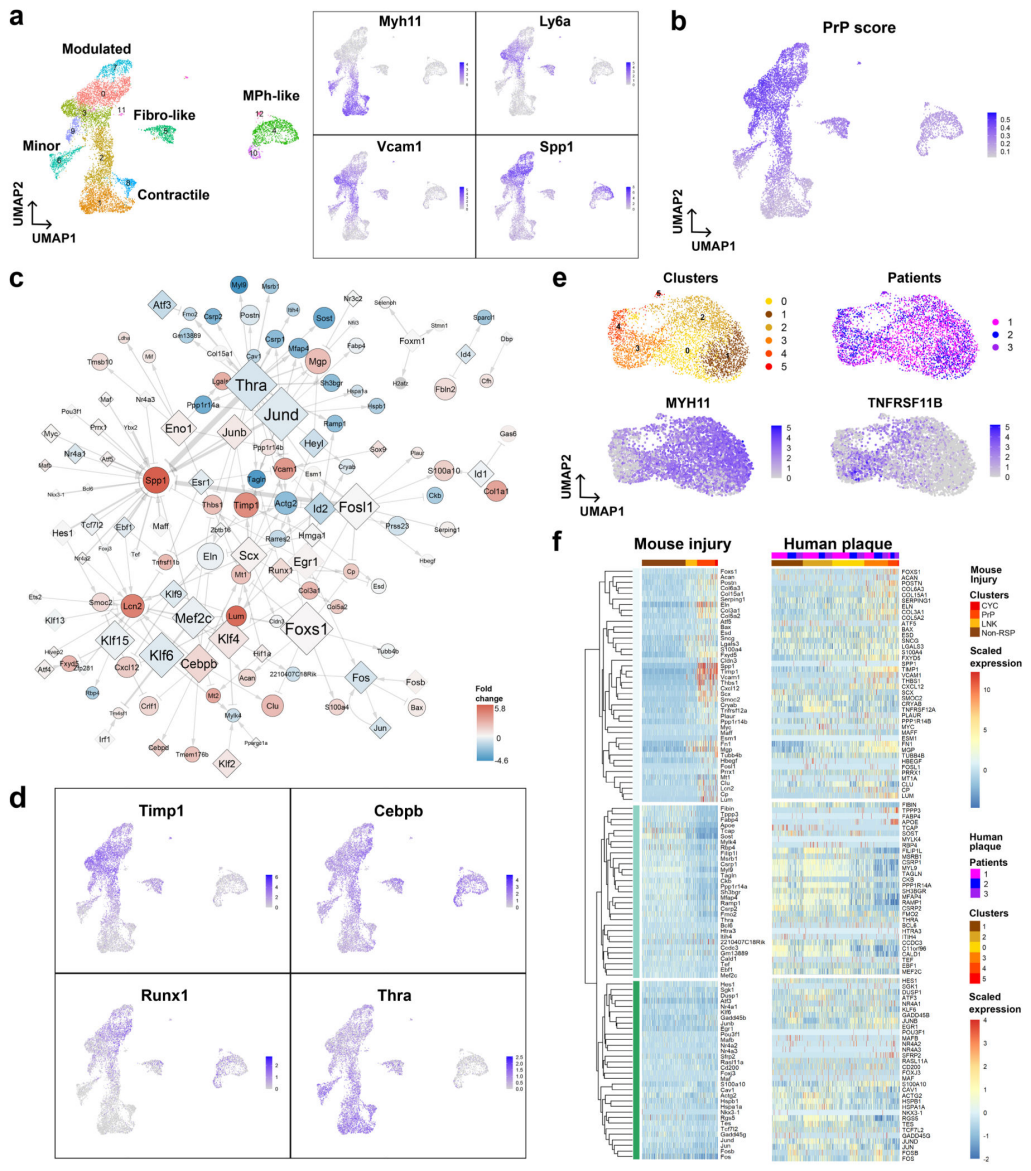
GRN-rewiring after injury aligns with gene expression changes in human and mouse atherosclerosis. **a-d**, Analysis of GRN node expression in experimental atherosclerosis using scRNA-seq profiles of lineage-traced VSMCs from high fat diet-fed *Apoe*-null animals (GSE155513). **a**, UMAPs showing cell clusters and annotation (left) and normalised, log-transformed expression of VSMC state marker genes (right). **b**, UMAP (as in **a**), showing UCell enrichment score of the PrP signature genes (increased expression in PrP *vs*. non-RSP cells, FC>0.5, p-adj<0.05, 2-sided Wilcoxon rank-sum test). **(c)** The PrP network, as shown in Fig. 2e, but the shading of nodes represent differential expression between modulated and contractile VSMC in the atherosclerosis mouse model (blue: higher in contractile (cluster 1), red: higher in modulated VSMCs, clusters 0+3). Black borders indicate significant differential expression (p-adj<0.05, 2-sided Wilcoxon rank-sum test). **(d)** UMAP (as in **a**), showing normalised, log-transformed expression for selected genes with high re-wiring score. **e-f**, Analysis of GRN node expression in VSMCs from a human carotid plaque scRNA-seq dataset (GSE155512). **e**, UMAPs annotated with cell clusters, patients, and normalised, log-transformed expression of contractile (*MYH11*) and modulated VSMC genes (*TNFRSF11B*). **f**, Heatmap showing scaled expression of genes ranked in the top 10 according to rewiring score (PrP *vs*. Non-RSP GRNs), and their direct strong interactors (connectivity score>0.1), in human carotid plaque VSMCs (right) and in the mouse injury dataset (left). Genes are clustered based on correlated expression along the mouse injury VSMC trajectory (left).

To assess the relevance of the regulation detected in injury to disease-associated VSMC changes, we used available datasets from VSMC-derived cells in experimental atherosclerosis^13^. First, we considered genes with higher expression in the PrP compared to the Non-RSP state in injury. Expression of this "PrP signature" was increased in VSMC subsets characterised by reduced expression of contractile genes (Fig. 3a-b, Extended Data Fig. 3a). Secondly, we found substantial overlap in transcript regulation between the two disease models for GRN nodes. Differential expression between contractile (cluster 1) and modulated VSMC clusters in atherosclerosis (clusters 0+3) was visualised on the PrP network (Fig. 3c), revealing similarity to the observed changes in injury (Fig. 2e). In general, transcription factors with increased expression in modulated VSMCs in atherosclerosis had positive connections to upregulated target genes in the PrP-GRN (e.g. *Sox9-Vcam1, Eno1-Tmsb10/Lgals3, Cebpb-Clu*). Vice versa, transcription factors with reduced expression in modulated *vs*. contractile VSMCs in atherosclerosis, such as Thra, had positive GRN interactions with genes that were also downregulated in modulated cells (e.g. *Csrp2, Mfap4, Sost, Myl9*, Fig. 3c). This analysis suggests similarity of the VSMC states in atherosclerosis and injury, and indicate that regulatory relationships mapped in injury are also relevant in atherosclerosis.

To compare with human disease, we isolated scRNA-seq profiles of mural cells from a published analysis of human carotid plaques^13^. The cells were clustered into 6 groups that were characterised by marker analysis (Fig. 3e, Supplementary Table 2b). Clusters 0-2 had abundant contractile gene expression, cluster 3 cells expressed *TNFRSF11B* that was identified as a fibromyocyte marker^14^, *VCAM1* was expressed mainly in clusters 3 and 4, and cluster 5 had increased levels of ribosomal proteins (Extended Data Fig. 3d-e). We then visualised the expression profiles for human orthologues of injury-rewired genes in the human cell clusters ordered by contractile gene expression. To capture regulatory relationships, we included the top 10 genes (by GRN rewiring score) and their direct interactors. Genes were visualised in the same order in human plaque and mouse injury cells (hierarchically clustered by their expression along the proliferation-associated trajectory, Fig. 3f). This side-by-side comparison showed similar expression patterns in mouse injury and carotid plaque VSMCs, indicating that the GRN analysis identified regulation that is relevant in human atherosclerosis.

## Prioritisation of candidate regulators of VSMC activation

**Figure 4:**
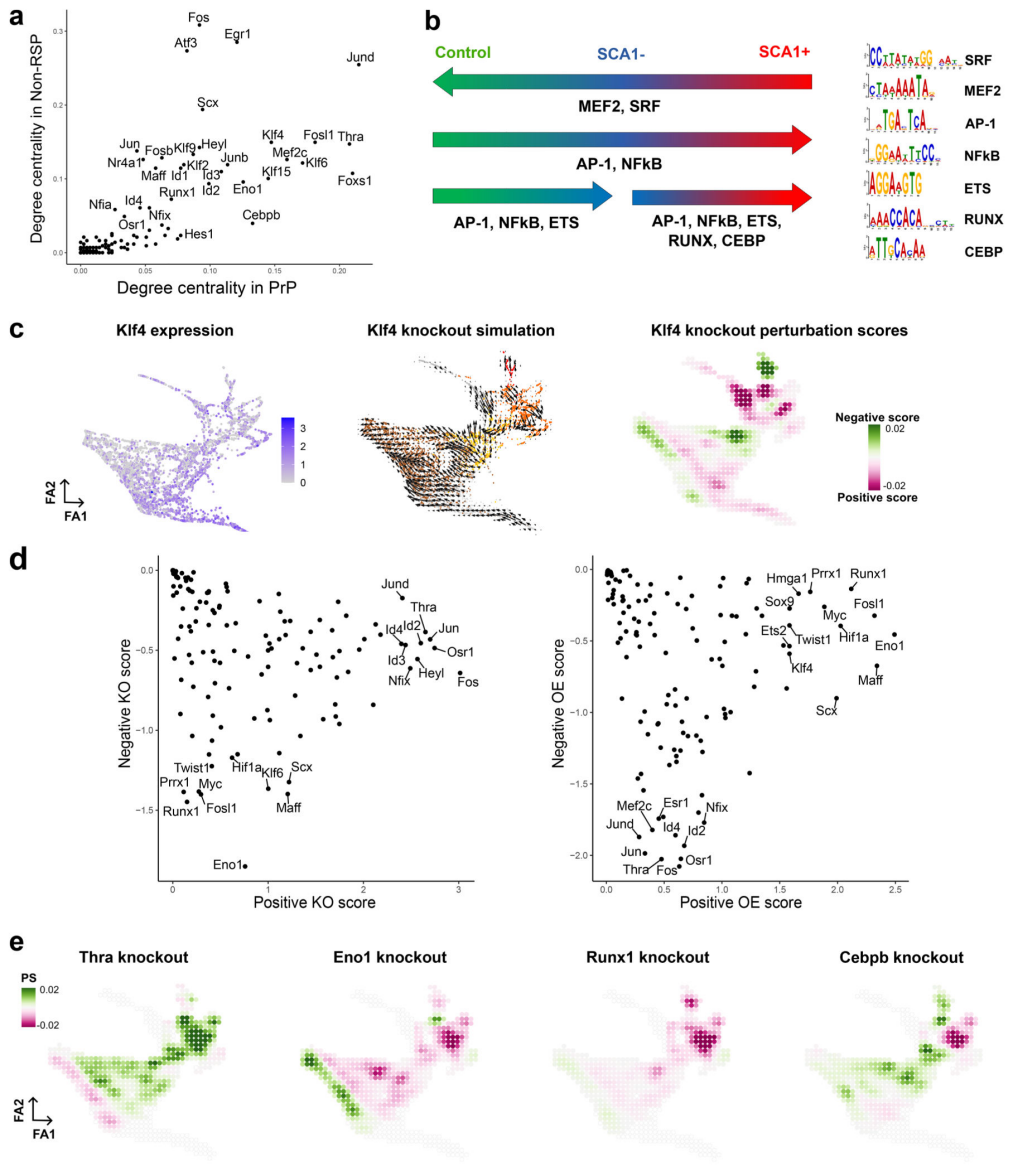
Rewiring of GRNs across cell states identifies candidate regulators of VSMC activation. **a**, Comparison of the out-degree centrality scores in PrP and Non-RSP GRNs indicating top scoring nodes. **b**, Summary of motif enrichment analysis for peaks showing higher accessibility relative to all peaks for the indicated comparisons (left). Detected motifs are shown on the right. **c**, Force-directed graph projections with shading showing normalised, log-transformed Klf4 expression (left) and simulation vector field (middle, colours indicate VSMC state as in Fig. 2a), or the perturbation score values (right) as predicted by *in silico* knockout of Klf4. **d**, Sum of positive *vs*. sum of negative perturbation scores for systematic *in silico* knockout (KO, left) and overexpression simulations (OE, right). Top-ranked transcription factors are highlighted. **e**, Force-directed graphs showing perturbation scores from *in silico* simulation of knockout phenotypes for indicated transcription factors.

Transcription factors changed topology score between the GRNs (Fig. 4a), prompting a motif enrichment analysis for ATAC-seq peak regions showing differential accessibility between conditions (Fig. 4b). The MEF2 and SRF motifs were enriched for peaks showing higher accessibility in control samples compared to SCA1+ VSMCs from injured arteries. Peaks that gained *de novo* accessibility after injury were enriched for AP-1, NFκB, CEBP, ETS and RUNX binding sites. Interestingly, RUNX, NFκB and CEBP motifs were also found to colocalise with AP-1 binding sites (Extended Data Fig. 4a), suggesting co-regulatory interaction at some loci.

The generated GRNs enabled computational simulation of how perturbation of transcription factor levels would affect cell state^24^. KLF4, a known regulator of VSMC phenotypic switching that promotes a macrophage-like state in atherosclerosis^28^, had high centrality in all networks apart from that of proliferating cells (CYC). *In silico* simulation of KLF4 depletion predicted a general shift towards the non-responding cell state, concomitantly with promotion of CYC cells (Fig. 4c). Such context-specific effects of KLF4 depletion are consistent with the delayed downregulation of contractile genes, but accelerated neointima formation in VSMC-specific KLF4 knockout after injury animals^21,21^.

To systematically predict function, we generated *in silico* perturbation scores for each transcription factor in the networks. Positive scores, reflecting stimulation of progression along the proliferation-associated trajectory^20^, and negative scores, indicating blocking effects, were calculated for loss- (knockout, KO) and gain-of-function (over-expression, OE) separately and showed strong correlation (R^2^>0.9, Extended Data Fig. 4b, Supplementary Table 1g). Transcription factors scoring highly for both positive and negative perturbation scores were predicted to have context-specific effects (Fig. 4d, Extended Data Fig. 4c-d). RUNX1, FOSL1, TWIST1, PRRX1 were predicted to mainly stimulate trajectory progression (Negative KO score < -1, positive KO score <0.75 (Fig. 4d-e, Extended Data Fig. 4c-d). The factors predicted to block trajectory progression (Positive KO score >1) were enriched for TGF-beta signalling (KEGG:04350), IL-17 signalling (KEGG:04657) and parathyroid hormone synthesis, secretion and action (KEGG:04928). Interestingly, thyroid hormone receptor alpha (THRA), that has been suggested to impact VSMC cholesterol metabolism^29^ was among trajectory-blocking factors (Fig. 4d). *Thra* is downregulated in PrP compared to Non-RSP VSMCs and negatively interacts with synthetic genes (*Spp1, Vcam1* and *Mgp*) in the PrP network, while promoting cytoskeletal genes (*Actg2, Pdlim4, Cdc42ep3*) in the Non-RSP GRN (Supplementary Table 1a). Simulation of THRA perturbation also suggested that THRA may safeguard the contractile state (Fig. 4e, Extended Data Fig. 4c-d).

Next, we used the GRN analysis to investigate how targets of this transcriptional rewiring may impact VSMC biology. To this end, we performed pathway analysis of the nodes with high target gene scores ("in-degree centrality") in the PrP or CYC GRNs that are also induced in PrP compared to Non-RSP cells (Supplementary Table 1c-d). This highlighted GO-terms including ECM organization (*Spp1, Vcam1, Cxcl2, Postn*) and regulation of cell adhesion (*Eln, Lum, Fbln2*). We also found modulators of proteolysis (*Timp1, Serpine1, Thbs1*) among the targets of rewired transcription factors, consistent with cellular niche remodelling by activated VSMCs.

Overall, this analysis highlights both known and putative VSMC regulators. To experimentally test factors highlighted by the GRN analysis, we selected RUNX1, as a candidate stimulating transcription factor, based on top ranking in the perturbation score analysis combined with motif enrichment in *de novo* accessible chromatin, and Tissue Inhibitor of Metalloproteinases-1 (TIMP1), as an example of a highly rewired target gene that, interestingly, was predicted to be indirectly induced by RUNX1.

## RUNX1 promotes VSMC proliferation

**Figure 5:**
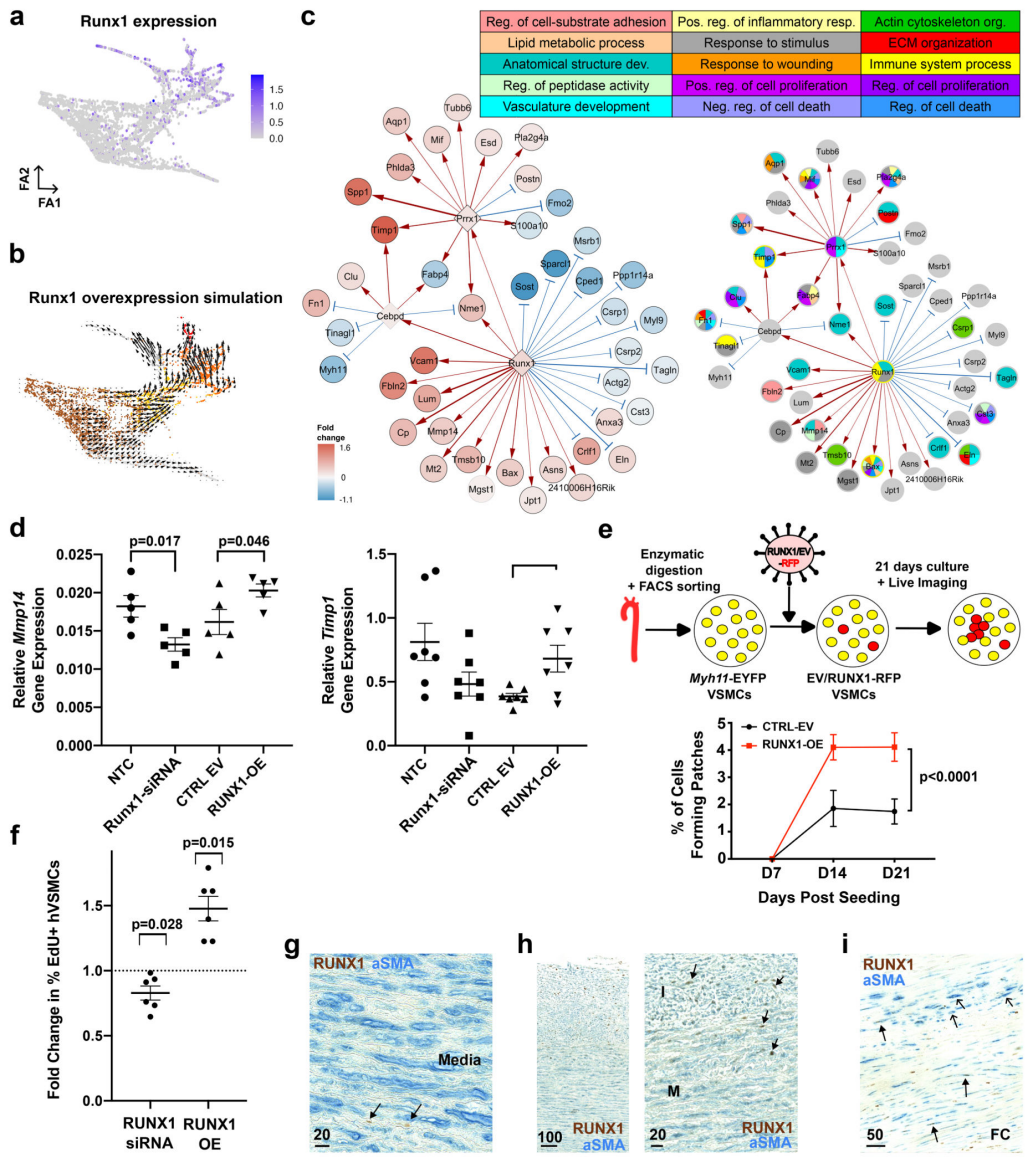
RUNX1-mediated regulation of VSMC proliferation. **a-b**, Force-directed graph of VSMCs isolated 5 days after injury showing normalised, log-transformed *Runx1* expression (**a**), or the result of RUNX1 overexpression simulation (**b**, colours indicate VSMC state as in Fig. 2a). **c**, Direct and indirect targets of Runx1 in the PrP GRN showing differential expression between PrP and Non-RSP cells on a blue (higher in Non-RSP) to red (higher in PrP) scale, (left, black borders indicate significantly differential expression, p-adj<0.05, 2-sided Wilcoxon rank-sum test) or gene ontology terms for nodes (right). Edge widths reflect connectivity magnitude and colour show positive (red) and negative (blue) interactions. **d**, *Mmp14* and *Timp1* transcript levels detected by quantitative RT-PCR in lineage-labelled VSMCs transfected with non-targeting (NTC) or *Runx1*-targeting siRNA (Runx1-siRNA), or transduced with an empty vector (CTRL EV) or RUNX1-overexpressing (RUNX1-OE) lentivirus. Dots represent values from independent animals (N=5), lines mean and error bars SEM, p-value: two-tailed *t*-test or Mann-Whitney U. **e**, Schematic of clonal proliferation assay; red fluorescent protein (RFP)-expressing lentivirus is used to test the effect of RUNX1 cDNA relative to an empty control vector (EV) on the ability of lineage-labelled VSMCs from Myh11-EYFP animals to form colonies (left). Right panel shows the percentage of cells forming a clonal VSMC patch in RUNX1-OE and empty vector control cells (CTRL-EV). Points indicate mean (N=4 animals analysed in triplicate), line show mean, error bars SEM, p-value=1.97e-12, generalised linear model. **f**, Fold change in %EdU+ cells after siRNA-mediated RUNX1 depletion (RUNX1-siRNA, relative to non-targeting siRNA-treated cells) and lentivirus-mediated RUNX1-overexpression in hVSMCs (RUNX1-OE, relative to cells transduced with empty vector virus). Points show averages of quadruplicate replicates for independent hVSMC isolates (N=6 donors), lines indicate mean, error bars SEM, p-value: two-tailed *t*-test. **g-i**, Representative immunostaining of non-plaque aorta (**g** and **h**, N=10 donors) or carotid endarterectomy samples (**i**, N=6 donors) for RUNX1 (brown) and αSMA (blue). Scale bars: **g**: 20 µm, **h**: 100 µm (left, middle) and 20 µm (right), **i**: 50 µm. FC: fibrous cap, I: Intima, M: Media. Examples of RUNX1+ (closed arrowheads) and RUNX1- αSMA+ cells (open arrowheads) are indicated.

The GRN analysis highlighted RUNX1, which belongs to the Runt-related transcription factor family and plays a key role in haematopoiesis, as a potent stimulator of VSMC activation. Consistently, *Runx1* transcripts were increased in the PrP and CYC states compared to non-responding cells, increased chromatin accessibility was detected at both *Runx1* promoters in SCA1+ VSMCs compared to the control samples, and we detected RUNX1 in lineage-labelled VSMCs after injury (Fig. 5a, Extended Data Fig. 5a-b). In contrast, the RUNX family members *Runx2* and *Runx3* were lowly expressed at this stage of VSMC activation (Extended Data Fig. 2a). The RUNX motif was also highlighted in relation to VSMC regulation by studies in experimental atherosclerosis^1^ and *RUNX1* transcript levels were higher in modulated compared to contractile VSMCs in mouse and human atherosclerosis (Figure 3d, Extended Data Fig. 3f).

GRN-based simulation of elevated RUNX1 expression predicted that RUNX1 promotes the transition towards VSMC proliferation (Fig. 5b), as expected from the limitation of proliferation observed when simulating *Runx1*-knockout (Fig. 4e, Extended Data Fig. 4c-d). The PrP GRN predicted that RUNX1 stimulates injury-induced genes, including *Vcam1, Lum* and *Mmp14*, and GO analysis of all direct and indirect RUNX1 target genes suggested that RUNX1 could affect cell-substrate adhesion (*Fbln2, Mmp14*), response to stimulus (*Cp, Mt2*) and ECM organisation (*Eln*) (Fig. 5c). Interestingly, four of the 15 direct RUNX1 target genes were previously associated with activated, SCA1+ VSMCs in healthy arteries^16^. We also found that expression of the predicted RUNX1 target, MMP14 is a hallmark of SCA1+ VSMCs (Extended Data Fig. 5c), further indicating that RUNX1 supports a state that is primed for proliferation.

We experimentally tested how RUNX1 affects gene expression in freshly isolated lineage-traced VSMCs. Lentiviral-induced RUNX1 overexpression significantly increased expression of predicted direct targets *Mmp14, Timp1* and *Cebpd*. Conversely, siRNA-mediated depletion of *Runx1* significantly reduced *Mmp14* and blunted *Timp1* expression (Fig. 5d, Extended Data Fig. 5d-e). To test whether RUNX1 affects activation of VSMC proliferation, we adapted an *in vitro* proliferation assay that reproduces the low frequency clonal VSMC expansion observed in vascular disease models^20^. RFP-expressing lentivirus was introduced in lineage-labelled EYFP+ VSMCs at low titre and expanding RFP/EYFP double-positive clonal VSMC patches identified by repeated live cell imaging over three weeks. This showed significantly increased clonal patch-forming ability of RUNX1-overexpressing *vs*. control cells (Fig 5e).

In human VSMCs (hVSMCs), RUNX1-depletion decreased the percentage of EdU+ cells and, *vice versa*, overexpression of RUNX1 increased the percentage of EdU+ cells, relative to their respective controls. This suggests that stimulation of VSMC proliferation by RUNX1 is conserved in human (Fig. 5f, Extended Data Fig. 5f). To assess RUNX1 functions in human disease, we immunostained arteries without detectable plaques but displaying varying degrees of intimal thickening and carotid plaques. RUNX1 protein was detected infrequently in alpha smooth muscle actin (αSMA)+ cells in arteries with a healthy, organised morphology of the medial layer, but a higher proportion of medial RUNX1+αSMA+ cells were observed in arteries with evidence of perturbation (Fig. 5g). For example, in arteries displaying intimal thickening, many αSMA+ cells within the intima expressed RUNX1 (Fig. 5h, Extended Data Fig. 5g). Substantial heterogeneity in RUNX1 expression was observed in human atherosclerotic lesions, interestingly however, αSMA+RUNX1+ cells were also observed in the fibrous cap (Fig. 5i). Jointly, these results demonstrate that RUNX1 regulates gene expression in VSMCs and promotes their proliferation, and indicates that RUNX1 is implicated in human disease development.

## TIMP1 promotes VSMC proliferation in an MMP-independent manner

**Figure 6:**
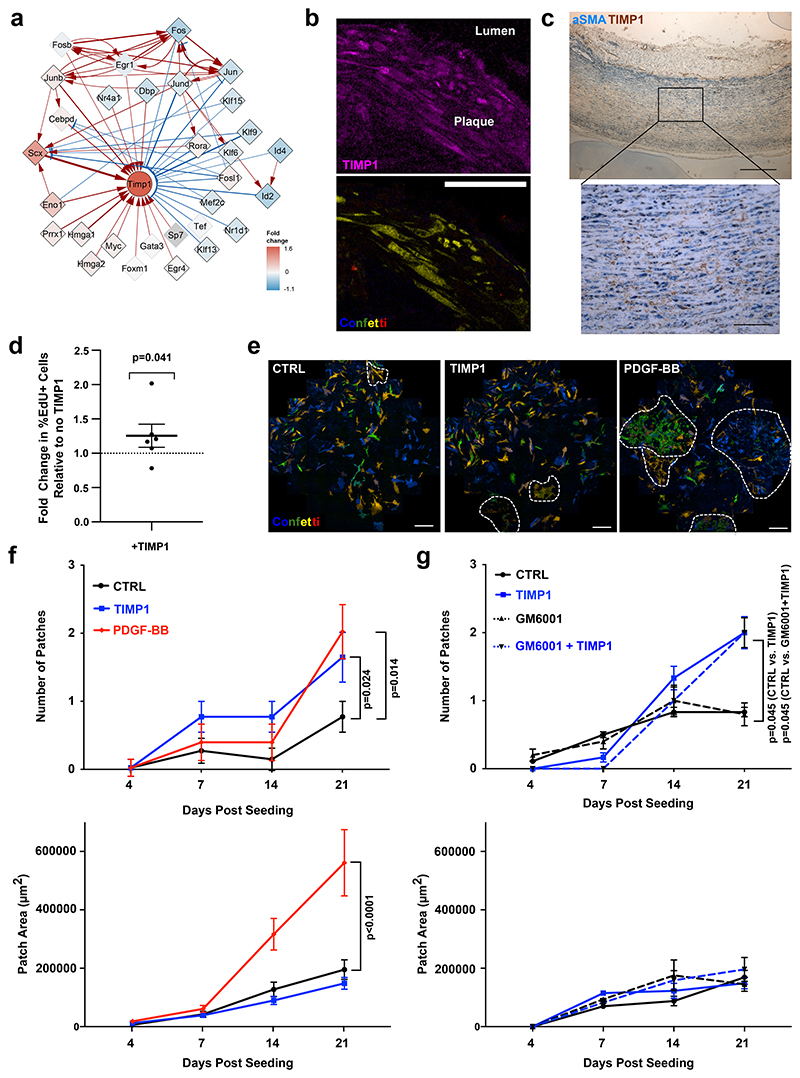
GRN analysis identifies TIMP1 as a functional gene target and driver of VSMC proliferation. **a**, TIMP1 connections in the PrP GRN annotated as Fig. 6c. **b**, TIMP1 immunostaining of atherosclerotic lesion from Myh11-Confetti/Apoe animals showing signals for lineage-labelled VSMCs (Confetti), TIMP1 (magenta), and nuclear DAPI staining (white) in a single confocal z-section. Representative of 3 animals. Scale bar=50 µm. **c**, Representative immunohistochemistry image for αSMA (blue) and TIMP1 (brown) in non-plaque human aorta (N=7 donors), scale bar=500 µm (overview),100 µm (zoomed view). **d**, Fold change in %EdU+ hVSMCs following 16 hours EdU incorporation in cells treated with 500 ng/mL rhTIMP1 relative to vehicle controls. Dots indicate average of independent hVSMC isolates (N=6 donors), line show mean, error bars SEM, p-value: two-tailed *t*-test. **e**, Representative images of lineage-labelled VSMCs isolated from Myh11-Confetti aortas and cultured for 21 days in the presence of vehicle control, 500 ng/mL recombinant murine TIMP1 or 2 ng/mL PDGF-BB. Scale bar=500 µm. **f-g**, Quantification of number and size of clonally expanded patches of Confetti+ VSMCs over 21 days of culture. Points indicate mean (N=4 animals, triplicate analysis of cells from each animal), error bars SEM, p-value= 5.2e-06, generalised linear model.

TIMP1 was among the most central nodes and was selected among the targets of regulatory rewiring as increased TIMP1 protein levels have been associated with cardiovascular disease and severity^30,31^. In the PrP GRN, TIMP1 was predicted to be induced by multiple factors including Cebpd, Myc, Prrx1 and Scx (Fig. 2e, 6a), and an indirect RUNX1 target (see above). TIMP1 transcripts were also upregulated by VSMCs in human and mouse atherosclerosis scRNA-seq datasets (Fig. 3d, Extended Data Fig. 3f and ^1,13,16^).

TIMP1 protein was expressed in clonally expanded VSMCs in both murine atherosclerotic and injury-induced lesions and also detected in αSMA+ cells located in the medial layer of non-plaque human arteries (Fig. 6b-c, Extended Data Fig. 6a-b). This suggested TIMP1 could affect VSMCs at an early disease stage. To investigate the potential impact of TIMP1 protein on VSMC function, we performed bulk RNA-seq in TIMP1-treated *vs*. control hVSMCs. This revealed significant induction of cell cycle genes (E2F targets and G2/M checkpoint), fatty acid metabolism and oxidative phosphorylation gene sets in TIMP1-treated cells (Extended Data Fig. 6c), indicating impact on metabolism and cell proliferation. We therefore tested the impact of TIMP1 on VSMC proliferation, as the role of TIMP1 is controversial^32–34^. We found a small, but significant, increase in %EdU+ cells in TIMP1-treated hVSMCs versus controls (Fig. 6D), similar to a recent study^34^. TIMP1-treatment also stimulated clonal expansion of quiescent VSMCs isolated directly from aortas of lineage-labelled Myh11-Confetti animals (Fig. 6e-f). TIMP1 increased the frequency of VSMC clone formation to a similar degree as PDGF-BB treatment (Fig. 6f). However, TIMP1 did not change the size of clonal patches, in contrast to PDGF (Fig. 6f, **lower panel**), suggesting that the establishment and growth of VSMC clones are regulated independently.

TIMP1 functions both as a matrix metalloproteinase (MMPs) inhibitor and as a cytokine^35,36^. We found no effect of a broad-spectrum MMP inhibitor (GM6001) on clone dynamics in control nor in TIMP1-treated cells (Fig. 6g). This suggested that the ability of TIMP1 to promote clonal VSMC proliferation is not linked to inhibition of proteolysis, similar to in established hVSMC cultures^32^. Taken together, we conclude that TIMP1 stimulates clonal VSMC proliferation in an MMP-independent manner.

## TIMP1 induces STAT3 phosphorylation to induce VSMC proliferation

**Figure 7:**
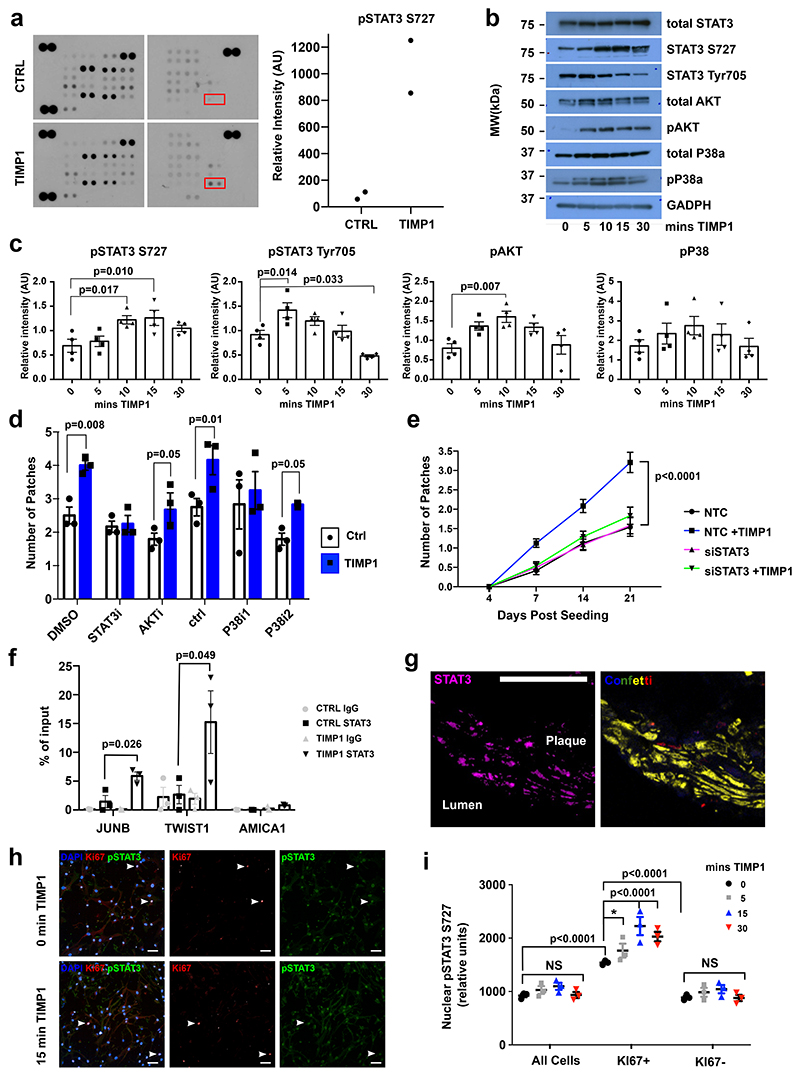
TIMP1 signalling induces STAT3 phosphorylation in human and mouse VSMCs. **a**, Phosphokinase array and densitometric quantification of serum-starved human VSMCs (hVSMCs) treated for 15 minutes with 500 ng/mL rhTIMP1 or vehicle control (N=1 donor). **b**, Western blot of total, phospho-STAT3 (S727 or Tyr705) STAT3, total and phospho-AKT, total and phospho-P38 (pP38), and GAPDH in serum-starved hVSMCs after 0, 5 10, 15 or 30 minutes rhTIMP1. **c**, Quantification of relative western blot band intensity, normalised to GAPDH. Points show independent hVSMC isolates (N=4), bars means, error bars SEM, p-value: one-way ANOVA. **d**, Number of clonally expanded patches formed by lineage-labelled VSMCs following 21 days of culture, without (white, circles) or with 500 ng rmTIMP1 (blue, squares) in samples treated with vehicle (DMSO), 10 µM TT101 (STAT3i), 100 nM MK-2206 (AKTi), 10 µM SB202474 (control inhibitor, ctrl), 10 µM SB203580 (p38 i1), or 10 µM SB202190 (p38 i2). Points show average (N=3 Myh11-Confetti animals analysed in triplicate), bars the mean, error bars SEM, p-value: two-tailed *t*-test. **e**, Quantification over time of clonally expanded patches formed by lineage-labelled VSMCs, treated with non-targeting control (NTC) or *Stat3*-targeting siRNA (siSTAT3) +/-500 ng rmTIMP1. Points indicate mean (N=3 Myh11-Confetti animals analysed in triplicate), error bars SEM, p-value= 2.2e-07, generalised linear model. **f**, ChIP-qPCR analysis at STAT3 targets (*TWIST* and *JUNB*) and negative control (*AMICA1*), in serum-starved control and rhTIMP1-treated hVSMCs (15 minutes, 500 ng/mL), showing anti-STAT3 and control-IgG precipitated DNA as a percent of input. Bars show mean of independent hVSMC isolates (N=3 donors), error bars SEM, p-value: two-tailed *t*-test. **g**, pSTAT3 S727 immunostaining (magenta) in cryosections from carotid plaque in Myh11-Confetti/Apoe animal (11 weeks HFD) with DAPI (white) and Myh11-Confetti signals (CFP: blue, RFP: red, YFP: yellow, GFP: green). Representative of 3 animals. Scale bar=50 µm. **h-i**, pSTAT3 S727 and KI67 immunostaining (**h**) and quantification (**i**) of serum-starved control hVSMCs +/- 500 ng/mL rhTIMP1 treatment (15 minutes, **h**) or indicated time points (**i**). Arrowheads indicate KI67+ cells. Scale bar=50 µm. Symbols show averages values for independent hVSMC isolates (N=3 donors, analysed in triplicate) lines show mean, error bars SEM, p-value: two-way ANOVA, *p=0.0498.

The signalling pathways activated by TIMP1 show context-dependency^34,36,37^, so we screened for TIMP1-mediated phospho-kinase activation in hVSMCs. This suggested that STAT3 phosphorylated at S727 is strongly increased in TIMP1-treated cells, which was confirmed by western blotting in independent hVSMC isolates (Fig. 7a-b, Extended Data Fig. 7a). Increased phosphorylation was observed at both S727 and Tyr705, while total STAT3 protein levels were unchanged (Fig. 7b-c, Extended Data Fig. 7b-c). The rapid and transient induction of STAT3 phosphorylation suggests a direct response to TIMP1 (Fig. 7b-c). We also analysed the effect of TIMP1 on signal transducers known to regulate VSMC growth. Similar to STAT3, phosphorylation of AKT was induced by TIMP1, whereas P38 phosphorylation levels were variable between hVSMCs from different individuals (Fig. 7b-c, Extended Data Fig. 7b-c).

The role of these signalling pathways in TIMP1-mediated stimulation of clonal VSMC proliferation was investigated by addition of small molecule inhibitors in the clonal VSMC proliferation assay. Inhibition of STAT3 using TT-101 abolished the increased clone number in response to TIMP1, whereas an impact of AKT and P38 inhibition was less obvious (Fig. 7d). To further test the specificity, we depleted STAT3 using siRNA, which also prevented the TIMP1-mediated increase in clone formation (Fig. 7e). Phosphorylation of STAT3 leads to dimerization, nuclear import and binding at target gene promoters^38^. Consistent with its activation, STAT3 chromatin immunoprecipitation showed increased binding in TIMP1-treated cells, specifically at promoters of canonical STAT3 target genes (JUNB, TWIST1), compared to control cells (Fig. 7f).

VSMC-derived cells in atherosclerotic plaques and neointimal lesions expressed high levels of pSTAT3 compared to medial cells (Fig. 7g, Extended Data Fig. 7d), indicating its involvement in clonal VSMC expansion. To further assess whether STAT3 phosphorylation is linked with VSMC proliferation, we co-stained hVSMCs for KI67 and pSTAT3 (S727) after TIMP1 treatment. Overall, KI67+ cells had higher nuclear pSTAT3 levels compared to KI67- cells and there was significantly increased pSTAT3 expression 15 minutes after TIMP1 stimulation (Fig. 7h, i). There was no effect of TIMP1 on hVSMC proliferation within this time frame (Extended Data Fig. 7e). We next analysed VSMCs isolated from aortas of lineage-labelled mice and cultured to mimic phenotypic switching and activation of proliferation. STAT3 phosphorylation was also induced by TIMP1 after four days of culture, but this effect was less pronounced in cells analysed at day 7 (Extended Data Fig. 7f). Interestingly, baseline pSTAT3 intensity was higher in cells cultured for four days after isolation, compared to day 7 cells, suggesting the existence of a "window of opportunity" with higher responsiveness to TIMP1. Overall, this data links TIMP1-mediated STAT3 phosphorylation to the activation of VSMC proliferation.

## TIMP1 signals through CD74 to induce VSMC proliferation

**Figure 8:**
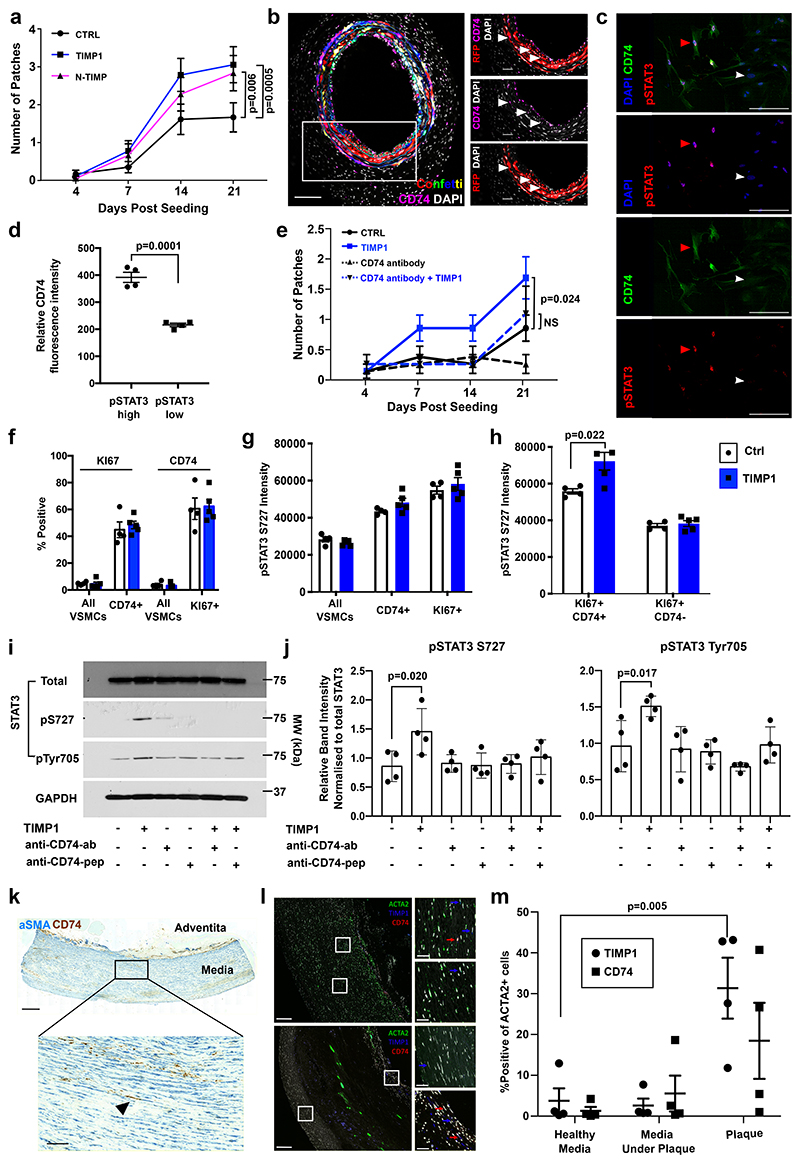
TIMP1 signals to STAT3 via CD74 in a disease-relevant mechanism. **a**, Number of clonal patches formed by lineage-labelled VSMCs, treated with 500 ng/mL recombinant TIMP1 or equimolar N-TIMP1 over 21 days culturing. Points indicate mean (N=4 Myh11-Confetti animals analysed in triplicate), error bars SEM, p-value: generalised linear model. **b**, CD74 immunostaining (magenta), Myh11-Confetti signal and DAPI nuclear staining (white) in carotid arteries 10 days post ligation. Magnified view of boxed region shows only the RFP Confetti reporter. White pointers mark CD74/RFP double-positive cells. N=5 Myh11-Confetti animals. Scale bar=100 µm (overview), 30 µm (zoom). **c**, Immunostaining for pSTAT3 S727 (red) and CD74 (green) with nuclear DAPI (blue) in mVSMCs treated with 500 ng/mL rmTIMP1 four days post isolation. Arrowheads mark STAT3-high (red) and STAT3-low cells (white). Scale bar=50 µm. **d**, Quantification of cellular CD74 levels in **c**, stratified by nuclear pSTAT3 S727 intensity. Dots show average per animal (N=4 animals analysed in quadruplicate), lines means, error bars SEM, p-value: two-tailed *t*-test. **e**, Number of clonal patches formed by lineage-labelled VSMCs +/-500 ng/mL rmTIMP1 and/or CD74-blocking antibody/peptide. Points indicate mean (N=4 Myh11-Confetti animals analysed in triplicate), error bars SEM, p-value: generalised linear model (CTRL as base variable). Independent generalised linear modelling (TIMP1 as base variable) show significant difference between TIMP1 and CD74-antibody+ TIMP1 (p=0.049). **f-h**, Quantification of imaging flow cytometry of lineage-labelled aortic VSMCs from 13-week old, HDF-fed (4 weeks) Myh11-EYFP/Apoe animals treated with rmTIMP1 or vehicle control. Symbols show values for individual animals (N=4 (ctrl), 5 (TIMP1)), bars mean, error bars SEM, p-value: two-tailed *t*-test. **f**, Percentages of all VSMCs, or indicated sub-populations, expressing KI67 and CD74. **g-h**, pSTAT3 S727 median fluorescence intensity. **i-j**, Representative western blot (**i**) and quantification (**j**) of serum-starved control and rhTIMP1-treated hVSMCs (5 minutes) +/- pre-treatment with CD74-blocking antibody (CD74 ab) or peptide (CD74 pep). Points show independent hVSMC isolates (N=4 donors), lines means, error bars SEM, p-value: one-way ANOVA. **k**, CD74 (brown) and αSMA (blue) immunohistochemistry in non-plaque human aorta. Scale bar=500 µm (overview), 100 µm (zoom). N=7 donors. **l**, RNA *in situ* hybridization for *ACTA2* (green), *TIMP1* (blue) and *CD74* (red) in human healthy aorta, and plaque-containing carotid. Arrows indicated *TIMP1*/*ACTA2*+ (red) and *CD74*/*ACTA2*+ cells (blue). Scale bar=250 µm (overview), 50 µm (zoom). **m**, Quantification of *TIMP1* or *CD74* expression in *ACTA2*+ cells in non-plaque aortas (Healthy media) or carotid endarterectomy regions (Media Under Plaque, Plaque). Symbols show different donors (N=4 donors/condition), lines means, error bars SEM, p-value: one-way ANOVA.

Signalling by TIMP1 can be executed *via* several surface receptors that bind to different TIMP1 protein domains^37^. We found that the ability to induce clonal VSMC proliferation was retained by the N-terminal part of TIMP1 (Fig. 8a), which binds the HLA class II histocompatibility antigen gamma chain, CD74^39^ that has also been detected in modulated, or "macrophage-like" VSMCs in experimental atherosclerosis^40^.

Immunostaining revealed that Myh11-lineage labelled VSMCs also express CD74 protein after vascular injury *in vivo* (Fig. 8b and Extended Data Fig. 8a), providing evidence that TIMP1 could function via CD74 in VSMCs, as suggested^34^. CD74 was also detected in cultured lineage labelled mVSMCs (Fig. 8c); we found an association between high pSTAT3 and greater CD74 expression (Fig. 8c-d), indicating that CD74 signalling results in phosphorylation of STAT3. To directly test whether TIMP1 acts via CD74 in VSMCs, we used a CD74 blocking antibody, which abolished TIMP1-augmented VSMC clone formation (Fig. 8e). Interestingly, anti-CD74 also reduced the frequency of clone formation in untreated cells, which could be due to blocking the effect of TIMP1 secreted by VSMCs after culture-induced phenotypic switching, or other CD74 ligands such as MIF. Blocking CD74 in VSMCs overexpressing RUNX1 also abolished the stimulation of proliferation (Extended Data Fig. 8b), suggesting that the observed increase in *Timp1* expression could be important for the RUNX1-OE phenotype.

To assess whether the TIMP1-CD74-pSTAT3 axis is active in atherosclerosis, we investigated lineage-labelled *Apoe*-/- animals after a 4-week exposure to high fat diet, at a timepoint prior to robust VSMC contribution in lesions^10,20^. Imaging flow cytometry showed a low frequency of KI67+ lineage-labelled VSMCs, as expected, and this varied between animals. We found a striking enrichment of CD74-expression in KI67+ cells (Fig. 8f) and the levels of phospho-STAT3 were higher in proliferating (KI67+) and in CD74+ VSMCs, compared to all lineage-labelled cells (Fig. 8g). This suggests that CD74 and phosphorylation of STAT3 are linked to VSMC proliferation. TIMP1 treatment further increased pSTAT3 levels in CD74+, but not in CD74- proliferating VSMCs (Fig. 8h, Extended Data Fig. 8c), providing *in vivo* evidence linking this signalling pathway to initiation of VSMC proliferation in atherosclerosis.

Next, we explored the TIMP1-CD74-pSTAT3 axis in human. As shown in Fig. 8i, TIMP1-induced STAT3 phosphorylation was reduced in cells co-treated with the anti-CD74 antibody and a similar effect was observed using a CD74-blocking peptide^41^, demonstrating that TIMP1-mediated induction of STAT3-phosphorylation is downstream of CD74 (Fig. 8i-j). We found that CD74 was detected by immunostaining in αSMA+ cells in non-plaque arteries from human organ donors and in samples taken after carotid endarterectomies (Fig. 8k, Extended Data Fig. 8d). To quantify expression, we performed multi-probe *in situ* hybridisation (Fig. 8l-m). *TIMP1* and *CD74* transcripts were detected in *ACTA2*+ VSMCs in non-plaque arteries, but observed at low frequency. Compared to medial cell without lesions, CD74 and TIMP1 expression was elevated in lesions and quantification revealed substantial co-expression with ACTA2 (12-43% of ACTA2+ plaque cells expressed TIMP1; 1-41% for CD74). Altogether, these findings suggest that the induced expression of TIMP1 upon VSMC activation could stimulate VSMC proliferation by binding to CD74 via activating STAT3 phosphorylation.

## Discussion

We here used GRN modelling after acute vascular injury to identify factors governing activation of VSMC proliferation, which could be important to reduce vascular disease susceptibility and for disease prevention strategies. Our analysis indicates that differential use of shared transcription factors plays an important role in this process. This also suggests that VSMC state-dependent mechanisms may underlie observed context-specific functions, such as the dichotomous function of the MCP-1 factor, encoded by *Ccl2*^22^. Using *in silico* simulation experiments, we prioritise both factors known to control VSMCs in disease and candidate regulators proposed to safeguard the contractile state (e.g. THRA) or promote cell activation (e.g. RUNX1, CEBP/D, ENO1). We experimentally validate RUNX1 and the TIMP-STAT3-CD74 axis *in vivo* and *in vitro* as VSMC regulators with relevance to human diseases. Genetic evidence linking these factors to vascular disease includes a variant in the RUNX1 locus associated with stroke (rs116262092-A) and colocalization of a STAT3 VSMC eQTL with a CAD GWAS signal (Supplementary Table 1g). Furthermore, vascular abnormalities and increased aneurysm risk have been identified in patients with missense STAT3-variants causally implicating this pathway in vascular regulation^42,43^.

RUNX1 has well-known functions in haematopoiesis and also impacts differentiation and proliferation in other contexts, including cardiovascular cells^44,45^. We here show that RUNX1 increases the frequency of VSMCs that start proliferating, possibly by directly inducing the expression of VSMC "transition state" genes, such as *MMP14*. It is therefore tempting to speculate that this pioneer transcription factor could drive activation of alternative transcriptional programs in VSMCs. The expression of RUNX1 protein in human arteries with intimal thickening is higher compared to those with a healthy morphology, suggesting that RUNX1 may indeed play a role in vascular changes predisposing to disease.

We show that TIMP1, a target of transcriptional re-wiring, promote establishment of clonal VSMC proliferation itself, suggesting a positive feedback loop which might impact vascular disease susceptibility. TIMP1 also stimulates proliferation in hVSMC cultures^34^ and higher TIMP1 serum levels have been associated with elevated cardiovascular disease risk in humans^31^. Future studies are needed to assess whether increased TIMP1 in serum is a cause or consequence of activated VSMCs within diseased arteries. In experimental atherosclerosis, *Apoe*-/-, *Timp1*-/- animals had smaller plaques with reduced VSMC content compared to *Apoe-/-* controls^30,46^. In contrast, femoral artery injury yielded larger neointimal lesions and increased MMP activity in *Timp1*-/- animals^47^. As VSMC lineage tracing was not performed, the impact on VSMC proliferation was not assessed in these studies. Interpreting the effects of global TIMP1 manipulation on VSMC function is further complicated by, firstly, the dual effect of TIMP1 on MMP activity and cell signalling, and, secondly, the ability of TIMP1 signalling to impact multiple cell types in vascular disease^34^. Whereas TIMP1 depends on AKT in macrophages^34^, CD74 signalling via STAT3 is required for TIMP1-stimulation of VSMC clonal proliferation *in vitro* and this axis is also active *in vivo*.

The regulatory interactions observed here are consistent with gene expression changes in atherosclerosis and include factors impacting on cultured VSMCs and VSMC-derived plaque cells. For example, enriched motifs in scATAC-seq analysis of human CAD patients include AP-1, RUNX, CEBP, and also highlighted importance of STAT3 in human vascular disease^2^. Similarly, AP-1 and STAT motifs were highlighted by analysis of lineage-traced VSMCs that adopt a more fibroblastic phenotype during mouse atherogenesis^1^. We also detected RUNX1 and CD74 within αSMA+ cells in developed human atherosclerotic lesions. However, whether enrichment of these motifs in atherosclerosis is caused by ongoing VSMC activation in disease, or implies that these factors also function at later disease states remains to be tested.

Interestingly, studies in zebrafish heart regeneration showed that RUNX1 promotes expression of smooth muscle cell genes in mesenchymal cells^45^, suggesting that in addition to regulating VSMC proliferation, it could also impact fibrous cap formation. The potential links between mechanisms regulating VSMC proliferation and VSMC-derived atherosclerotic plaque cell phenotypes are an exciting topic for future research.

## Methods

Detailed methods are available in the Supplementary information, antibodies are listed in Supplementary Table 4 and primer sequences in Supplementary Table 5.

### Animals and procedures

All experiments were done according to UK Home Office regulations (project licences P452C9545, PP7513347) and were approved by the Cambridge Animal Welfare and Ethical Review Body. Mice (C57Bl/6) were housed on a 12h dark/light cycle, at 19-21°C and 45-65% humidity. Alleles were previously generated; Myh11-CreERt2 (RRID:IMSR_JAX:019079) is a Y-linked transgene that confers expression of a tamoxifen-inducible Cre recombinase in smooth muscle cells, Rosa26-Confetti (RRID:IMSR_JAX:013731) and Rosa26-EYFP (RRID:IMSR_JAX:006148) are Cre-recombination reporter alleles, KI67-RFP (RRID:IMSR_JAX:029802) is an insertion in the Mki67 locus resulting in expression of a KI67-RFP fusion protein and the mutant Apoe allele (RRID:IMSR_JAX:002052) sensitizes mice to high fat diet (HFD)-induced atherosclerosis. VSMC lineage-labelling was achieved by administration of tamoxifen (10 injections of 1 mg/ml in intraperitoneal injections over two weeks). Note that Confetti-GFP induction occurs at low frequency^4^. Myh11-CreERt2 is Y-linked, so males were used for VSMC lineage-tracing. Animals were rested for >1 week after the last injection to allow tamoxifen washout before tissue harvest, high fat diet (HFD) feeding (21% fat and 0.2% cholesterol, Special Diets Services), vascular injury, or randomized into groups receiving intraperitoneal injections of recombinant murine TIMP1 protein (200 µg/kg, n=5) or vehicle (phosphate buffered saline, PBS, n=4) daily for 9 days at the end of a 4 week HFD protocol. The left carotid artery was tied off with a silk suture just under the bifurcation point and analysed 5-28 days after surgery after pre-operative analgesic subcutaneously (~0.1 mg/kg body weight, Buprenorphine) under isoflurane anaesthesia (inhalation, 2.5-3%, 1.5 L/minute induction, maintained at 1.5%). Animals were euthanized (cervical dislocation or CO2 asphyxiation) and perfused with cold PBS before tissue removal.

### Human tissue

Human arteries were collected after informed consent and approval by the Cambridgeshire 1 or East of England - Cambridge South Research Ethics Committee (REC ref 15/EE/1052, H00/514). Non-plaque aorta was obtained from organ donors via the Cambridge Collaborative Biorepository for Translational Medicine. Carotid endartectomy samples were obtained from the Royal Papworth Hospital research tissue bank. Cell isolates from a total of 12 donors (age and sex; 27M, 20M, 70M, 75F, 45F, 65M, 62F, 51M, 68F, 69M, 72F, 68M, 61M) and histological analysis of 9 carotid artery samples (72F, 82M, 80F, 67M, 67F, 58F, 74M, 54M and 50F) and 10 aorta (60M, 24M, 84F, 65M, 49M, 35M, 41M, 75F, 18M, 59F) were included. Both male and female tissue and cell isolates were included in all analyses. The experiments with human samples conform to the principles outlined in the Declaration of Helsinki.

### VSMC isolation, culture and treatment

Human VSMCs (hVSMCs) were cultured from aortas of patients undergoing cardiac transplant or aortic valve replacement in hVSMC-specific medium (Promocell, SMC-GM2, C22062) supplemented with antibiotics. Human VSMCs were serum starved in media supplemented with 0.1% BSA prior to TIMP1 treatment. Single-cell suspensions of mouse VSMCs (mVSMCs) were generated from freshly isolated aortas of wild type or VSMC lineage-labelled animals (Myh11-Confetti or Myh11-EYFP) and cultured in DMEM supplemented with 10% FCS and antibiotics (complete medium).

RUNX1 overexpression lentiviral vector were generated by inserting full-length mCherry and Runx1 cDNA (*Runx1-202*) linked by T2A into pLentiGFP backbone (Addgene, cat no. 17448). VSMCs were transduced with lentivirus in media containing 10 µg/ml protamine sulphate (Sigma, P3369). Transfection with siRNA (50 nM ON-TARGETplus® SMART Pool, Dharmacon, targeting human *RUNX1* (L-003926-00-0005), murine *Runx1* (L-048982-00-0005), murine *Stat3*, L-040794-01-0005 or non-targeting control siRNA, Control Pool, D-001810-10-05) was done using Lipofectamine RNAiMAX transfection reagent (13778030, Invitrogen).

Cells were treated with recombinant (r) murine (m) or human (h) protein, peptide or small molecule inhibitors as detailed in figure legends and Supplementary Table 3. Transfection with siRNA was performed the day before TIMP1 addition and cells were pre-treated for 1 hour (CD74 antibody, GM6001, STAT3i, AKTi, P38i1, P38i2, P38 control i) or 6 hours (CD74 peptide) prior to TIMP1 treatment.

### Clonal VSMC proliferation assay

Cells (EYFP+ VSMCs from aortas of Myh11-EYFP isolated by flow cytometry-assisted cell sorting or medial cells from Myh11-Confetti animals mixed with medial cells from wild type animals in a 1:3 ratio) were seeded in 96-well imaging plates (5,000 cells/well, CellCarrier-96 Ultra, Perkin Elmer) in complete medium. Low titre lentiviral transduction with RUNX1 and control virus was performed 3 days post seeding or cells were treated as indicated in complete medium from day four after seeding. Medium with fresh reagents was added twice weekly. Cells were imaged 4, 7, 14, and 21 days after plating using an Opera Phenix high content screening system (Perkin Elmer). Image analysis was done using Harmony software v5 (Perkin Elmer) and quantification was performed in Fiji v2.15.1. Patches were defined as three or more contiguous EYFP+RFP+ (RUNX1 experiment) or same-colour lineage-traced Confetti+ cells (TIMP1 assays).

### ATAC-seq

Single-cell suspensions of carotid arteries from 4-5 lineage-labelled Myh11-EYFP/Mki67-RFP animals (11 weeks old) were generated 7 days after carotid ligation surgery, stained for SCA1 and EYFP+SCA1+ or EYFP+SCA1- cells isolated by cell sorting (BD FACSAria™ III, BD Bioscience). In parallel, lineage-traced (EYFP+) VSMCs were isolated from non-ligated carotid arteries of a non-ligated littermate. The Omni-ATAC protocol^48^ was used to process 5,000 cells from each sample type in parallel (two independent replicates generated on separate days), using 10-13 PCR amplification cycles and libraries sequenced with a 50 bp paired-end run cycle (Illumina HiSeq or HiSeq2500-RapidRun, 40-60 million reads/sample).

Details of ATAC-seq data analysis is provided in the Supplementary information. Condition-specific peak lists include peaks overlapping at least 50% between biological replicates, and the pan-VSMC peak list is the union of condition-specific lists. Peaks were associated with genomic features using ChIPseeker v.1.24.0. Differential accessibility was scored in LIMMA (SeqMonk v.1.47.2) using a >2-fold change threshold and Benjamini-Hochberg adjusted p-value <0.01. Peaks were annotated to genes and gene ontology (GO) term enrichment analysis performed using GREAT v.4.0.4^49^ (http://great.stanford.edu/public/html/index.php) with default settings and all genes as a background. For the SCA1+ condition, the top 4000 peaks ranked by fold-change were used. Motif enrichment analysis was done on 500 bp genomic sequences centred on ATAC-seq peak summits using MEME-ChIP v.5.4.1^50^ for differentially accessible peaks using HOCOMOCO mouse v.11.

### Gene regulatory network analysis

Single-cell RNA sequencing (scRNA-seq) profiles of VSMCs isolated from mouse carotid arteries 5 days after carotid ligation surgery (GSE162167) and the bulk ATAC-seq data generated here (accession number below) were used for gene regulatory network (GRN) modelling using CellOracle v.0.10.5^24^. A "BaseGRN", including a list of all potential TF-target gene interactions, was constructed using pan-VSMC ATAC-seq peaks. Transcriptional start site (TSS) annotation and motif scan were performed with this union peak data using the default settings of CellOracle. Cells from the day 5 carotid ligation injury scRNA-seq data, clustered as described^20^, were annotated with VSMC states (cluster 9 as CYC, clusters 4 and 3 as PrP, cluster 2 as LNK, clusters 0, 5, 7, 8 as Non-RSP, cluster 1 as stress and clusters 6,10 as path 2 (Figure 2a, Extended Data Fig. 2a)) and processed using Scanpy v.1.9.1^51^. For GRN modelling, the top 4000 highly variable genes were supplemented with transcription factors showing pseudotime-dependent expression (*Eno1, Id3, Mef2c, Hif1a, Nfia, Hmga1, Ebf1, Rora, Klf9, Jund, Foxp1, Thra, Prrx1, Nfix*), and *Stat3, Twist1*, and *Runx2*.

GRNs were constructed with the top 2,000 interactions for all VSMC states. Topological analyses of GRNs were performed with CellOracle and GRNs were visualized with Cytoscape v.3.7.2^52^. GRNs for the stress and path2 states (coloured in grey in Fig. 2a) were not investigated further. Venn and Euler diagrams were created by using CRAN R package eulerr v.7.0.0. Gene Ontology (GO) term analysis and visualization were performed with BiNGO v.3.0.4 and GOlorize v.1.0.0.beta1 with the default settings, hypergeometric test, Benjamini-Hochberg False Discovery Rate correction, significance level of 0.05. Communities were detected using clusterMaker2 v.1.3.1^53^ with the MCODE algorithm^54^(fluff option on).

A rewiring score was calculated for each node as the sum of absolute change in connectivity score between the PrP *vs*. Non-RSP networks in R v.4.2.2 using igraph v.1.4.2 (https://igraph.org) functions to obtain adjacency matrices. Differential gene expression values for cells in PrP *vs*. Non-RSP cell states were calculated using Wilcoxon rank sum-test (with Bonferroni correction as the method for multiple testing correction) and gene expression values were plotted using Seurat v.4.3.0 ^55^ based on sctransform-normalized values^20^.

*In silico* simulations were performed with the default settings and top 10,000 interactions in all GRNs with an expression value of zero for the knockout simulations or of 1.5 times the maximum gene expression value for the overexpression simulations. Systematic knockout and overexpression simulations were performed for all TFs present in the GRNs with the same parameters along the proliferation associated trajectory including cells from Non-RSP, LNK, PrP, and CYC populations. TFs were scored based on sum of positive or negative perturbation scores (PS) which were calculated as the inner product of developmental flow and simulation vectors as described^24^. Root cell selection for pseudotime calculation was based on *Myh11* expression and cell clustering.

Prior to GRN modelling with post-carotid ligation day 7 dataset, this data was integrated with the day 5 dataset with sctransform-based normalization using Seurat v.4.3.0^55^ and further clustered (24 principal components, and 1.9 resolution) in order to identify cell clusters corresponding to equivalent cell states. This information was then used for the GRN modelling.

### Analysis of published scRNA-seq datasets

Filtered scRNA-seq profiles (GSE155513) of VSMC-lineage labelled cells from murine atherosclerotic arteries from 3 time points (8, 16, and 22 weeks) were integrated with sctransform-based normalization (Seurat v.4.3.0)^55^ and clustered (20 principal components at 0.3 resolution). Differential expression testing for each cell cluster *vs*. all other VSMCs, or for modulated (clusters 3 and 0) *vs*. contractile (cluster 1) cells was done with the Wilcoxon rank-sum test (p-adj<0.05, Bonferroni-corrected).

Gene set signatures were assessed using UCell v.2.2.0^56^. PrP state signature genes include those showing differential expression between PrP and Non-RSP cells (logFC>0.5, p-adj<0.05, Supplementary Table 1e). "Positive regulator" signatures were defined as TFs with top 10 highest positive PS for overexpression simulation or negative PS for knockout simulation + the target genes of these TFs in the PrP GRN (edge connectivity>0.1). "Negative regulators" signatures contained TFs with top 10 highest negative PS for overexpression simulation or positive PS for knockout simulation + the target genes of these TFs in the PrP GRN (edge connectivity>0.1).

ScRNA-seq profiles (GSE155512) of human atherosclerotic carotid plaques^13^ were filtered to include only cells minimum 200 and maximal 4,000 genes, total counts >500, and mitochondrial reads <10% and data from three different patients integrated. Data integration and clustering was repeated after subsetting VSMCs (17 principal components, Seurat v.4.3.0)^55^. Differential expression testing was performed for each cell cluster against all other VSMCs as above, with LogFC>0.25 threshold. Heatmaps were created with the CRAN R package pheatmap v.1.0.12 with complete linkage and correlation as the distance metric.

### Colocalisation analysis

Colocalisation analyses were done for human orthologues of genes ranking in the top 50 for rewiring score or top 10 for positive or negative perturbation score, plus factors associated with TIMP1 signalling (STAT3, CD74). Summary statistics for CAD GWAS^57^ were converted from hg19 to hg38 build with CrossMap^58^ and colocalized with VSMC cis-eQTL from smooth muscle cells isolated from human umbilical cord (n=1499)^59^ and human aortic smooth muscle cell (HAoSMC) cis-eQTL for quiescent (n=139) and proliferative (n=145) cells^60^. For eCAVIAR, variants with p<0.001 within 1mb of the top cis-eQTL association for each gene were overlapped with corresponding GWAS variants (p<0.001) in the same locus. eCAVIAR colocalization test was performed for genes that had 5 or more overlapping variants with GWAS summary data. Linkage between overlapping variants was estimated from individuals of European ancestry in the 1000 Genomes Phase 3 using PLINK^61,62^. Summary-based Mendelian Randomization (SMR)-based analysis was done with SMR v1.3.1^63^ with default settings and peqtl-smr set to 0.001. European samples in the 1000 Genomes project served as reference for linkage disequilibrium estimation. Colocalization events with eCAVIAR colocalization posterior probability (CLPP)>0.01 or p_SMR<0.05 were considered significant.

### Bulk gene expression

RNA was extracted using a RNeasy Mini kit (Qiagen, 74104) and cDNA generated using QuantiTect Reverse Transcriptase (Qiagen, 205311). Bulk RNA-seq was conducted on RNA isolated from of six different hVSMC isolates following 3 hours serum starvation in serum free media containing 0.1% BSA, followed by 6 hours TIMP1 (or vehicle) treatment in serum free media containing 0.1% BSA. Libraries were prepared from oligo-dT-purified mRNA and sequenced (150 bp paired-end reads, Illumina Novaseq 6000). Raw data reads were trimmed with Trim Galore v.0.6.7, and aligned to the human genome (GRCh38) with Kallisto v.0.46.2. Gene set enrichment analysis (GSEA)^64^ was performed after Trimmed Mean of M-values (TMM) normalization. Quantitative real-time PCR was performed using SsoAdvanced Universal SYBR Green Supermix (Biorad, 1725270).

### Protein detection

Whole cell protein lysates were prepared in RIPA buffer freshly supplemented with proteinase inhibitors (Millipore) and phosphatase inhibitors (Millipore) and separated on gradient (4-12%) polyacrylamide gels. Protein concentration was determined using the BCA method (23227, Pierce BCA protein assay kit, Thermo Fisher). Primary antibodies were detected by HRP-labelled secondary antibodies using chemiluminescence detection (Amersham ECL detection reagent, GE Healthcare) and exposure to photographic film. Phosphokinase array (R&D Systems, ARY003C) was incubated with cell lysates according to manufacturer's instructions. Densitometry values of spots were calculated in ImageJ and expressed as arbitrary units.

### Immunostaining, EdU incorporation and imaging

Cryosections (14 µm) of ligated left carotid arteries from lineage-labelled Myh11-Confetti animals and plaque containing arteries from Myh11-Confetti/Apoe animals after high fat feeding were permeabilized, stained and mounting in RapiClear 1.52 (Sunjin Lab). Confocal imaging was done with a Leica SP8 scanning laser microscope (Leica) using a 20x lens and image analysis done in Imaris v9.2.

Human arteries were formaldehyde-fixed and paraffin-embedded (FFPE) and sections (4 µm) were dewaxed, processed for antigen retrieval and sequential sections were either H&E stained, or co-stained for αSMA and either RUNX1, TIMP1, or CD74.

Cells cultured in 96-well plates and treated as indicated in figure legends were fixed and stained. Cells seeded in 96-well imaging plates (10,000 per well) were incubated with EdU (10 µM) for 16 hours and EdU incorporation was detected using the Click-iT EdU kit (C10340, Thermo Fisher Scientific), stained with DAPI for 10 minutes. Cells were imaged using an Opera Phenix high content screening system, with a x20 water objective (Perkin Elmer). Image analysis was done using Harmony software (Perkin Elmer), quantification based on intensity values with nuclei defined by DAPI staining, and for mouse VSMCs from Myh11-EYFP animals, within cell borders (EYFP). Quantification of EdU+ cells was done by thresholding intensity values measured in detected nuclei.

### Chromatin immunoprecipitation (ChIP)

ChIP was performed on 3x10^6^ hVSMCs using a SimpleChIP kit according to the manufacturer's instructions (Cell Signaling Technology, 56383). Cells were crosslinked with formaldehyde (1% final concentration, 15 minutes). Chromatin (5 μg) sheared using a Diagenode bioruptor (15 pulses of 30 seconds with a 30 second rest) was immunoprecipitated with 10 μl STAT3 antibody (Clone D3Z2G, Cell Signaling Technology, 12640) or an equal amount of control IgG (Cell Signaling Technology, 2729). Antibody-bound protein/DNA complexes were captured using Protein-G coated magnetic beads, reversed cross-linked and digested with proteinase K. DNA was purified and quantified using qPCR.

### Flow cytometry

Single-cell suspensions were incubated with 5 µg/mL TruStain FcX anti-mouse CD16/32 antibody (Biolegend) in FACS buffer (0.5% (w/v) BSA in PBS) for 15 minutes on ice, stained with primary antibody for 30 minutes at room temperature and washed twice (FACS buffer). This was followed by incubation with secondary antibody (in FACS buffer, 30 minutes at room temperature) and washed twice (FACS buffer) for non-conjugated antibodies. Intracellular targets were stained using the Foxp3 staining buffer set (eBioscience). Cells were analysed using an Imagestream system (Amnis® ImageStream®XMk II, Luminex) using IDEAS v6.2, or sorted (BD FACSAria™ III, BD Bioscience). Gating was done using cells stained with control IgG or non-expressing cell populations.

### RNA scope

RNA *in situ* hybridisation was performed using RNA Scope® Multiplex Fluorescent v2 kits, according to the manufacturer’s instructions (ACD). Experiments used Hs-TIMP1-C2 coupled to opal 570, Hs-CD74-C3 coupled to opal 620 and Hs-ACTA2-C1 coupled to opal 690 (ACD). Imaging was done using a ZEISS Axioscan slide scanner. Analysis was performed in QuPath using the subcellular detection feature.

### Statistical analysis

Group size and replicate numbers were decided following power calculations based on observed or expected variation using a power of 90% and a 0.05% type I error rate. Where technical repeats were conducted of the same cell line/animal, these have been averaged and biological repeats used for statistical analysis. Data visualisation and analysis were conducted using R, the IGV browser and GraphPad Prism V8. Statistical testing information is provided in Supplementary Table 6. Data meeting normality (p>0.05, Shapiro-Wilk) and equal variance criteria were subjected to two-sided Student’s t-tests (with F-tests for equal variance) or ANOVA (Brown forsythe and bartlett testing for equal variances), unless otherwise stated. When these assumptions were not met, appropriate non-parametric tests were chosen. ANOVA was followed by post hoc testing (Dunnett's for comparisons against control, Tukey's for all pairwise comparisons) to calculate multiplicity-adjusted p-values. For the analysis of clonal patch formation over time, generalised linear modelling (Poisson regression) was employed. Model suitability was assessed by ensuring residual deviance was smaller than degrees of freedom and checking for over-dispersion, where the variance of the response variable is not greater than the mean as assumed by the Poisson distribution. Diagnostic checks, including examination of deviance residuals and consideration of alternative models, were performed to assess goodness-of-fit and model adequacy. Clonal patch sizes were analysed using linear modelling. In cases where data were not normally distributed, log transformation was applied to achieve normality. Normality of residuals was assessed using QQ plotting and the Shapiro-Wilk test. Differential expression and accessibility analyses were done using defaults tests in LIMMA (2-sided modified *t*-test, Benjamini-Hochberg correction for multiple testing) and Seurat v.4.3.0 (2-sided non-parametric Wilcoxon rank-sum test, Bonferroni correction). UCell is based on Mann-Whitney U (MWU) statistic.

## Extended Data

**Figure:**
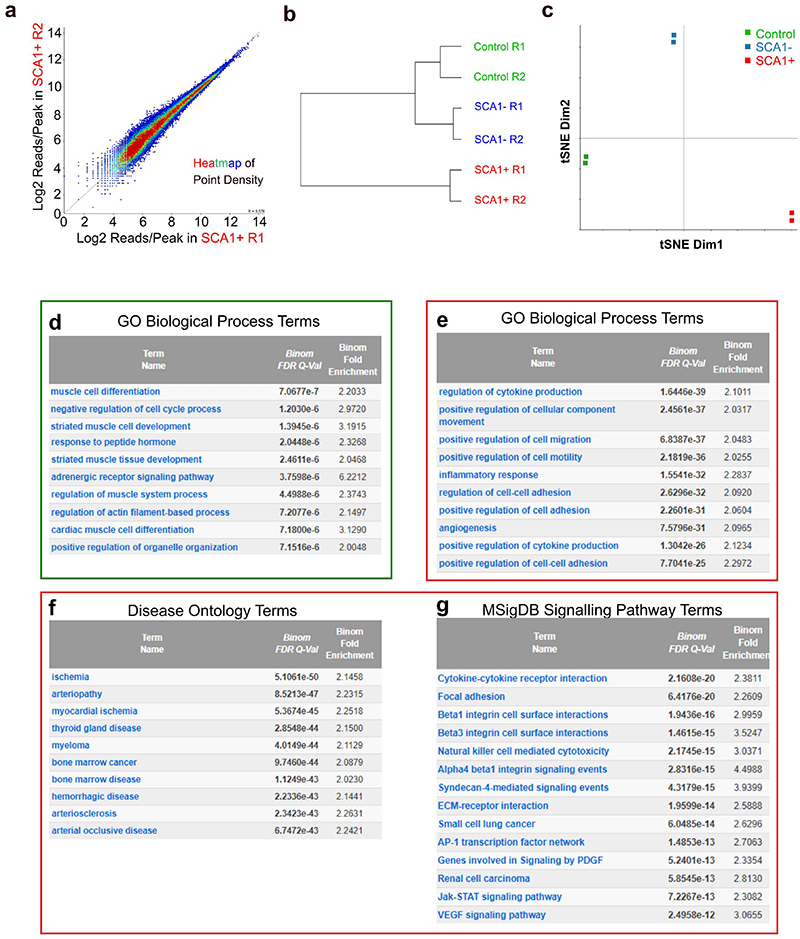


**Figure:**
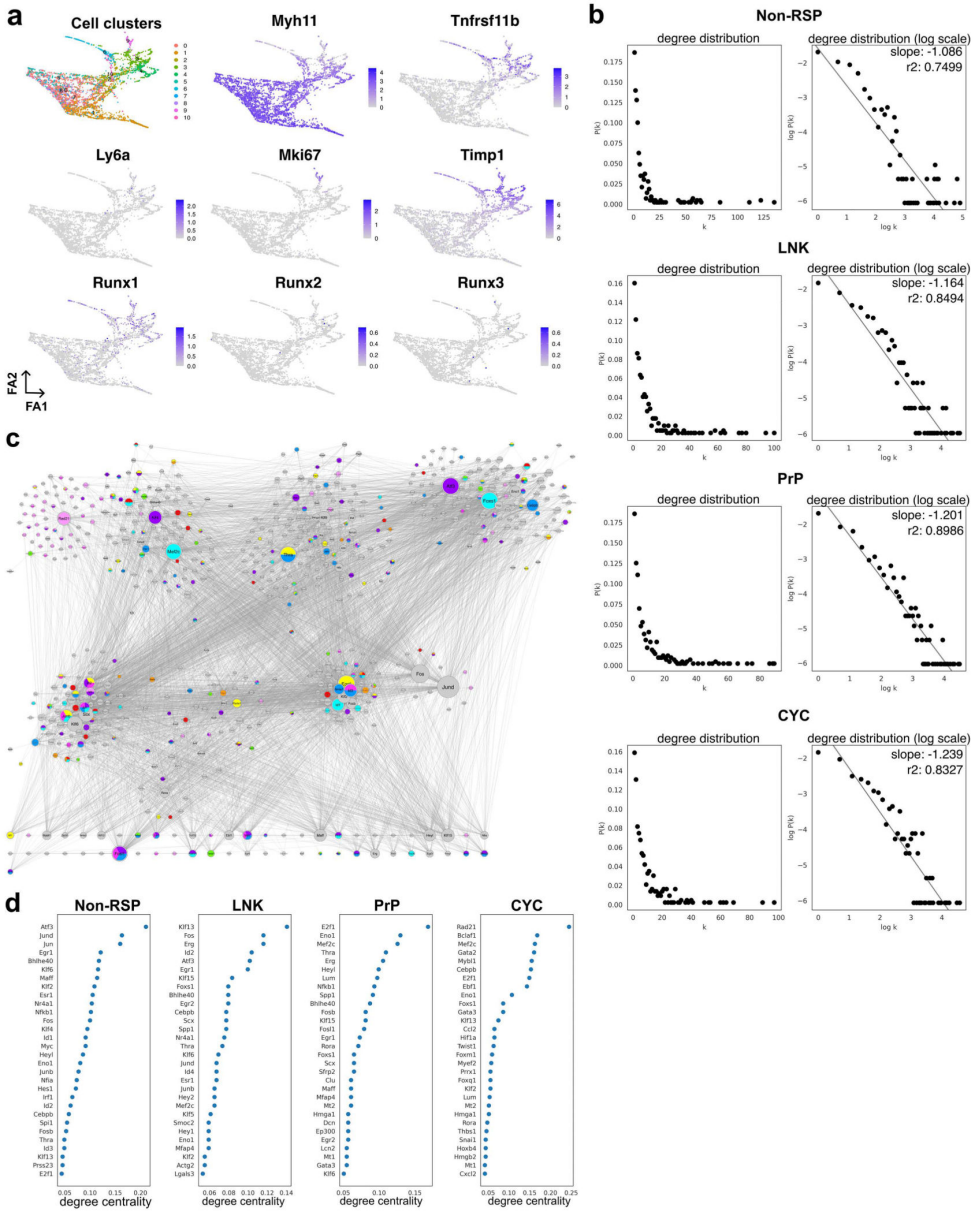


**Figure:**
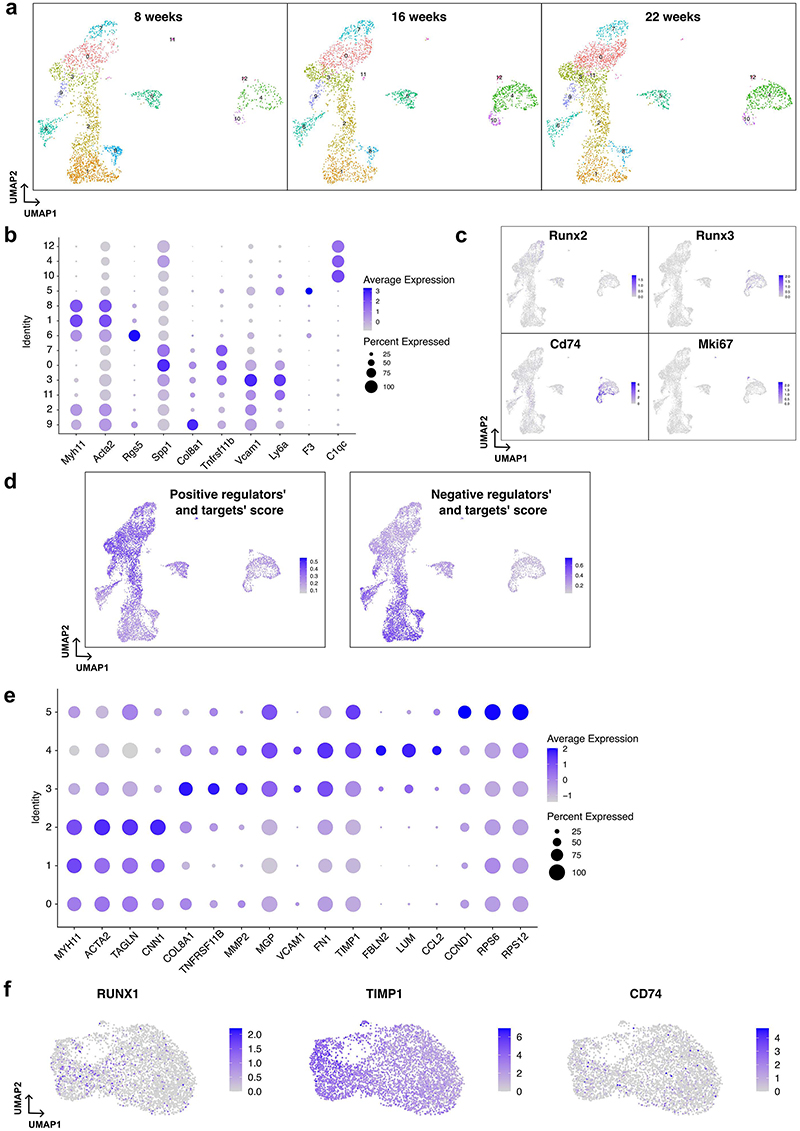


**Figure:**
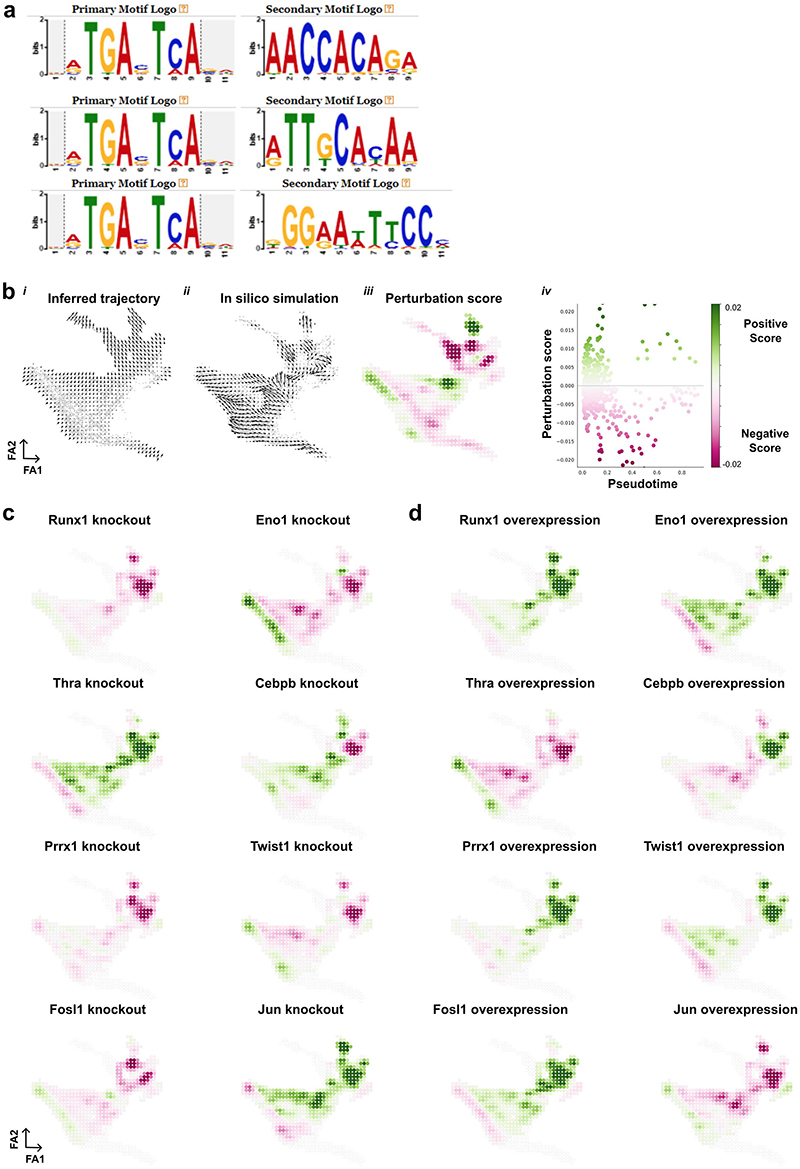


**Figure:**
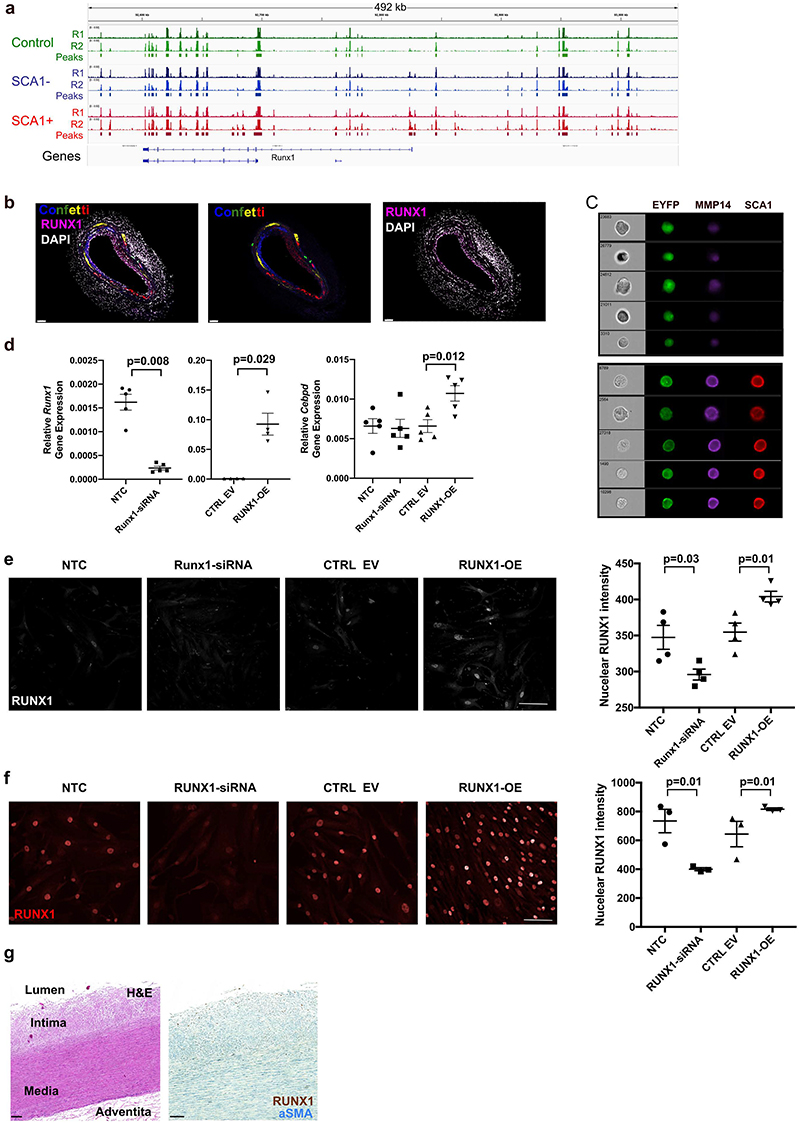


**Figure:**
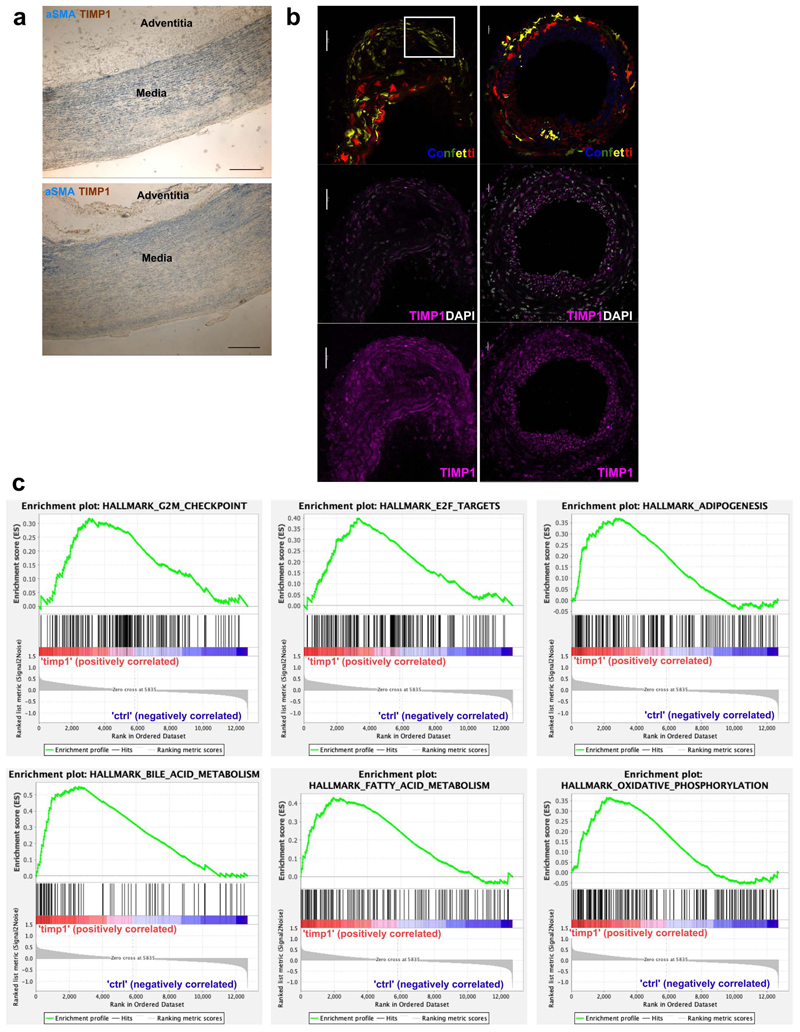


**Figure:**
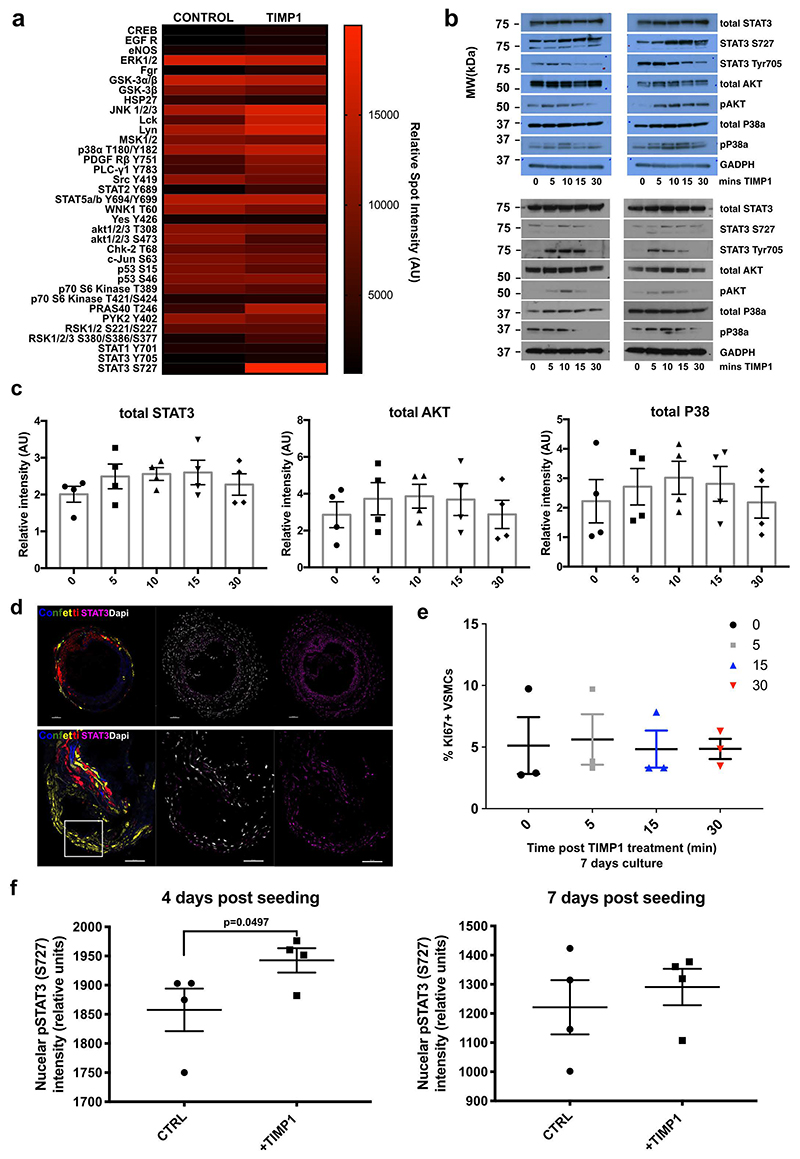


**Figure:**
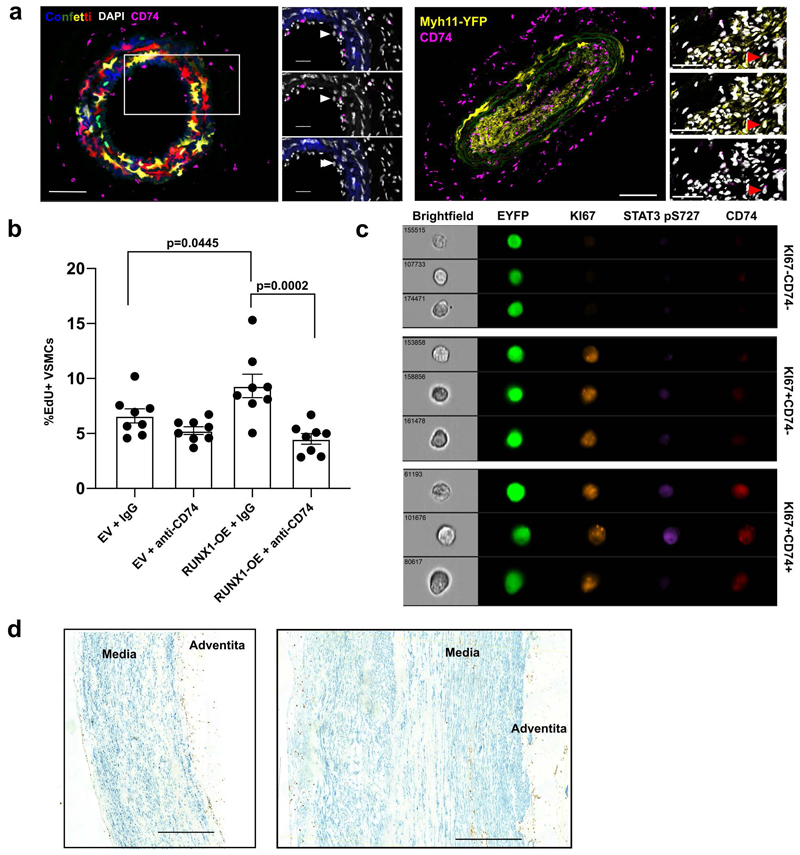


## Supplementary Material

Supplemental Material

## Data Availability

The ATAC-seq datasets (GSE246646) and bulk RNA-seq data from TIMP1-treated hVSMCs and controls (GSE246647) have been deposited to the NCBI gene expression omnibus (GEO). ScRNA-seq datasets of VSMCs from mouse arteries after injury (GSE162167^20^), murine atherosclerosis (GSE155513^13^) and human carotid plaque cells (GSE155512^13^) are available from GEO. CAD GWAS^57^ was downloaded from the GWAS Catalog. The cis-eQTL data are VSMC cis-eQTL from smooth muscle cells isolated from human umbilical cord (n = 1499)^59^ and human aortic smooth muscle cell (HAoSMC) cis-eQTL for quiescent (n = 139) and proliferative (n = 145) cells^60^. Source data is provided as Supplementary information.

## Code availability

Data analysis was done using available packages, as listed in the methods section. Scripts used for GRN inference, rewiring calculation and analysis of published scRNA-seq datasets are available on GitHub (https://github.com/hfj22/Lambert_Oc_et_al_2024_NCVR).
