# Supplementary material for: Network-based prioritization and validation of regulators of vascular smooth muscle cell proliferation in disease
Source: Nat Cardiovasc Res. 2024 Jun 6;3(6):714–33. doi: 10.1038/s44161-024-00474-4 (PMC11182749; doi:10.1038/s44161-024-00474-4)

Source data for Extended Figure 2d

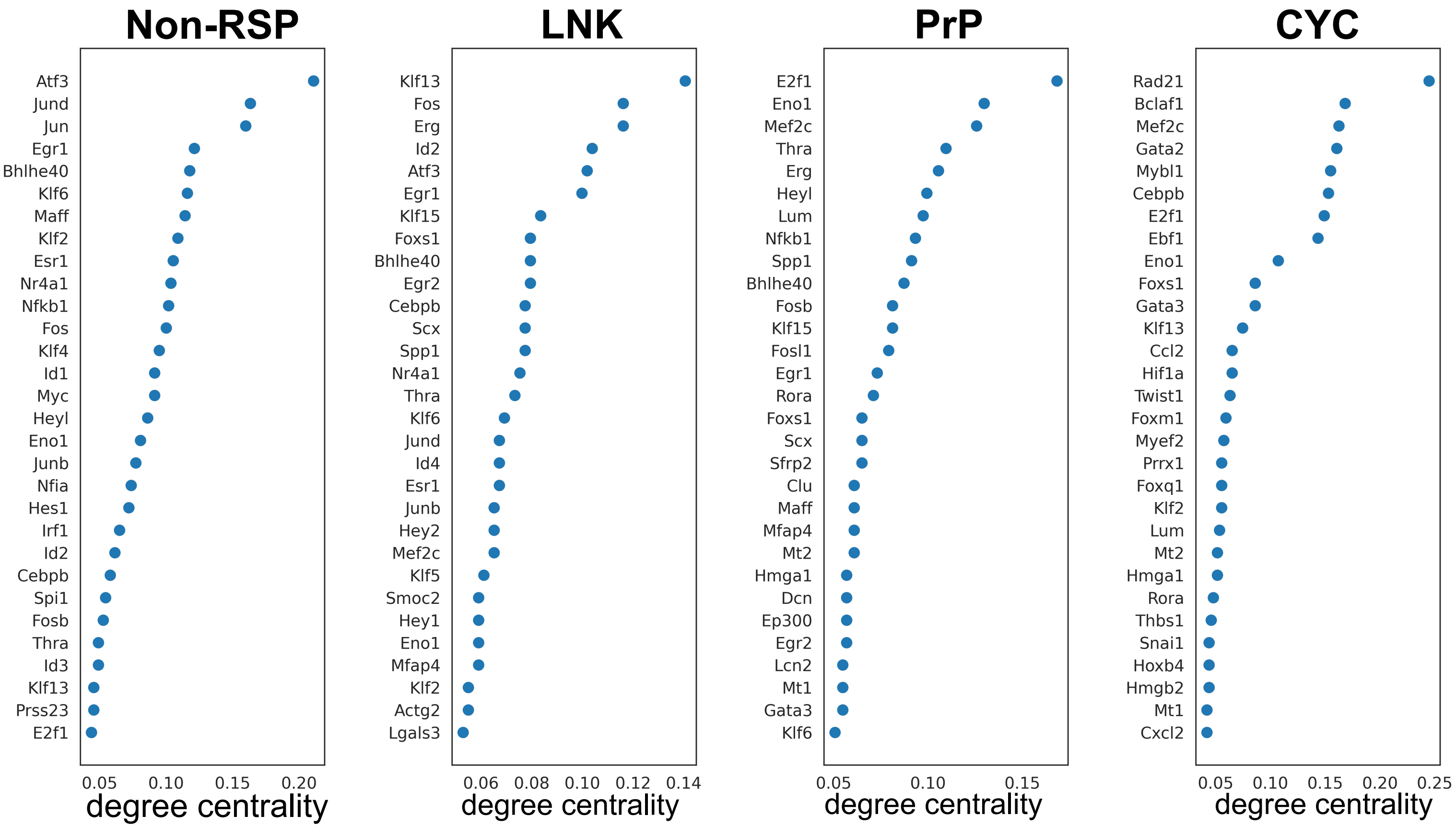

Supplement: Supplementary file 1 [file 44161_2024_474_MOESM14_ESM.pdf]
