# Supplementary material for: Network-based prioritization and validation of regulators of vascular smooth muscle cell proliferation in disease
Source: Nat Cardiovasc Res. 2024 Jun 6;3(6):714–33. doi: 10.1038/s44161-024-00474-4 (PMC11182749; doi:10.1038/s44161-024-00474-4)

nature cardiovascular research

Supplementary information https://doi.org/10.1038/s44161-024-00474-4

# Network-based prioritization and validation of regulators of vascular smooth muscle cell proliferation in disease

In the format provided by the authors and unedited

**Supplementary information**

**Content**

Supplementary Discussion

Supplementary Methods

Supplementary References

Supplementary Figs. 1-3: Gating strategy for Figs. 1, 6d, 8f-h and Extended Data Fig. 8c

Supplementary Figs. 4-6: Enlarged views of Figs. 2d-f, 3c and f and Extended Data Fig. 2d

**Supplementary Discussion**

One potential constraint of GRN analyses is the limitation to variably expressed genes. For example, neither the master VSMC regulator myocardin nor its partner SRF show differential expression across the VSMC population at this early timepoint post injury. However, while the motif enrichment analysis in differentially accessible ATAC-seq peaks identified the SRF motif, little change in open chromatin regions at contractile genes was observed, possibly reflecting that epigenetic modification at these SRF targets are not key to the initial VSMC state changes.

**Supplementary Methods**

***Animal lines***

| Name | Alleles | Description |
|---|---|---|
| Myh11-EYFP | Myh11-CreERt2, Rosa26-EYFP | VSMC-specific expression of EYFP after tamoxifen treatment.<br>Used for VSMC lineage-labelling. |
| Myh11-Confetti | Myh11-CreERt2, Rosa26-Confetti | VSMC-specific expression of GFP, RFP, YFP or CFP in a stochastic manner after tamoxifen treatment.<br>Used for multi-colour VSMC lineage-labelling. |
| Myh11-EYFP/Mki67-RFP | Myh11-CreERt2, Rosa26-EYFP, KI67-RFP | VSMC-specific expression of EYFP after tamoxifen treatment. Expresses RFP in KI67+ cells (not used for identification in this study)<br>Used for VSMC lineage-labelling. |
| Myh11-EYFP/Apoe | Myh11-CreERt2, Rosa26-EYFP, Apoe-/- | VSMC-specific expression of EYFP after tamoxifen treatment. Develops atherosclerosis after high fat diet feeding.<br>Used for VSMC lineage-labelling in experimental atherosclerosis. |
| Myh11-Confetti/Apoe | Myh11-CreERt2, Rosa26-Confetti, Apoe-/- | VSMC-specific expression of GFP, RFP, YFP or CFP in a stochastic manner after tamoxifen treatment. Develops atherosclerosis after high fat diet feeding.<br>Used for multi-colour VSMC lineage-labelling in experimental atherosclerosis. |

### *VSMC isolation, culture and treatment*

Human VSMCs (hVSMCs) were from aortas of patients undergoing cardiac transplant or aortic valve replacement. After manually removing the endothelial layer and adventitia, the medial layer was cut into 2-3 mm² pieces, placed into 6-well plates containing 1 ml media (DMEM supplemented with 20% fetal calf serum (FCS),100 U/ml penicillin, 100 μg/ml streptomycin) and cultured for 1-2 weeks to allow cells to migrate out of the tissue. After establishment, cells were cultured in hVSMC-specific medium (Promocell, SMC-GM2, C22062) supplemented with 100U/ml penicillin, 100μg/ml streptomycin and were studied at passages 2–10.

Single cell suspensions of mouse VSMCs (mVSMCs) were generated from freshly isolated aortas of wild type or VSMC lineage-labelled animals (Myh11-Confetti or Myh11-EYFP). Aortas were cleaned off connective tissue, cut open longitudinally and the endothelium removed by gentle scraping with a cotton bud before removal of the adventitia after brief enzymatic digestion. The medial layer was digested to a single-cell suspension in DMEM supplemented with 2.5 mg/mL Collagenase IV (Invitrogen) and 2.5 U/mL Elastase (Worthington) at 37℃. Cells were cultured in DMEM supplemented with 10% FCS, 100U/ml penicillin, 100μg/ml streptomycin (complete media).

RUNX1 overexpression was achieved by lentiviral transduction. Gibson assembly was used to insert full length mCherry and Runx1 cDNA (*Runx1-202*) linked by T2A into pLentiGFP backbone (Addgene, cat no. 17448). The resulting pLenti-mCherry-Runx1 (or plx302 vector encoding RFP only, empty vector, EV) were co-transfected with third generation lentiviral plasmids (pRRE, pRSV-Rev and pMD2.G) into HEK293FT cells using Trans-iT-LT1 transfection reagent (Mirus MIR2300). Lentivirus-containing medium collected after 48h and 72h after transfection was pooled and concentrated using Lenti-X concentrator (631232, Takara). VSMCs were transduced with lentivirus in media containing 10 μg/ml protamine sulphate (Sigma, P3369).

### *Clonal VSMC proliferation assay (detailed)*

To measure the effect of RUNX1 overexpression on VSMC clonal proliferation, single cell suspensions of medial cells from VSMC-lineage labelled Myh11-EYFP animals were generated, EYFP+ VSMCs isolated by flow cytometry-assisted cell sorting and seeded at a density of 5,000 cells per well of a 96-well imaging plate (CellCarrier-96 Ultra, Perkin Elmer) in DMEM supplemented with 10% (v/v) FBS, 100 U/mL penicillin, 100 mg/mL streptomycin. Low titre lentiviral transduction was performed 3 days post seeding and media was changed twice weekly. For TIMP1 clonal proliferation assays, medial cells from VSMC-lineage labelled Myh11-Confetti animals were mixed with medial cells from wild type animals in a 1:3 ratio and a total of 5,000 cells seeded per well of a 96-well imaging plate (CellCarrier-96 Ultra, Perkin Elmer). Cells were treated as indicated in complete medium from day four after seeding, and medium with fresh reagents was added twice weekly. Cells were imaged 4, 7, 14, and 21 days after plating using an Opera Phenix high content screening system (Perkin Elmer). Image analysis was done using Harmony software (Perkin Elmer) and quantification was performed in Fiji. Patches were defined as an area with three or more contiguous EYFP+RFP+ cells for the RUNX1 experiment and three or more contiguous lineage-labelled cells of the same colour in the TIMP1 assays. A mask was generated using thresholding after enhancing local contrast and to calculate patch area.

### *Recombinant TIMP1 protein purification*

Recombinant (r) TIMP1 proteins were produced and purified in an endotoxin-free HEK293F cell system (Thermo Fisher Scientific Inc.) harboring a eukaryotic (pcDNA3.4) expression plasmid encoding the Igkappa antibody chain secretion sequence followed by TIMP1-encoding cDNA

sequences (full-length TIMP1 and the N-terminal peptide (N-TIMP1) from both mouse and human, referred to as TIMP1 below), as described previously[1].

### *ATAC-seq data analysis (detailed)*

Following trimming of adaptor sequences, reads >20bp were aligned to the GRC38.98 Mus musculus genome (Bowtie2 v.2.3.5). Reads were shifted to account for Tn5 transposase adaptor sequence insertion (+4/-5bp) using alignmentSieve within deeptools v.3.4.3. BAM files were generated using the view command within samtools v.1.18 and deeptools v.3.4.3 was used for conversion to BigWig format for data visualisation in the IGV browser, removing duplicate reads and normalisation by counts per million.

Peak-calling was done after removing reads mapping to a blocklist with repetitive sequences and other mapability issues (a total of 0.02% of the genome, provided in the Supplementary information, samtools, v.1.18) or having low confidence (bedtools v.2.27.1) using MACS2 v.2.2.7.1 (paired-end mode with parameters: –f BAMPE –nomodel –nolambda –q 0.05 –broad). Only peaks that overlapped at least 50% between biological replicates were included in condition-specific peak lists. Peaks were associated with genomic features using ChIPseeker v.1.24.0. A pan-VSMC accessibility list was generated by taking the union of condition-specific peaks. Each dataset was log2 transformed, normalised (to align peaks with the highest levels of accessibility between samples) and differential accessibility scored in LIMMA (SeqMonk v.1.47.2) using a >2-fold change threshold and Benjamini and Hochberg adjusted p-value <0.01.

Motif enrichment analysis was done on 500 bp genomic sequences centred on ATAC-seq peak summits (identified using the refinePeak command in MACS2 v.2.2.7.1), following masking of repetitive elements using RepeatMasker v.4.1.1 (https://www.repeatmasker.org/cgi-bin/WEBRepeatMasker), with default settings using MEME-ChIP v.5.4.1[2] for differentially accessible peaks *vs*. a background of all peaks. HOCOMOCO mouse (v.11) was used as the transcription factor motif database, the algorithm was instructed to search for 5 motifs using a CentriMo threshold ≥7. Motifs that were similar to highly repetitive DNA or a very strong true motif, or called as significantly centrally enriched which disagreed with judgement by eye were discarded as spurious.

### *Heatmaps*

Heatmaps were created with the CRAN R package pheatmap v.1.0.12. Pearson residuals for sctransform-normalised gene expression values were used as the scaled data to create heatmaps for the Day 5 injury dataset. Genes were clustered with complete linkage and correlation as the distance metric along the injury associated cell trajectory. Gene clustering and ordering for Day 5 was used to create heatmap for the visualization of gene expression changes in the human carotid plaque VSMCs with the log-normalized and scaled data.

### *Immunostaining*

Cryosections (14 µm) of ligated left carotid arteries from lineage-labelled Myh11-Confetti animals and plaque containing arteries from Myh11-Confetti/Apoe animals after high fat feeding were permeabilized for 20 minutes in 0.5% (v/v) Triton X-100 (Sigma Aldrich) in PBS. Sections were blocked for 1 hour at room temperature in 1% (w/v) bovine serum albumin and 10% (v/v) of normal goat serum (Dako), and incubated with primary antibody or isotype control, diluted in blocking buffer overnight at 4°C. Following 3x 5 minutes washes in PBS, Alexa Fluor 647-conjugated secondary antibodies were added for 1 hour at room temperature, sections were washed 3x 5 minutes in PBS and nuclei stained with DAPI (1 µg/mL in PBS, 10 minutes at room temperature) before rinsing in PBS and mounting in RapiClear 1.52 (Sunjin Lab). Confocal imaging was done with a Leica SP8 scanning laser microscope (Leica) using a 20x lens with sequential, resonant, tile

scan mode. Laser lines and detector settings for sequence 1 (405/410-462, DAPI), sequence 2 (458/462-543, CFP and 555/565-632, RFP), sequence 3 (514/530-558, YFP), and sequence 4 (488/490-507, GFP and 653/660-800, Alexa Fluor-647) were selected to avoid spectral overlap. Images were analysed in Imaris v9.2.

Human arteries were formaldehyde-fixed and paraffin-embedded (FFPE) and sections (4 μm) were dewaxed, processed for antigen retrieval and sequential sections were either H&E stained, or co-stained for $\alpha$SMA, detected with biotin-coupled anti-Mouse (DAKO, E0433) and Vectastain avidin-coupled alkaline phosphatase with Blue AP substrate solution (Vector Labs), and either RUNX1, TIMP1 or CD74, that were detected with HRP-conjugated anti-Rabbit (Cell Signaling Technology, 8114), using DAB peroxidase substrate (SignalStain), before mounting in VectaMount mounting media (Vector Labs).

Cultured cells were fixed in 4% paraformaldehyde for 10 minutes at room temperature, washed in PBS, blocked and permeabilised with PBS with 0.3% Triton X-100, 5% goat serum for 1 hour, followed by incubation with primary antibody diluted in 0.1% Triton PBS 1% BSA for 1 h at room temperature (RT). Cells were washed with PBS, and incubated with the relevant Alexa Fluor 647-conjugated secondary antibody (Invitrogen) diluted in PBS with 0.1% Triton X-100 and 1% BSA for 1 hour at RT. Cells were imaged using an Opera Phenix high content screening system, with a x20 water objective (Perkin Elmer). Image analysis was done using Harmony software (v5, Perkin Elmer), quantification based on intensity values with nuclei defined by DAPI staining, and for mouse VSMCs from Myh11-EYFP animals, within cell borders (EYFP).

***EdU incorporation assay***

To assess proliferation, cells seeded in 96-well imaging plates (10,000 per well) were incubated with EdU (10 μM) for 16 hours following treatment (TIMP1/RUNX1 siRNA/OE) and EdU incorporation was detected using the Click-iT EdU kit (C10340, Thermo Fisher Scientific). Briefly, cells were fixed, permeabilized and incubated with the Click-iT reaction cocktail (containing $CuSO_4$, Alexa Fluor 647-conjugated azide, reaction buffer and additive) for 30 minutes protected from light. Next, cells were washed and stained with DAPI for 10 minutes and imaged using an Opera Phenix high content screening system (Perkin Elmer). Quantification of EdU+ cells was done using Harmony software (Perkin Elmer), by thresholding of intensity values measured in detected nuclei.

***Western blotting***

Whole cell protein lysates were prepared in RIPA buffer freshly supplemented with proteinase inhibitors (Millipore) and phosphatase inhibitors (Millipore). Protein concentration was determined using the BCA method (23227, Pierce BCA protein assay kit, Thermo Fisher). Immunoblotting was performed according to standard conditions, using gradient (4-12%) polyacrylamide gels, methanol-based wet transfer and chemiluminescence detection (Amersham ECL detection reagent, GE Healthcare). Primary antibodies were detected using HRP-labelled secondary antibodies.

**Supplementary references (also included in the main text)**

1. Schoeps, B. *et al.* Identification of invariant chain CD74 as a functional receptor of tissue inhibitor of metalloproteinases-1 (TIMP-1). *J. Biol. Chem.* **297**, 101072 (2021).

2. Ma, W., Noble, W. S. & Bailey, T. L. Motif-based analysis of large nucleotide data sets using MEME-ChIP. *Nat. Protoc.* **9**, 1428–1450 (2014).

**Supplementary Fig. 1:**
**SCA1 FACS sorting gating strategy (Fig. 1)**

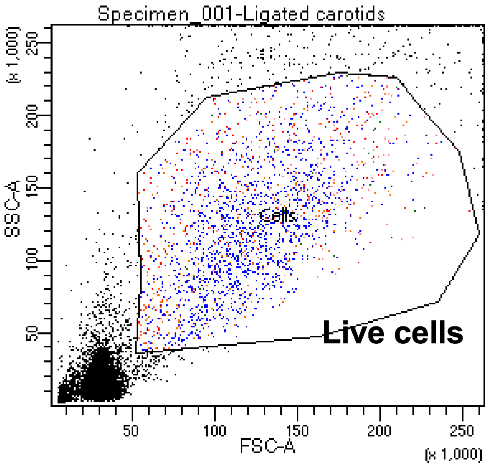


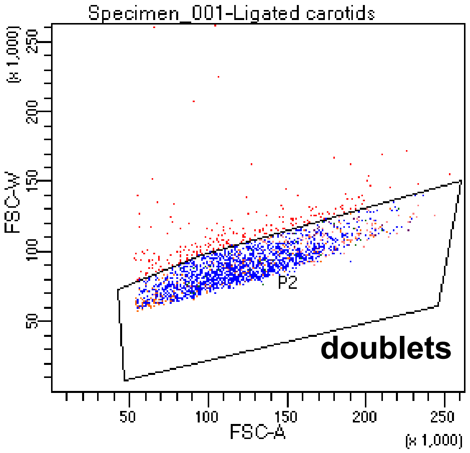


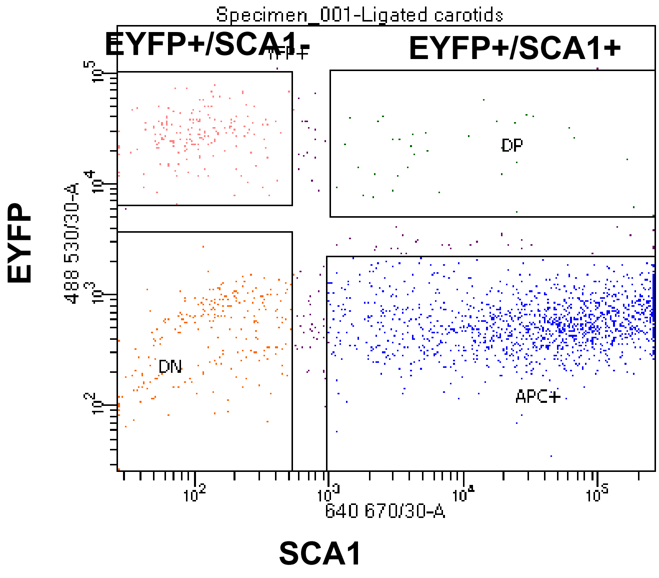


Tube: Ligated carotids

| Population | #Events | %Parent | %Total |
|---|---|---|---|
| All Events | 10,000 | #### | 100.0 |
| Cells | 2,132 | 21.3 | 21.3 |
| P2 | 1,921 | 90.1 | 19.2 |
| DN | 239 | 12.4 | 2.4 |
| YFP+ | 175 | 9.1 | 1.8 |
| APC+ | 1,388 | 72.3 | 13.9 |
| DP | 35 | 1.8 | 0.4 |

| | |
|---|---|
| Experiment Name: | Matt Worssam 290519 |
| Specimen Name: | Specimen_001 |
| Tube Name: | Ligated carotids |
| Record Date: | May 29, 2019 4:47:56 PM |
| $OP: | Administrator |
| GUID: | 16e40c82-2196-4bb2-b3c6-53d... |

| Population | #Events | %Parent | FSC-A Mean | FSC-W Mean |
|---|---|---|---|---|
| All Events | 10,000 | #### | 59,450 | 68,295 |
| Cells | 2,132 | 21.3 | 122,514 | 92,276 |
| P2 | 1,921 | 90.1 | 123,018 | 89,654 |
| DN | 239 | 12.4 | 108,209 | 82,663 |
| YFP+ | 175 | 9.1 | 155,287 | 102,689 |
| APC+ | 1,388 | 72.3 | 120,794 | 88,886 |
| DP | 35 | 1.8 | 136,512 | 96,021 |

**Supplementary Fig. 2:**
**EdU gating strategy (Fig 6d)**

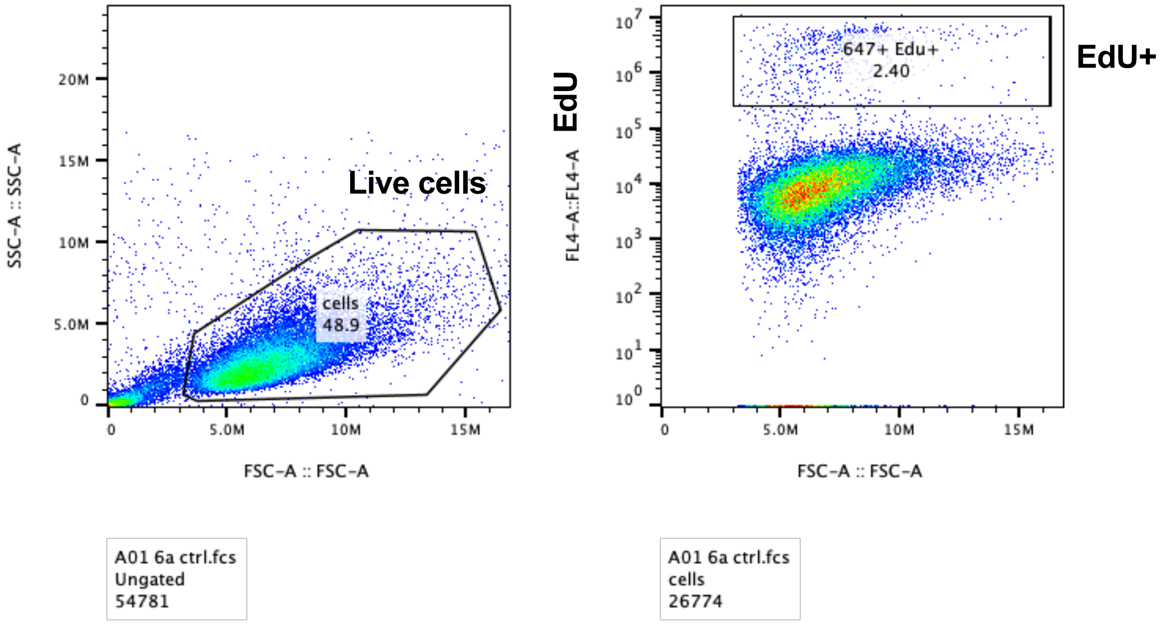


| | cells Count | cells / 647+ Edu+ Count | [illegible] |
|---|---|---|---|
| A01 6a ctrl.fcs | 26774 | 642 | |

**Supplementary Fig. 3:**
**Imagestream gating strategy (Fig. 8f-h, Extended Data Fig. 8c)**

## Gating cells from beads

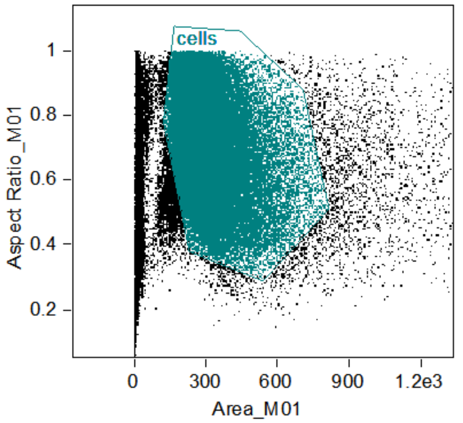


## in focus cells

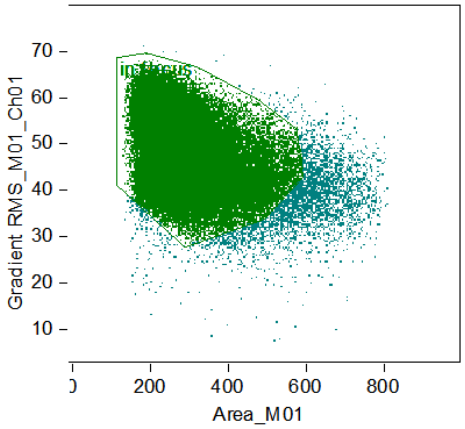


## EYFP+ cells in focus

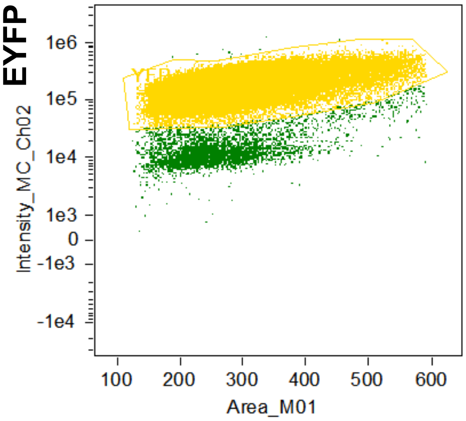


## CD74+EYFP+ cells in focus

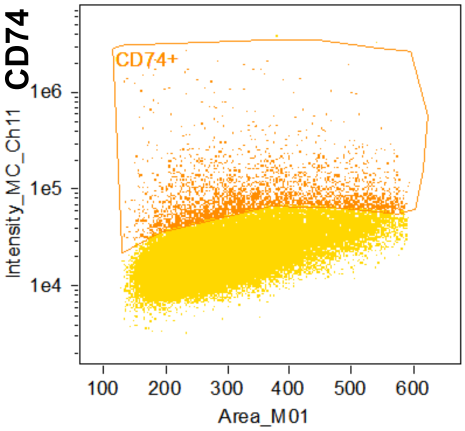


## Ki67+EYFP+ cells in focus

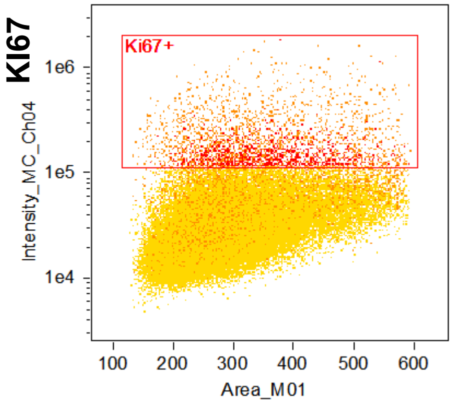


| Population | Count | %Gated |
|---|---|---|
| cells | 112178 | 62.88 |
| in focus | 108422 | 96.65 |
| YFP+ | 103773 | 95.71 |
| CD74+ | 3039 | 2.93 |
| Ki67+ | 1661 | 54.66 |
| Ki67+ | 2872 | 2.77 |
| CD74+ | 1661 | 57.83 |
| Ki67+Cd74- | 1010 | 35.17 |
| ki67- | 94308 | 90.88 |

**Supplementary Fig. 4:**
**Enlarged view of Fig. 2d**

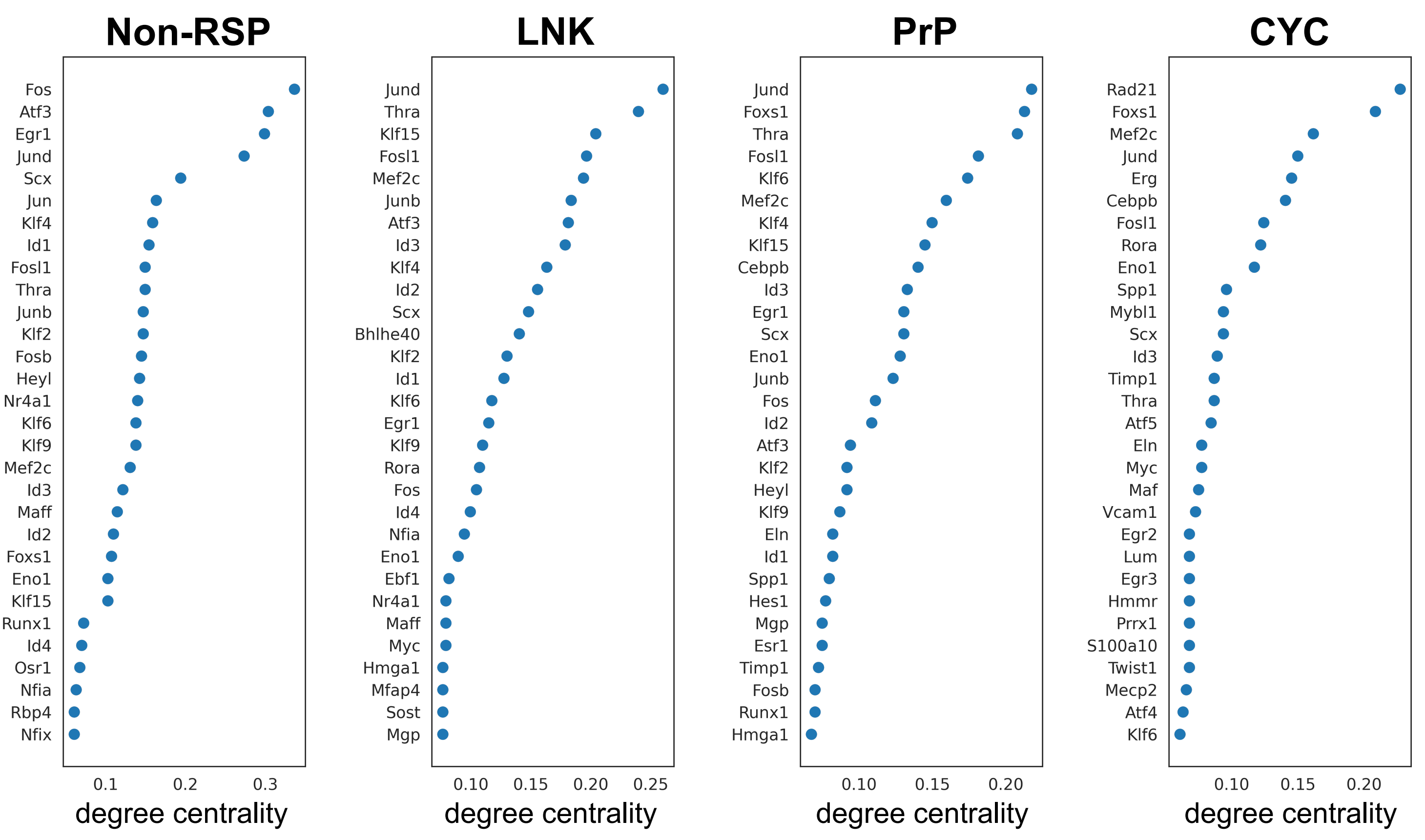


**Enlarged view of Fig. 2e**

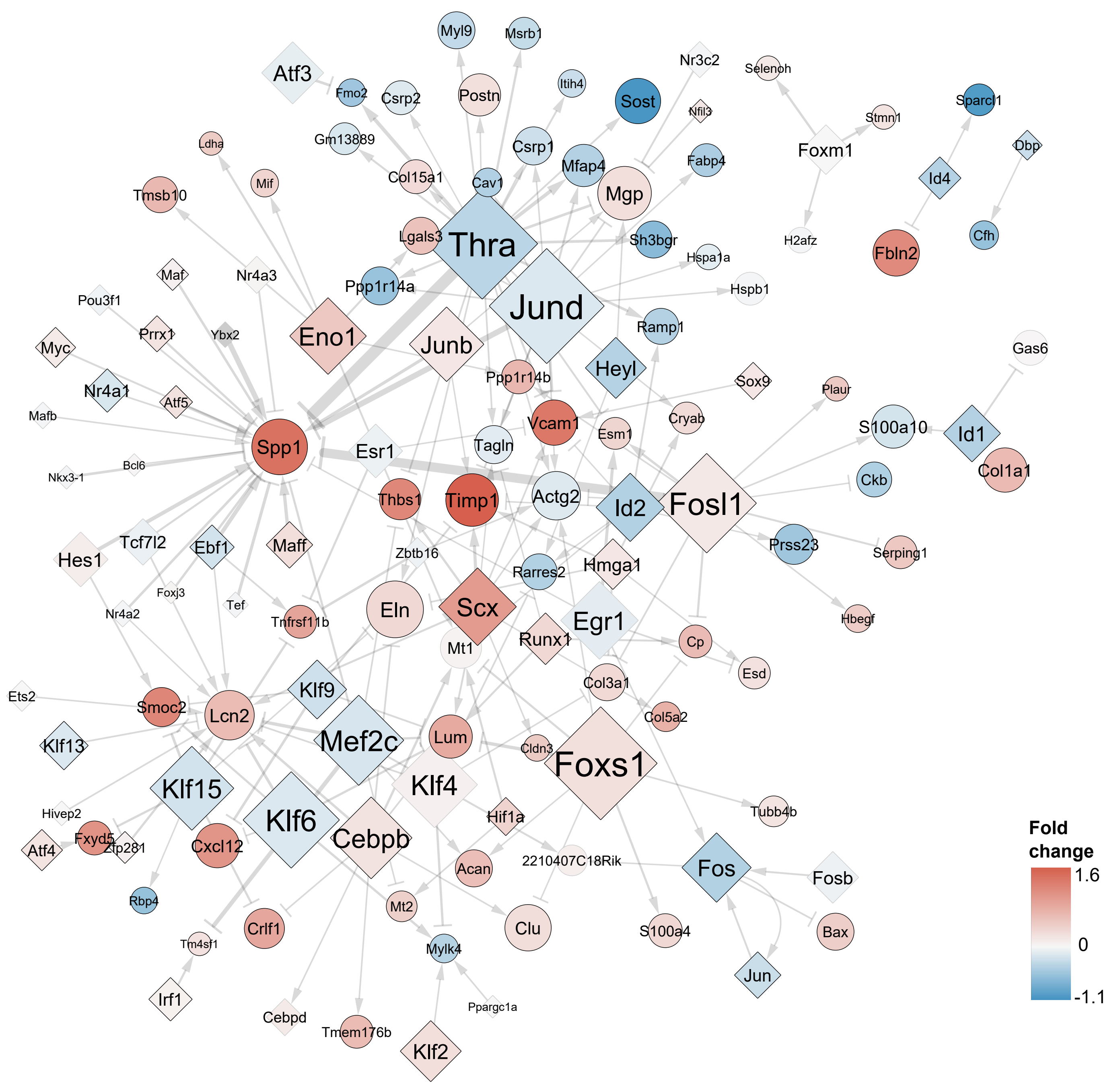


**Enlarged view of Fig. 2f**

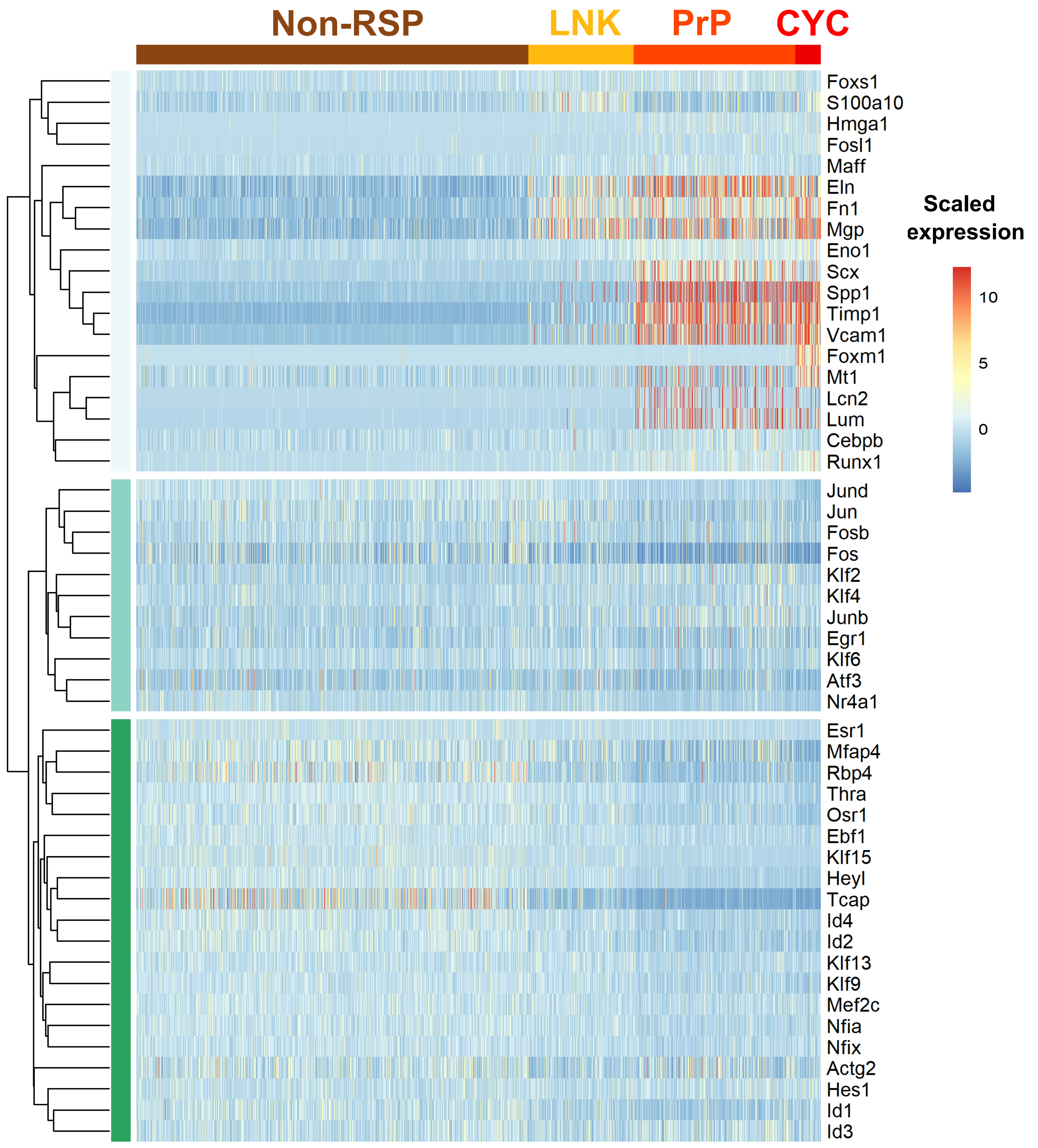

**Supplementary Fig. 5:**
**Enlarged view of Fig. 3c**

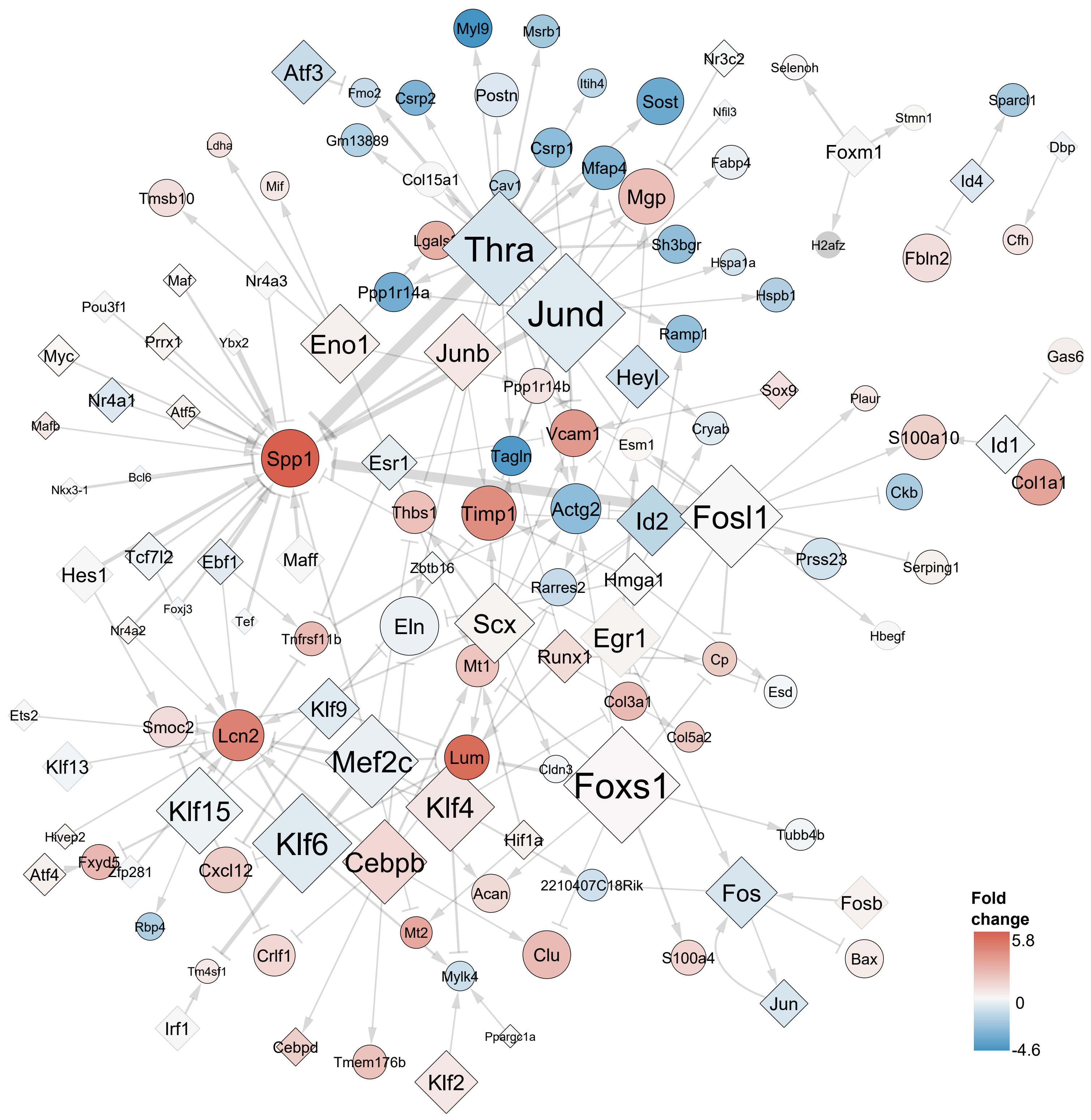


**Enlarged view of Fig. 3f**

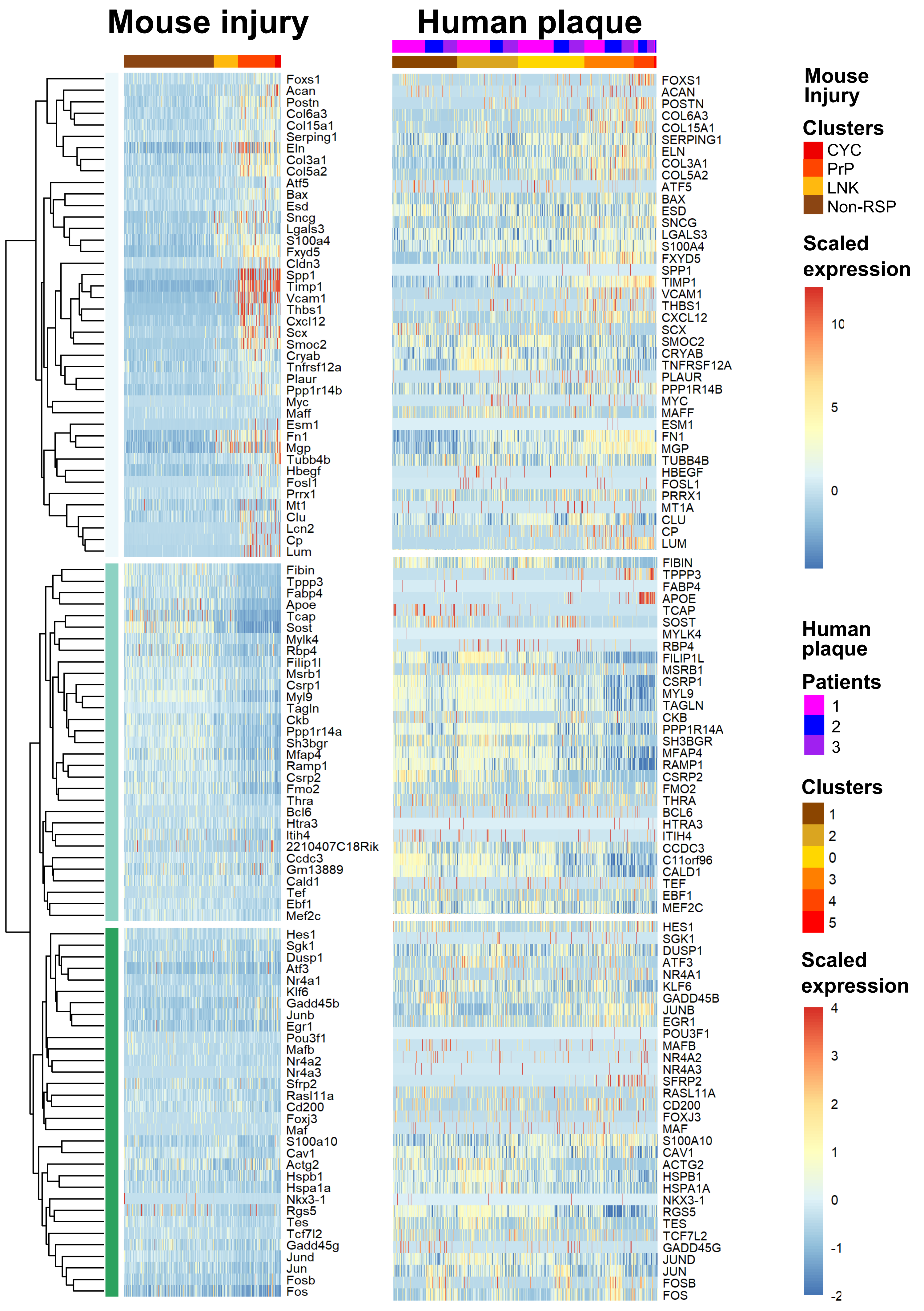

**Supplementary Fig. 6:**
**Enlarged view of Extended Data Fig. 2d**

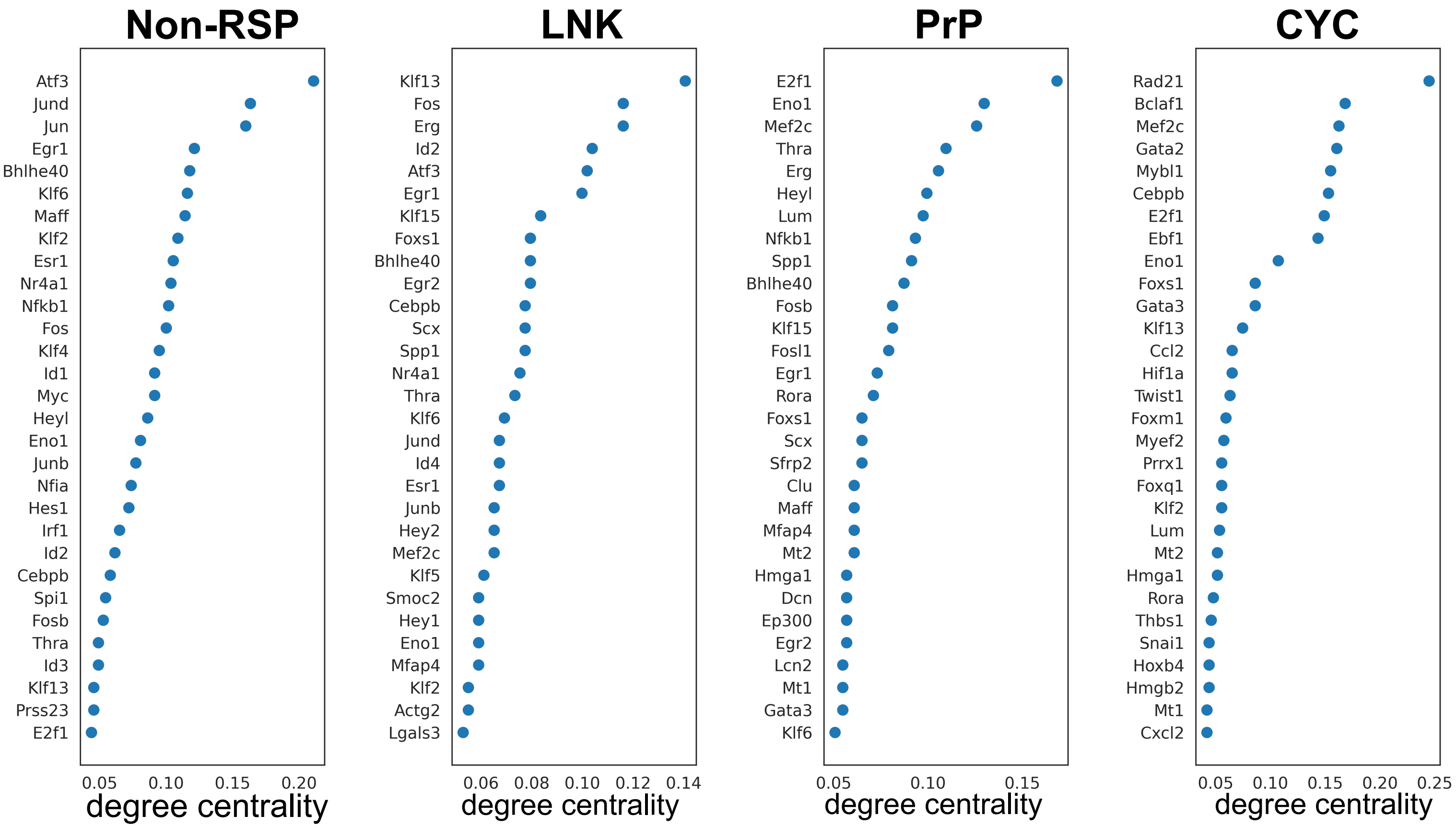

Supplement: Supplementary file 2 [file 44161_2024_474_MOESM1_ESM.pdf]
