# Supplementary material for: Network-based prioritization and validation of regulators of vascular smooth muscle cell proliferation in disease
Source: Nat Cardiovasc Res. 2024 Jun 6;3(6):714–33. doi: 10.1038/s44161-024-00474-4 (PMC11182749; doi:10.1038/s44161-024-00474-4)
Supplement: Supplementary file 3 [file 44161_2024_474_MOESM2_ESM.pdf]

nature portfolio

Corresponding author(s): Helle F Jørgensen

Last updated by author(s): Apr 16, 2024

Reporting Summary

Nature Portfolio wishes to improve the reproducibility of the work that we publish. This form provides structure for consistency and transparency in reporting. For further information on Nature Portfolio policies, see our Editorial Policies and the Editorial Policy Checklist.

Statistics

For all statistical analyses, confirm that the following items are present in the figure legend, table legend, main text, or Methods section.

n/a	Confirmed	
☐	☒	The exact sample size (*n*) for each experimental group/condition, given as a discrete number and unit of measurement
☐	☒	A statement on whether measurements were taken from distinct samples or whether the same sample was measured repeatedly
☐	☒	The statistical test(s) used AND whether they are one- or two-sided<br>*Only common tests should be described solely by name; describe more complex techniques in the Methods section.*
☒	☐	A description of all covariates tested
☐	☒	A description of any assumptions or corrections, such as tests of normality and adjustment for multiple comparisons
☐	☒	A full description of the statistical parameters including central tendency (e.g. means) or other basic estimates (e.g. regression coefficient) AND variation (e.g. standard deviation) or associated estimates of uncertainty (e.g. confidence intervals)
☐	☒	For null hypothesis testing, the test statistic (e.g. *F*, *t*, *r*) with confidence intervals, effect sizes, degrees of freedom and *P* value noted<br>*Give P values as exact values whenever suitable.*
☒	☐	For Bayesian analysis, information on the choice of priors and Markov chain Monte Carlo settings
☒	☐	For hierarchical and complex designs, identification of the appropriate level for tests and full reporting of outcomes
☒	☐	Estimates of effect sizes (e.g. Cohen's *d*, Pearson's *r*), indicating how they were calculated

Our web collection on statistics for biologists contains articles on many of the points above.

Software and code

Policy information about availability of computer code

Data collection	IDEAS® Image Analysis Software v.6.2 was used to collect data for imaging flow cytometry
Data analysis	ATAC-seq raw sequence reads were assessed using the FASTQC package (Andrews, 2010). Adaptor sequences and base-calls with Phred score < 20 were removed using Trim Galore v.0.6.1, running Cutadapt v.1.18. Alignment to the GRC38.98 Mus musculus genome was done using Bowtie2 v.2.3.5. BAM files were generated using samtools v.1.18 and BigWig files using deeptools v.3.4.3. Peak-calling was done using MACS2 v.2.2.7.1. Genomic features annotation was done using ChIPseeker v.1.24.0. Differential accessibility was scored in LIMMA (SeqMonk v.1.47.2). Peaks were annotated to genes and gene ontology (GO) term enrichment analysis was performed using the online version of GREAT v.4.0.4 (McLean et al, 2010) (http://great.stanford.edu/public/html/index.php). ATAC-seq peak summits were identified using the refinePeak command in MACS2 v.2.2.7.1 (Zhang et al., 2008). Masking of repetitive elements was done using RepeatMasker v.4.1.1 (https://www.repeatmasker.org/cgi-bin/WEBRepeatMasker). Motif enrichment analysis was done using MEME-ChIP v.5.4.1 (Ma et al, 2014). HOCOMOCO mouse (v.11) was used as the transcription factor motif database.<br><br>Gene regulatory networks (GRNs) were modelled using CellOracle v.0.10.5, and visualised with Cytoscape v.3.7.2. CellOracle v.0.10.5 was also used for in silico perturbation experiments and for calculation of perturbation scores. Venn and Euler diagrams were created by using CRAN R package eulerr v.7.0.0. Gene Ontology term analysis and visualization were performed with BiNGO v.3.0.4 and GOlorize v.1.0.0.beta1. Communities were detected using clusterMaker2 v.1.3.1 (Morris et al, 2011) with the MCODE algorithm (Bader & Hogue, 2003)(fluff option on). Differential gene expression was calculated using Seurat v.4.3.0. Rewiring calculations was done using iGraph v.1.4.2. Heatmaps were created with pheatmap v.1.0.12. Gene set signatures were assessed by using UCell v.2.2.0<br><br>Colocalisation analysis was done using eCAVIAR (Hormozdiari et al. 2016) and SMR v1.3.1 (Zhu et al. 2016).

Image analysis was done using Harmony v5, Fiji v2.15.1 and Imaris v9.2.

Flow cytometry data was analysed in FlowJo v10.8.1

For manuscripts utilizing custom algorithms or software that are central to the research but not yet described in published literature, software must be made available to editors and reviewers. We strongly encourage code deposition in a community repository (e.g. GitHub). See the Nature Portfolio guidelines for submitting code & software for further information.

Data

Policy information about availability of data

All manuscripts must include a data availability statement. This statement should provide the following information, where applicable:

- Accession codes, unique identifiers, or web links for publicly available datasets
- A description of any restrictions on data availability
- For clinical datasets or third party data, please ensure that the statement adheres to our policy

The ATAC-seq datasets (GSE246646) and bulk RNA-seq data from TIMP1-treated hVSMCs and controls (GSE246647) have been deposited to the NCBI gene expression omnibus, (GEO). ScRNA-seq datasets of VSMCs from mouse arteries after injury (GSE162167, (Worssam et al, 2022)), murine atherosclerosis (GSE155513, (Pan et al, 2020)) and human carotid plaque cells (GSE155512, (Pan et al, 2020)) are available from GEO. Other data will be made available upon request. CAD GWAS (Aragam et al, 2022) was downloaded from the GWAS Catalog. The cis-eQTL data are VSMC cis-eQTL from smooth muscle cells isolated from human umbilical cord (n = 1499) (Solomon et al, 2022, PMID: 35735005) and human aortic smooth muscle cell (HAoSMC) cis-eQTL for quiescent (n = 139) and proliferative (n = 145) cells (Aherrahrou et al, 2023, 36597873). Source data for experimental analysis is provided as Supplementary information.

Research involving human participants, their data, or biological material

Policy information about studies with human participants or human data. See also policy information about sex, gender (identity/presentation), and sexual orientation and race, ethnicity and racism.

Reporting on sex and gender	Cell isolates from a total of 12 donors (age and sex; 27M, 20M, 70M, 75F, 45F, 65M, 62F, 51M, 68F, 69M, 72F, 68M, 61M) and histological analysis of 9 carotid artery samples (72F, 82M, 80F, 67M, 67F, 58F, 74M, 54M and 50F) and 10 aorta (60M, 24M, 84F, 65M, 49M, 35M, 41M, 75F, 18M, 59F) were included. Both male and female tissue and cell isolates were included in all analyses. Gender information is not available
Reporting on race, ethnicity, or other socially relevant groupings	Tissue was obtained from anonymised donors and this information is not available.
Population characteristics	Not applicable
Recruitment	Recruitment was done by the Papworth Tissue Bank after informed consent (Tissue donors did not receive compensation).
Ethics oversight	Human arteries were collected after informed consent and approval by the Cambridgeshire 1 or East of England - Cambridge South Research Ethics Committee (REC ref 15/EE/1052, H00/514).

Note that full information on the approval of the study protocol must also be provided in the manuscript.

Field-specific reporting

Please select the one below that is the best fit for your research. If you are not sure, read the appropriate sections before making your selection.

☒ Life sciences ☐ Behavioural & social sciences ☐ Ecological, evolutionary & environmental sciences

For a reference copy of the document with all sections, see nature.com/documents/nr-reporting-summary-flat.pdf

Life sciences study design

All studies must disclose on these points even when the disclosure is negative.

Sample size	Group size and replicate numbers were decided following power calculations based on observed or expected variation using a power of 90% and a 0.05% type I error rate. Where technical repeats were conducted of the same cell line/ animal, these have been averaged and biological repeats used for statistical analysis.
Data exclusions	No data points were excluded from analysis
Replication	ATAC-seq was performed in parallel for each condition: SCA1- and SCA1+ cells were isolated from a single cell suspension of injured arteries, and control cells were isolated from littermate controls. Two independent experiments were performed on different days with independent animals; replicate profiles showed strong correlation. GRN construction was replicated in two different dataset producing similar results. VSMC clonal proliferation assays were done with cells from independent animals (seeded in triplicate), in at least two batches run independently - none of the data were excluded. Experiments with human VSMCs were done using VSMC isolates from different donors. Technical replicates of each sample were included in e.g. in qPCR analyses and clonal proliferation assays, but the data shown are averages of

technical replicates for each independent sample.

Randomization

For the in vivo TIMP1 treatment experiment, animals were randomised into control (PBS) and TIMP1 groups.

Blinding

Images were blinded during quantification of VSMC patches in the clonal proliferation assay, quantification of immunofluorescence staining and throughout processing samples from TIMP1/control-injected animals. For the in vivo TIMP1 treatment experiment, the personnel performing injections were aware of the group allocation (due to repeat injections), but the data collection was done in a blinded fashion and unblinding was not done until after sample processing, data collection and data analysis.

Reporting for specific materials, systems and methods

We require information from authors about some types of materials, experimental systems and methods used in many studies. Here, indicate whether each material, system or method listed is relevant to your study. If you are not sure if a list item applies to your research, read the appropriate section before selecting a response.

Materials & experimental systems

n/a	Involved in the study	
☐	☒	Antibodies
☐	☒	Eukaryotic cell lines
☒	☐	Palaeontology and archaeology
☐	☒	Animals and other organisms
☒	☐	Clinical data
☒	☐	Dual use research of concern
☒	☐	Plants

Methods

n/a	Involved in the study	
☒	☐	ChIP-seq
☐	☒	Flow cytometry
☒	☐	MRI-based neuroimaging

Antibodies

Antibodies used

arget ,Clone,Source,Cat number,Lot number,Assay ,"Dilutions, or final concentrations"
CD74,clone LN1,Biolegend,151002,B227379,IF-cryosection/IF-culture,10 ug/ml
CD74,clone LN1,Biolegend,151003,B331359,Imagestream,2.5 ug / mL
CD74 blocking antibody,clone LN-2,Santa Cruz,sc-6262,lot J1121,IHC-FFPE,0.4 ug / mL
TIMP1,polyclonal,Proteintech,16644-1-AP,23865,IF-cryosections,2.5 ug / mL
TIMP1,clone F31 P2 A5,ThermoFisher,MA1-773,,IHC-FFPE,1 ug / mL
STAT3 pS727,polyclonal,Abcam,ab86430,GR3359229-3,IF-cryosections/IF-culture/Imagestream,1ug/mL
STAT3 antibody,Clone D3Z2G,Cell Signaling Technology,12640,,ChIP,10 ul per 5 ug chromatin
IgG,isotype ctrl,Cell Signaling Technology,2729,,ChIP,1 ul per 5 ug chromatin
total STAT3,124H6,Cell Signaling Technologies,9139,lot 12,WB,1 in 1000
STAT3 pS727,polyclonal,Cell Signaling Technologies,9134,lot 21,WB,1 in 1000
STAT3 pTyr705,D3A7,Cell Signaling Technologies,9145,lot 43,WB,1 in 1000
total AKT,2C5D1,Proteintech,60203-2-IG,lot 10022173,WB,1 in 6000
AKT S473,polyclonal ,Cell Signaling Technologies,9271,lot 15,WB,1 in 1000
total P38,M138,Abcam,ab31828,lot GR3358093-1,WB,1 in 1000
P38 pThr180/Tyr182,polyclonal ,Cell Signaling Technologies,9211,lot 25,WB,1 in 1000
GAPDH,14C10,Cell Signaling Technologies,2118,lot 16,WB,1 in 3000
KI67,MIB-1,Santa Cruz,sc-101861,12418,Imagestream,1 in 200
KI67,SolA15,eBioscience,14-5698-80,lot 2383503,IF-culture,5 ug / mL
aSMA,1A4,DAKO,M0851,multiple,IHC-FFPE,1 in 400
RUNX1,clone A-2,Santa Cruz,sc-365644,lot F2822,IHC-FFPE/IF-culture,2 ug / mL
MMP14,polyclonal,Abcam,ab53712,lot GR3231349-15,Imagestream,1 in 100
SCA1,D7 ,Miltenyi,130-102-343,lot 5141007347,Imagestream,2 ug / mL
RUNX1,polyclonal,Abcam,ab23980,,IF-cryo,1 in 200
Rabbit IgG,SignalStain® Boost IHC Detection Reagent ,Cell Signaling Technologies,8114,multiple,IHC-FFPE,optimised for each antibody
Rabbit IgG,HRP-linked,Cell Signaling Technologies,7074S,multiple,WB secondary,1 in 3000

Validation

Target ,Clone,Source,Cat number,validation
CD74,clone LN1,Biolegend,151002,Validated in house via singal over IgG and subcellular localisation. Manufacturer validation statement: Each lot of this antibody is quality control tested by immunofluorescent staining with flow cytometric analysis.
CD74,clone LN1,Biolegend,151003,Validated in house via singal over IgG and subcellular localisation. Manufacturer validation statement: Each lot of this antibody is quality control tested by immunofluorescent staining with flow cytometric analysis.
CD74 blocking antibody,clone LN-2,Santa Cruz,sc-6262,CD74 blocking antibody - confirmed using blocking peptide. References for use as a blocking antibody; https://doi.org/10.4049/jimmunol.177.12.8730
TIMP1,polyclonal,Proteintech,16644-1-AP,"antigen affinity purified and has been used in published studies 17 times, signal was compared to appropritate isotype control "
TIMP1,clone F31 P2 A5,ThermoFisher,MA1-773,staining of negative and postitive control tissues was performed in parallel. Manufacturer validation statment: Advanced Verification
,,,,This Antibody was verified by Cell treatment to ensure that the antibody binds to the antigen stated.
STAT3 pS727,polyclonal,Abcam,ab86430,"Validated in house via signal over IgG and subcellular localisation. Manufacturer validation statement: Ab86430 has <30% cross reactivity with unmodified STAT3 as determined by ELISA, according to the vendor"
STAT3 antibody,Clone D3Z2G,Cell Signaling Technology,12640,"Antibody was validated by no qPCR amplication at a control, non-

binding locus. Manufacturer validation statement: This antibody has been validated using SimpleChIP® Enzymatic Chromatin IP Kits. Antibody has been referenced for ChIP 22 times. "
IgG,isotype ctrl,Cell Signaling Technology,2729,NA
total STAT3,124H6,Cell Signaling Technologies,9139,validated by Molecular weight assessment and QC'ed by the vendor
STAT3 pS727,polyclonal,Cell Signaling Technologies,9134,validated by Molecular weight assessment and QC'ed by the vendor
STAT3 pTyr705,D3A7,Cell Signaling Technologies,9145,validated by Molecular weight assessment and QC'ed by the vendor
total AKT,2C5D1,Proteintech,60203-2-IG,validated by Molecular weight assessment and QC'ed by the vendor
AKT S473,polyclonal ,Cell Signaling Technologies,9271,validated by Molecular weight assessment and QC'ed by the vendor
total P38,M138,Abcam,ab31828,validated by Molecular weight assessment. Manufacturer validation statement: this antibody has been KO validated.
P38 pThr180/Tyr182,polyclonal ,Cell Signaling Technologies,9211,validated by Molecular weight assessment and QC'ed by the vendor
GAPDH,14C10,Cell Signaling Technologies,2118,validated by Molecular weight assessment and QC'ed by the vendor
KI67,MIB-1,Santa Cruz,sc-101861,"Antibody was validated by subcellular localisation, %proliferating cells compared to existing EdU data, and use of positive control cells (splenocytes)"
KI67,SolA15,eBioscience,14-5698-80,"Antibody was validated by subcellular localisation, %proliferating cells compared to existing EdU data, and use of positive control cells (splenocytes). Manufacturer validation statement: Advanced validation. This Antibody was verified by Cell treatment to ensure that the antibody binds to the antigen stated."
aSMA,1A4,DAKO,M0851,staining of negative control tissue was performed in parallel
RUNX1,clone A-2,Santa Cruz,sc-365644,staining of negative control tissue was performed in parallel
MMP14,polyclonal,Abcam,ab53712,MMP14 has been referenced 29 times
SCA1,D7 ,Miltenyi,130-102-343,"SCA1 was tested using VSMCs from healthy arteries as (general) negative control (<1% positive) and using adventitial fibroblasts as a positive control, and tested for singal above IgG control"
RUNX1,polyclonal,Abcam,ab23980,subcellular localisation
Rabbit IgG,"SignalStain® Boost IHC Detection Reagent (HRP, Rabbit)",Cell Signaling Technologies,8114,staining of negative control tissue was performed in parallel
Rabbit IgG,"Anti-rabbit IgG, HRP-linked",Cell Signaling Technologies,7074S,

Eukaryotic cell lines

Policy information about cell lines and Sex and Gender in Research

Cell line source(s)	All VSMCs were primary cultures derived in house; human VSMC lines were derived from both male and female donors. Murine VSMC cultures were generated from enzyme digestion of the medial layer of aortas from lineage-traced animals. Lineage tracing was done using a Y-linked Cre driver (Myh11-Creert2) so all cells were from males. HEK293F/HEK293FT cells were used for lentiviral production
Authentication	The identity of murine VMSC was verified using the lineage label (sorting, imaging). Human VSMCs were used within 10 passages to avoid drift and VSMC identity confirmed by visual inspection of cell morphology and growth rate. HEK293F/HEK293FT cells were not authenticated formally but had a characteristically high transfection efficiency and produced functional virus particles.
Mycoplasma contamination	Primary mouse VSMC were analysed within 3 weeks of culture and were not tested. Human VSMCs and HEK293F/HEK293FT cells were regularly tested for mycoplasma contamination, with negative results.
Commonly misidentified lines (See ICLAC register)	HEK293F and HEK293FT

Animals and other research organisms

Policy information about studies involving animals; ARRIVE guidelines recommended for reporting animal research, and Sex and Gender in Research

Laboratory animals	Mus musculus, C57Bl/6, 9-33 weeks, housing on a 12h dark/light cycle, at 19-21 degree C and 45-65% humidity.
Wild animals	the study did not involve wild animals
Reporting on sex	Lineage labelling was done using the Myh11-CreERt2 (offermanns) allele, which is Y-linked. Males were therefore used for the study
Field-collected samples	The study did not involve samples collected from the field
Ethics oversight	The Cambridge Animal Welfare and Ethical Review Body

Note that full information on the approval of the study protocol must also be provided in the manuscript.

Flow Cytometry

Plots

Confirm that:

☐ The axis labels state the marker and fluorochrome used (e.g. CD4-FITC).

☐ The axis scales are clearly visible. Include numbers along axes only for bottom left plot of group (a 'group' is an analysis of identical markers).

☐ All plots are contour plots with outliers or pseudocolor plots.

☐ A numerical value for number of cells or percentage (with statistics) is provided.

Methodology

Sample preparation	Mouse VSMCs were isolated by enzymatic (collagenase and elastase) digestion of freshly harvested aorta or carotid arteries (ATAC-seq), hVSMCs were cultured from human tissue samples and harvested by trypsinization. Single-cell suspensions were incubated with 5 µg/mL TruStain FcX anti-mouse CD16/32 antibody (Biolegend) in FACS buffer (0.5% (w/v) BSA in PBS) for 15 minutes on ice, stained with primary antibody for 30 minutes at room temperature and washed twice (FACS buffer). This was followed by incubation with a secondary antibody (in FACS buffer, 30 minutes at room temperature) and washed twice (FACS buffer) for non-conjugated antibodies. Intracellular targets were stained using the Foxp3 staining buffer set (eBioscience).
Instrument	Cells were analysed using an Imagestream system (Amnis® ImageStream®XMk II, Luminex), BD Accuri C6 Flow Cytometer (BD Bioscience) or sorted using a BD FACSAria™ III (BD Bioscience).
Software	Data collection and cell sorting was done using BD FACS Diva v8.0.1. For flow cytometry data was collected using BD CSampler™ software. Data was analysed in FlowJo v10.8.1. Imagestream data was collected and analysed using IDEAS v6.2..
Cell population abundance	EYFP+SCA1+ cells represented 0.4% of the total cell population. Sort conditions were optimised for an estimated >95% purity, validated by computational analysis of sorted cells.
Gating strategy	To isolate cells for ATAC-seq, a population of live cells were gated from FSC/SSC plots, singlets from FSC-A/FSC-W plots. The gate for EYFP+ VSMCs were identified based on cells from wild type cells, or from unstained adventitial cell, that do not express the VSMC lineage label. The gate for SCA1+ cells were based on a control sample stained with conjugated control IgG (negative) and adventitial cells stained for SCA1 were used as postive controls. For EdU incorporation analysis, a population of live cells were gated from FSC/SSC plots, the gate for EdU+ cells was identified based on cells without EdU added, or cells without fluorophore-conjugated click-it reagent.

☒ Tick this box to confirm that a figure exemplifying the gating strategy is provided in the Supplementary Information.
