# Supplementary material for: Network-based prioritization and validation of regulators of vascular smooth muscle cell proliferation in disease
Source: Nat Cardiovasc Res. 2024 Jun 6;3(6):714–33. doi: 10.1038/s44161-024-00474-4 (PMC11182749; doi:10.1038/s44161-024-00474-4)

Source data for Figure 2d

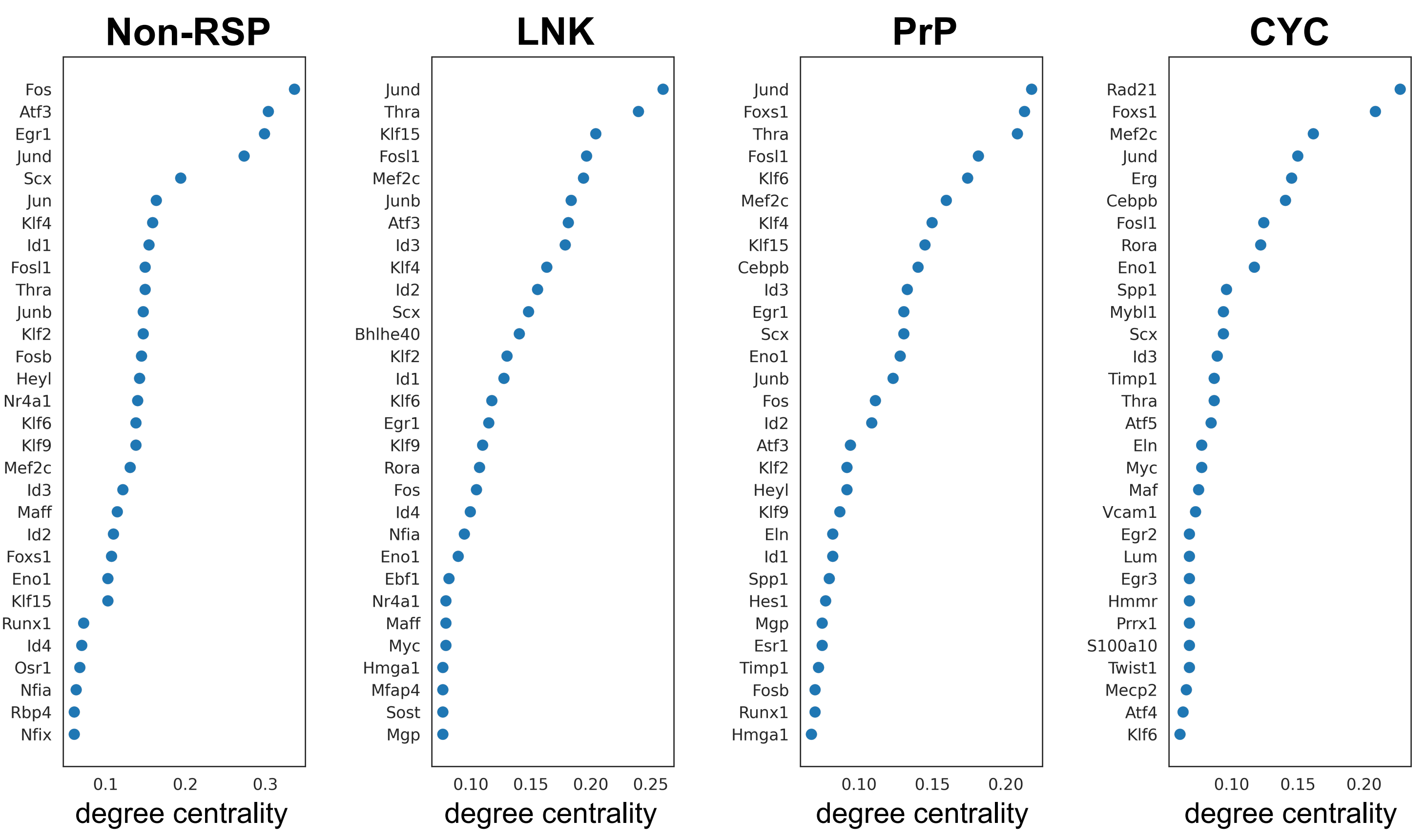

Source data for Figure 2e

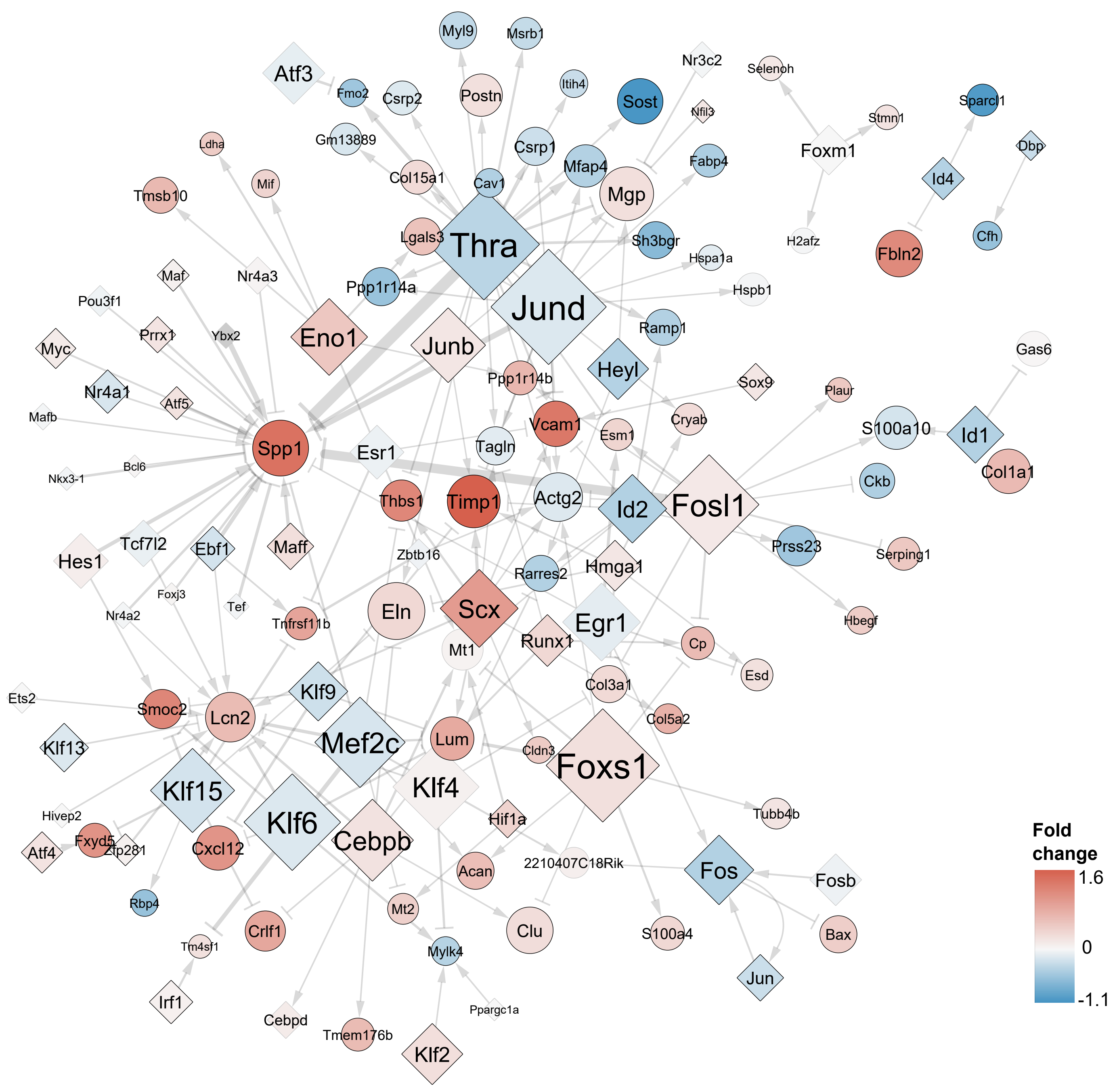

Source data for Figure 2f

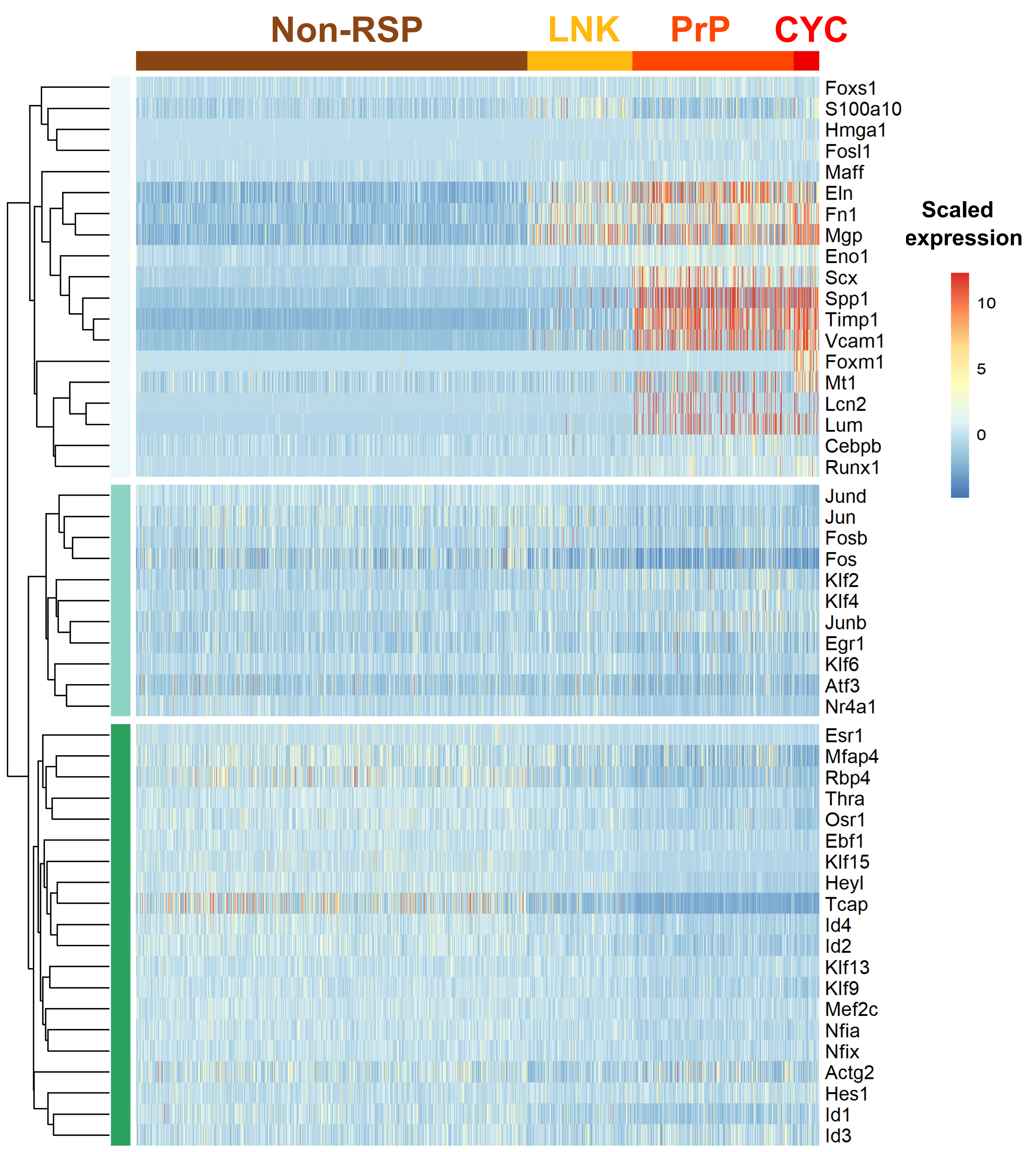

Supplement: Supplementary file 4 [file 44161_2024_474_MOESM6_ESM.pdf]
