# Supplementary material for: Network-based prioritization and validation of regulators of vascular smooth muscle cell proliferation in disease
Source: Nat Cardiovasc Res. 2024 Jun 6;3(6):714–33. doi: 10.1038/s44161-024-00474-4 (PMC11182749; doi:10.1038/s44161-024-00474-4)
Supplement: Supplementary file 6 [file 44161_2024_474_MOESM11_ESM.zip › Lambert_Oc_SourceData_fig7/Lambert_Oc_unmodified_westerns_fig7.pdf]

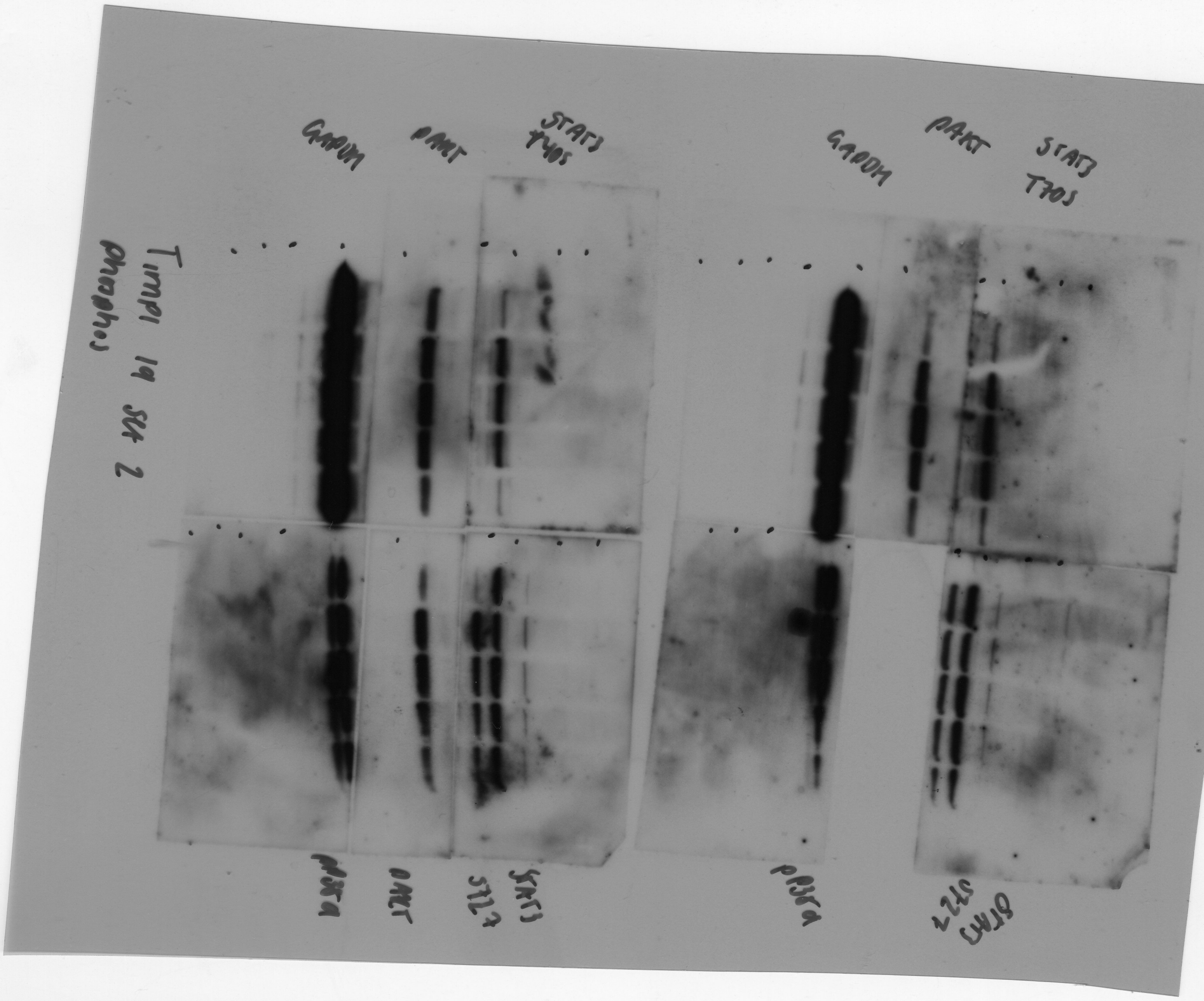
Timp1 19 seq 2 phosphos
GAPDH
pAKT
STAT3 T705
GAPDH
pAKT
STAT3 T705

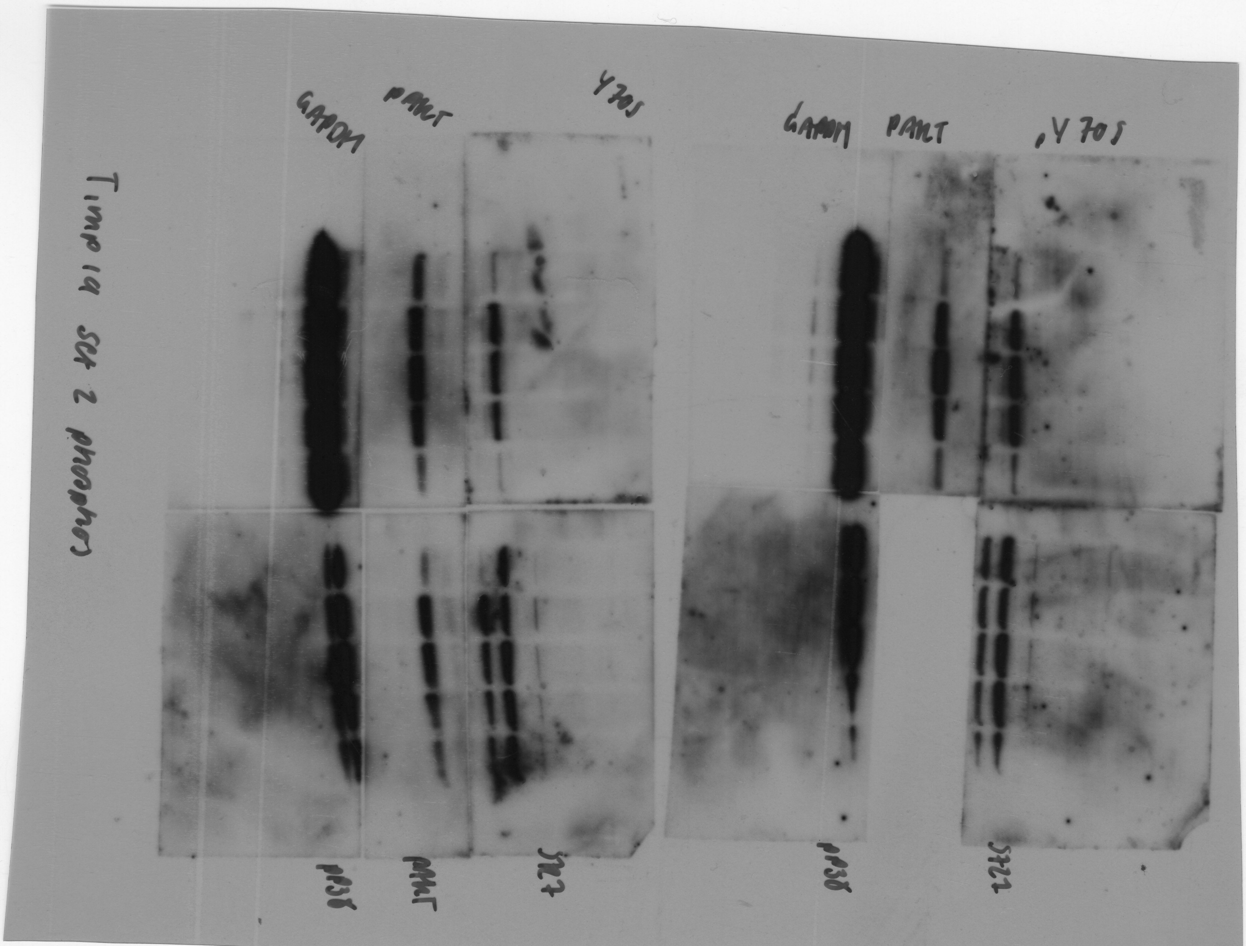
Timp1 19 seq 2 phosphos
GAPDH
pAKT
Y705
GAPDH
pAKT
Y705

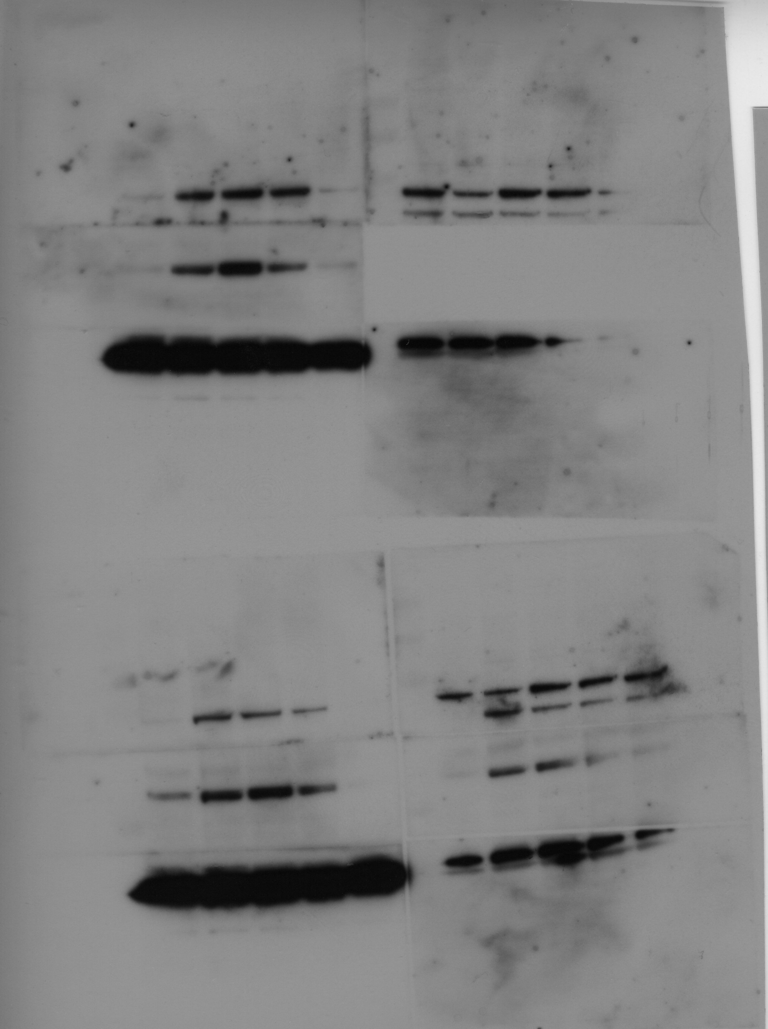

Timp1 19 set 2 phosphoproteins

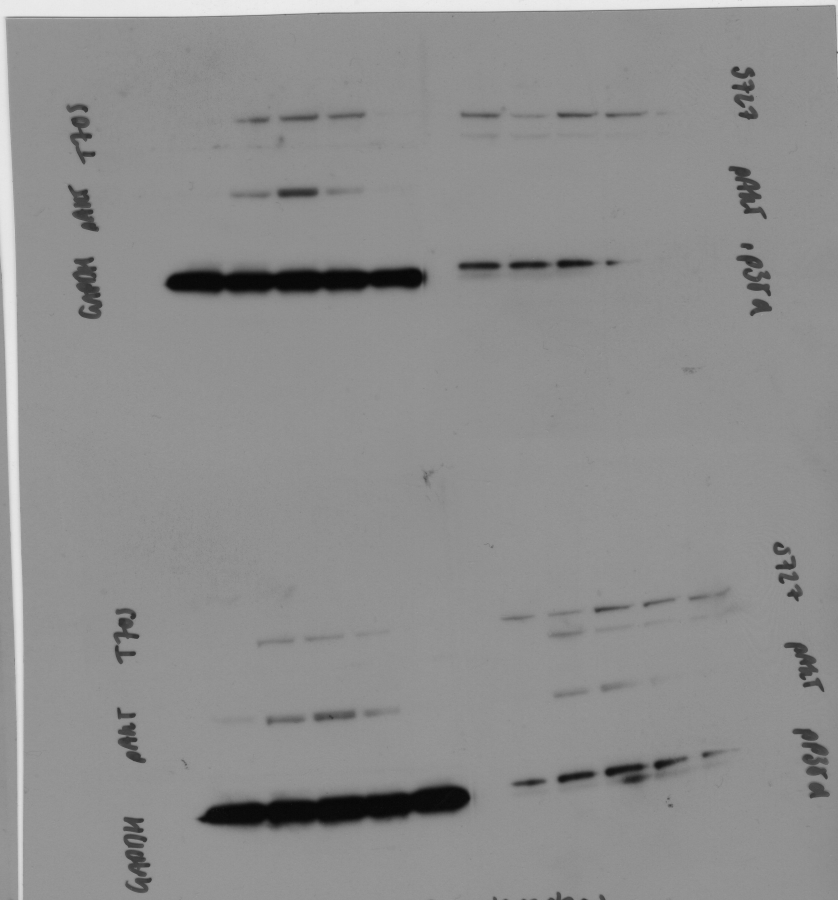

Timp1 19 set 2 phosphos

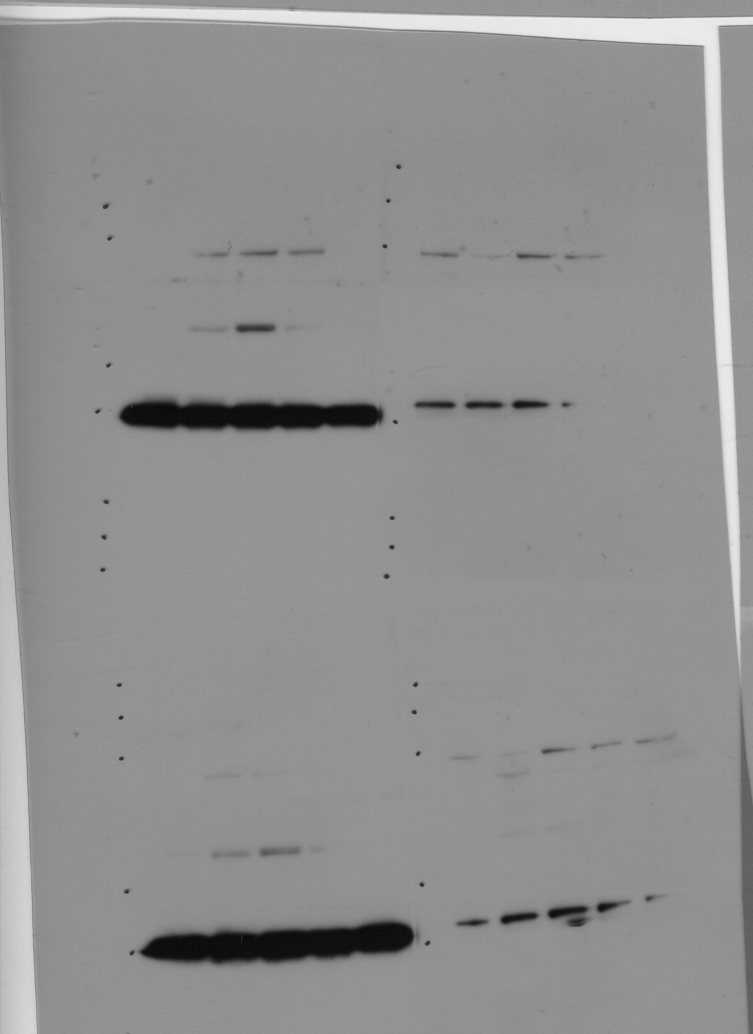

Timp1 19 phosphos1

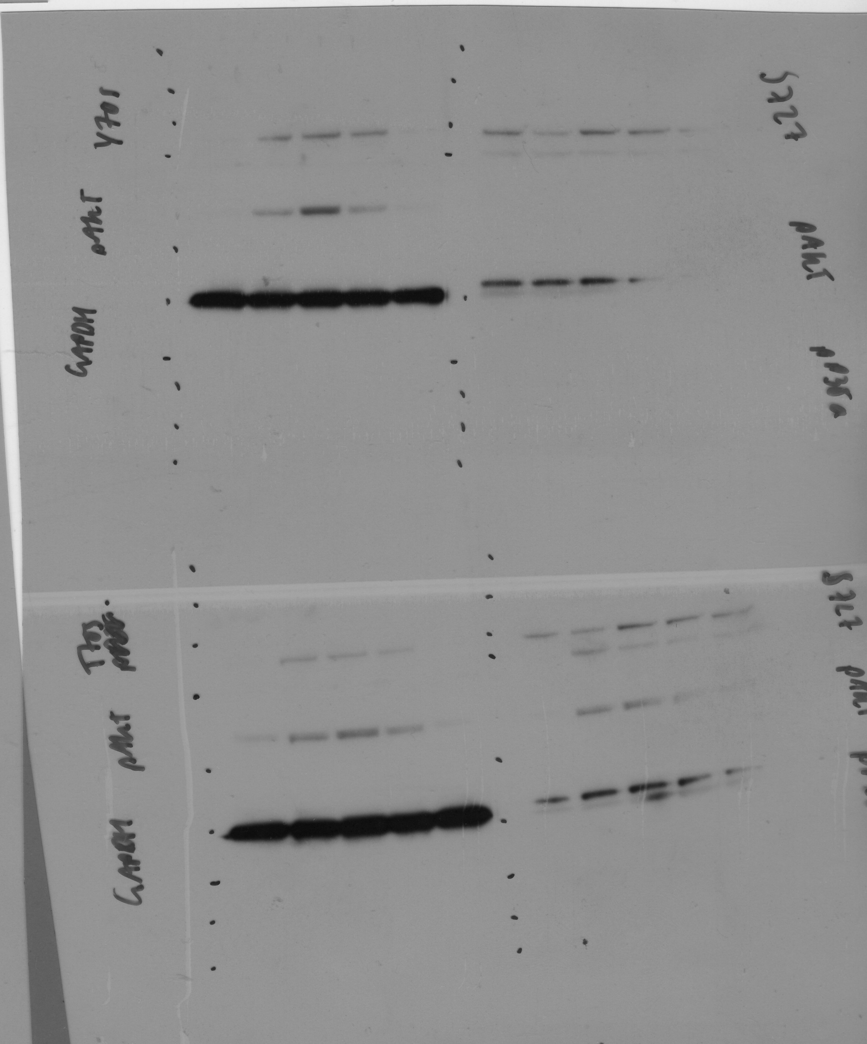

Timp1 19 set 2 phosphos

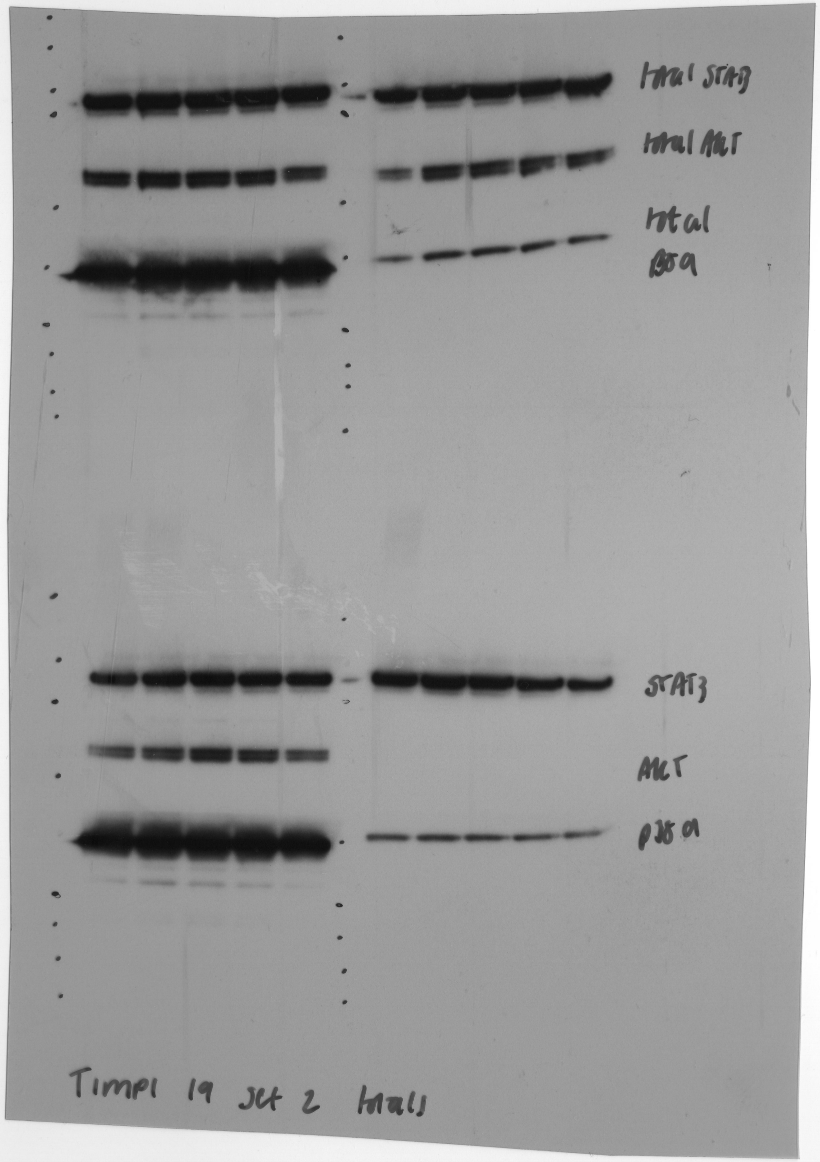
total STAT3
total AKT
total p38α
STAT3
AKT
p38α
Timp1 19 set 2 totals

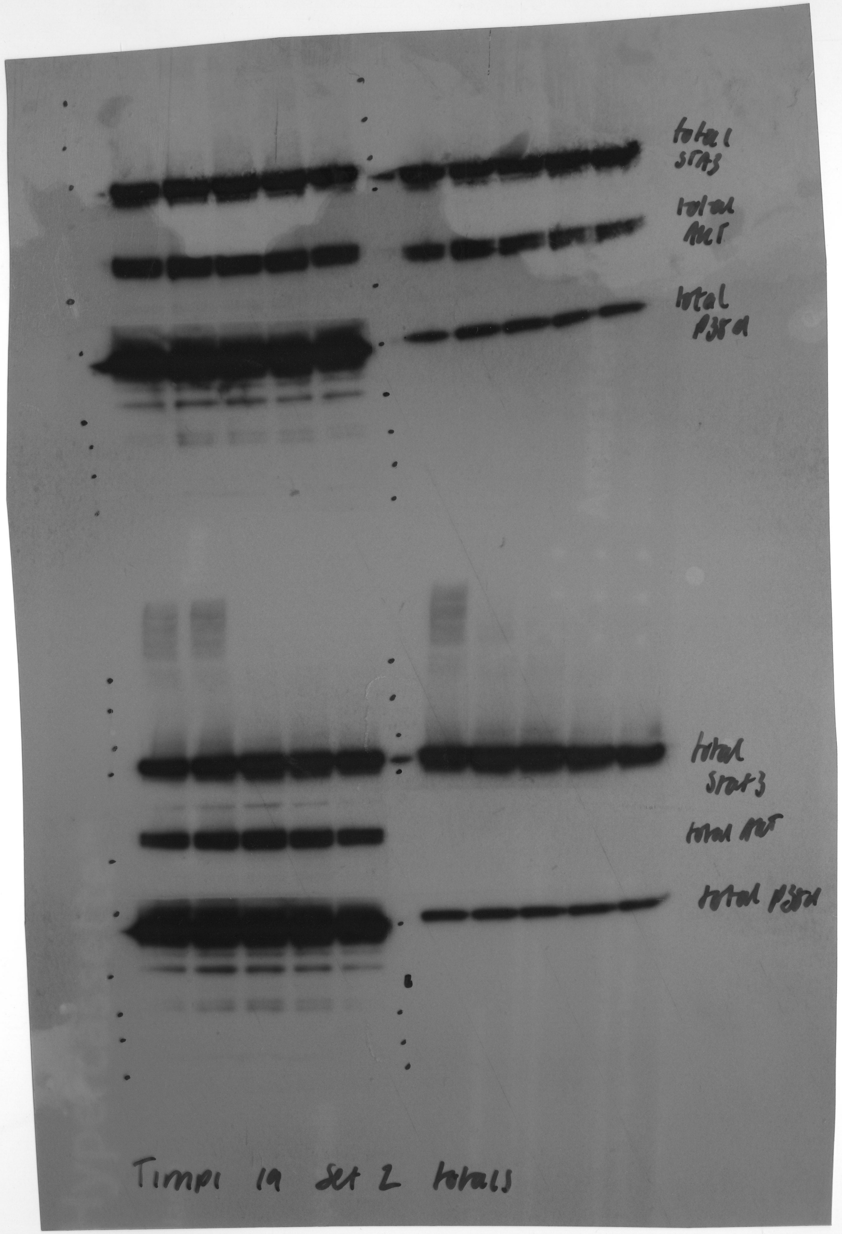
total STAT3
total AKT
total p38α
total STAT3
total AKT
total p38α
Timp1 19 set 2 totals

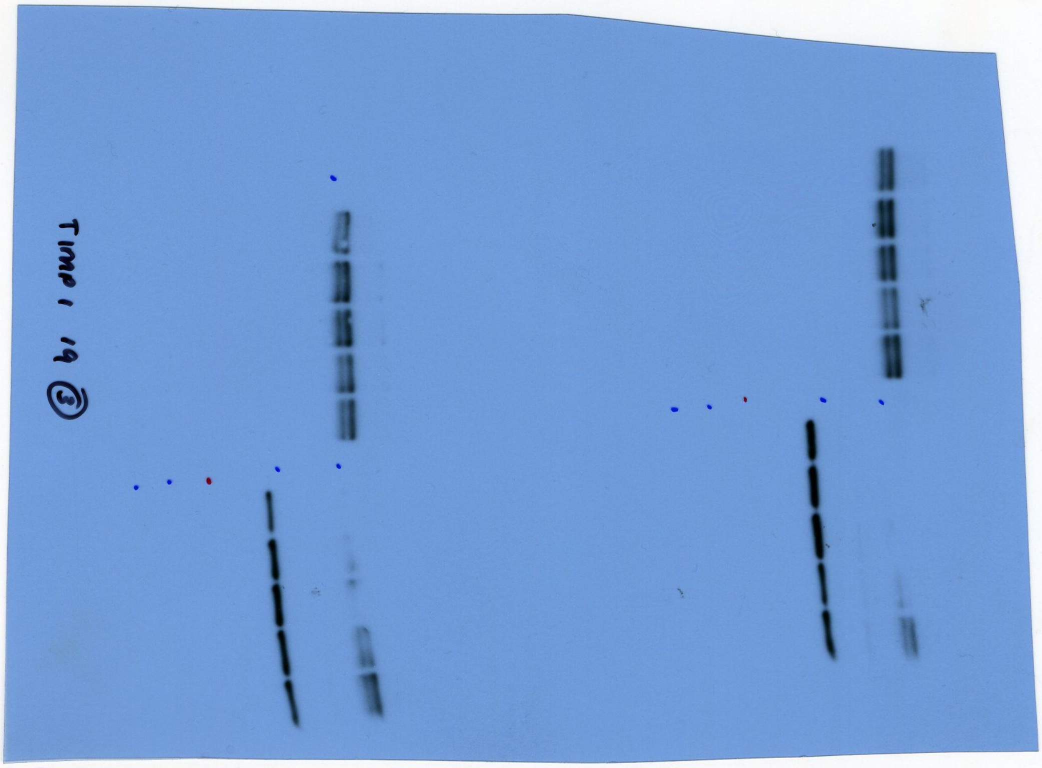
Timp1 19 (3)

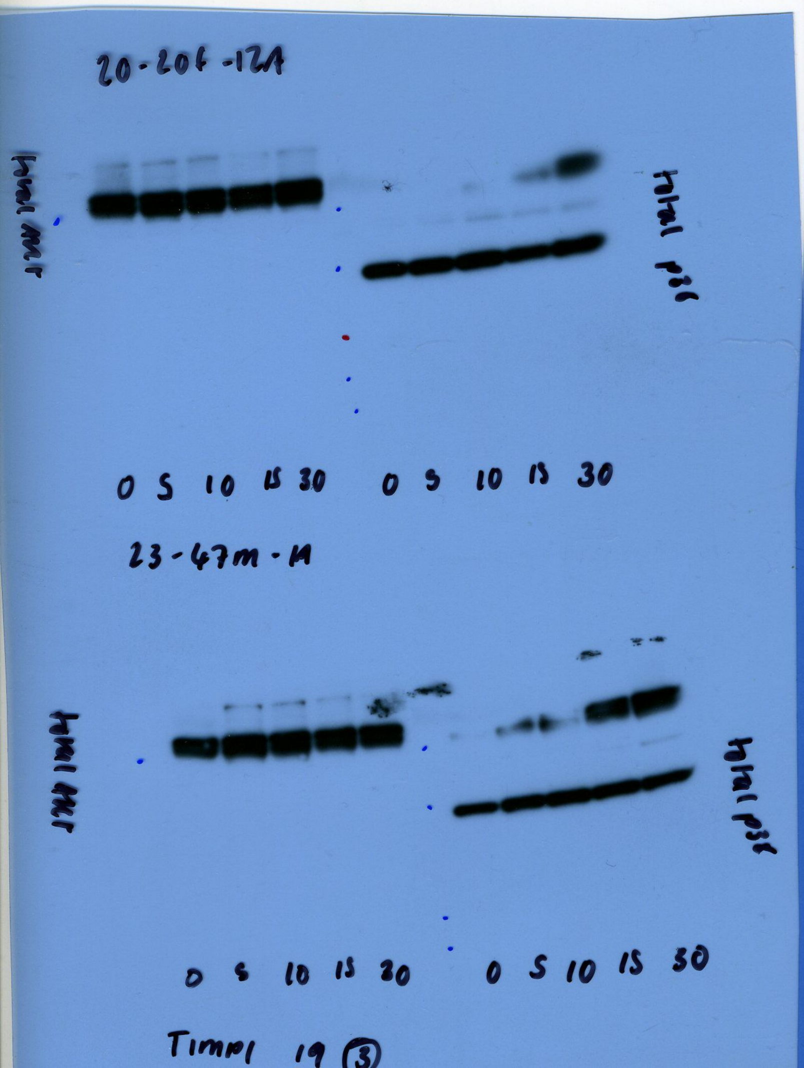
20-206-12A
total AKT
total p38
0 5 10 15 30 0 5 10 15 30
23-47m-1A
total AKT
total p38
0 5 10 15 20 0 5 10 15 30
Timp1 19 (3)

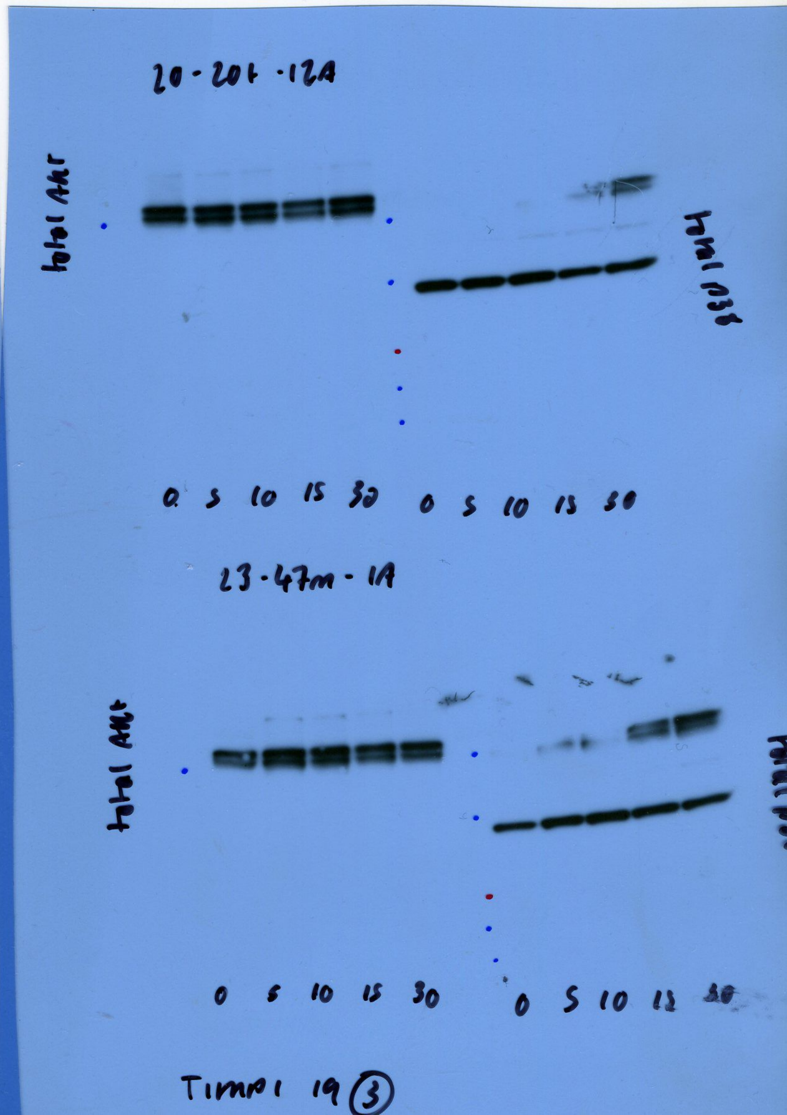
20-206-12A
total AKT
total p38
0 5 10 15 30 0 5 10 15 30
23-47m-1A
total AKT
0 5 10 15 30 0 5 10 15 30
Timp1 19 (3)

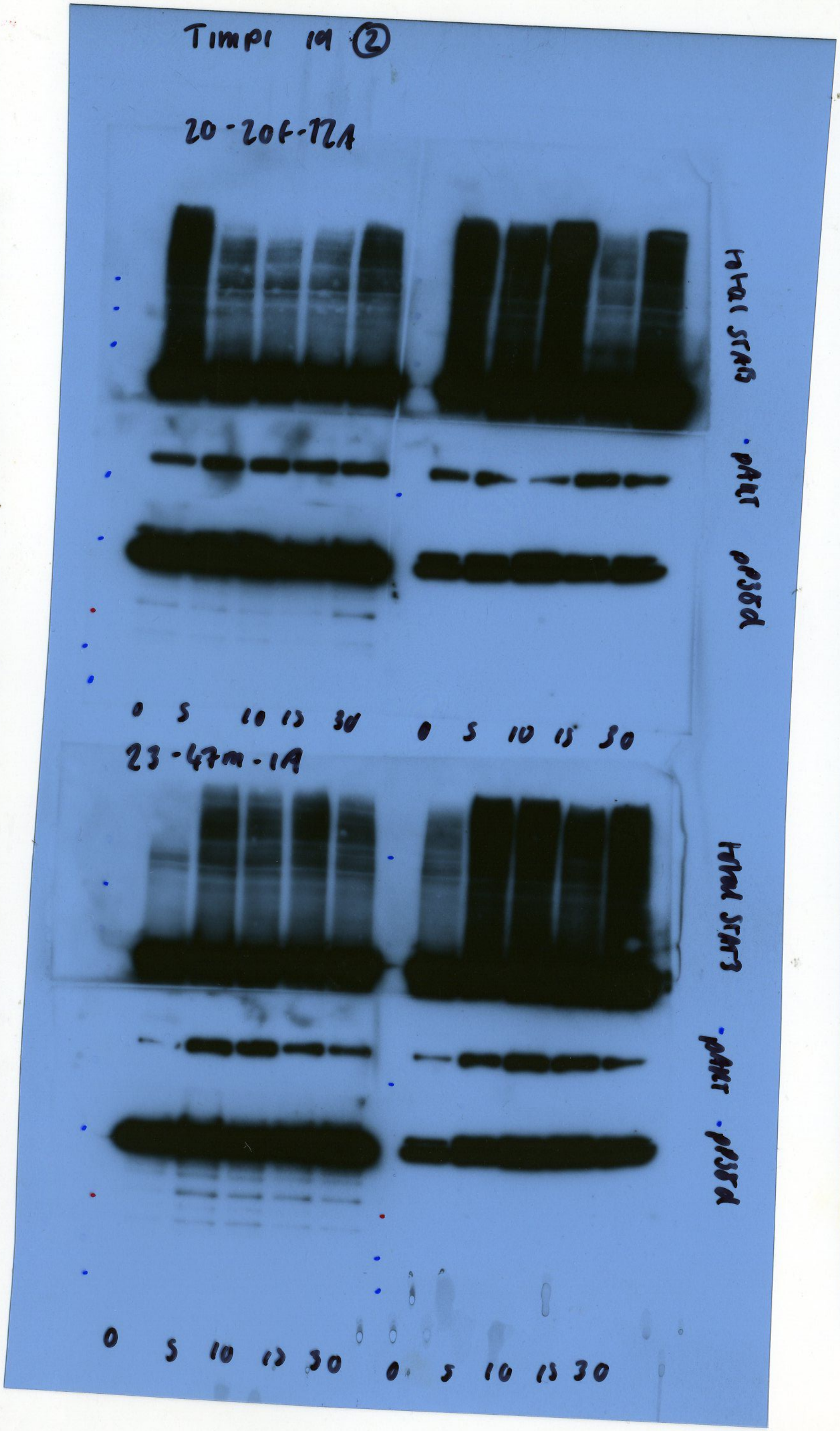
Timpi 1a ②
20-20f-12A
total STAT3
pAKT
pP38α
0 5 10 15 30 0 5 10 15 30
23-47m-1A
total STAT3
pAKT
pP38α
0 5 10 15 30 0 5 10 15 30

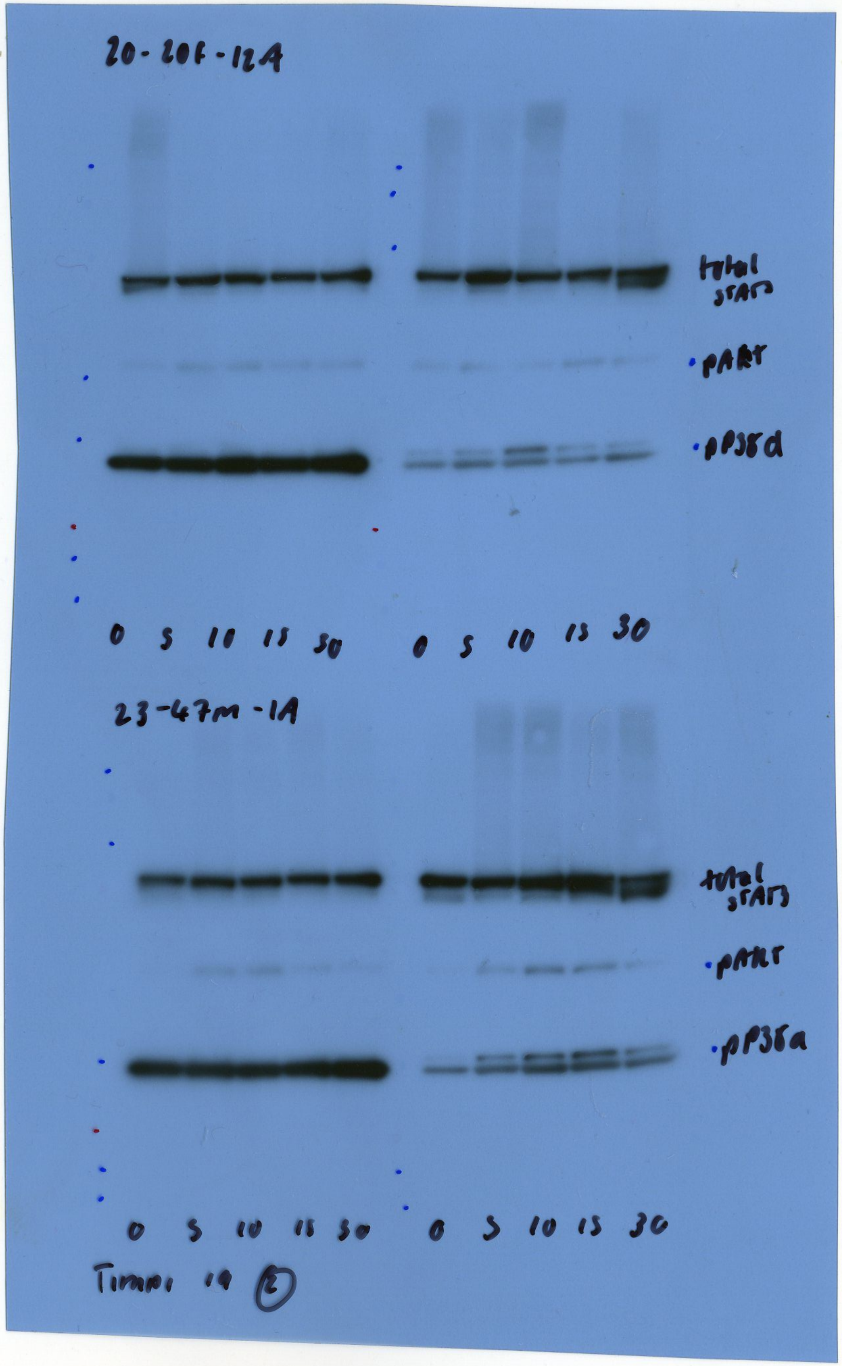
20-20f-12A
total STAT3
pAKT
pP38α
0 5 10 15 30 0 5 10 15 30
23-47m-1A
total STAT3
pAKT
pP38α
0 5 10 15 30 0 5 10 15 30
Timpi 1a

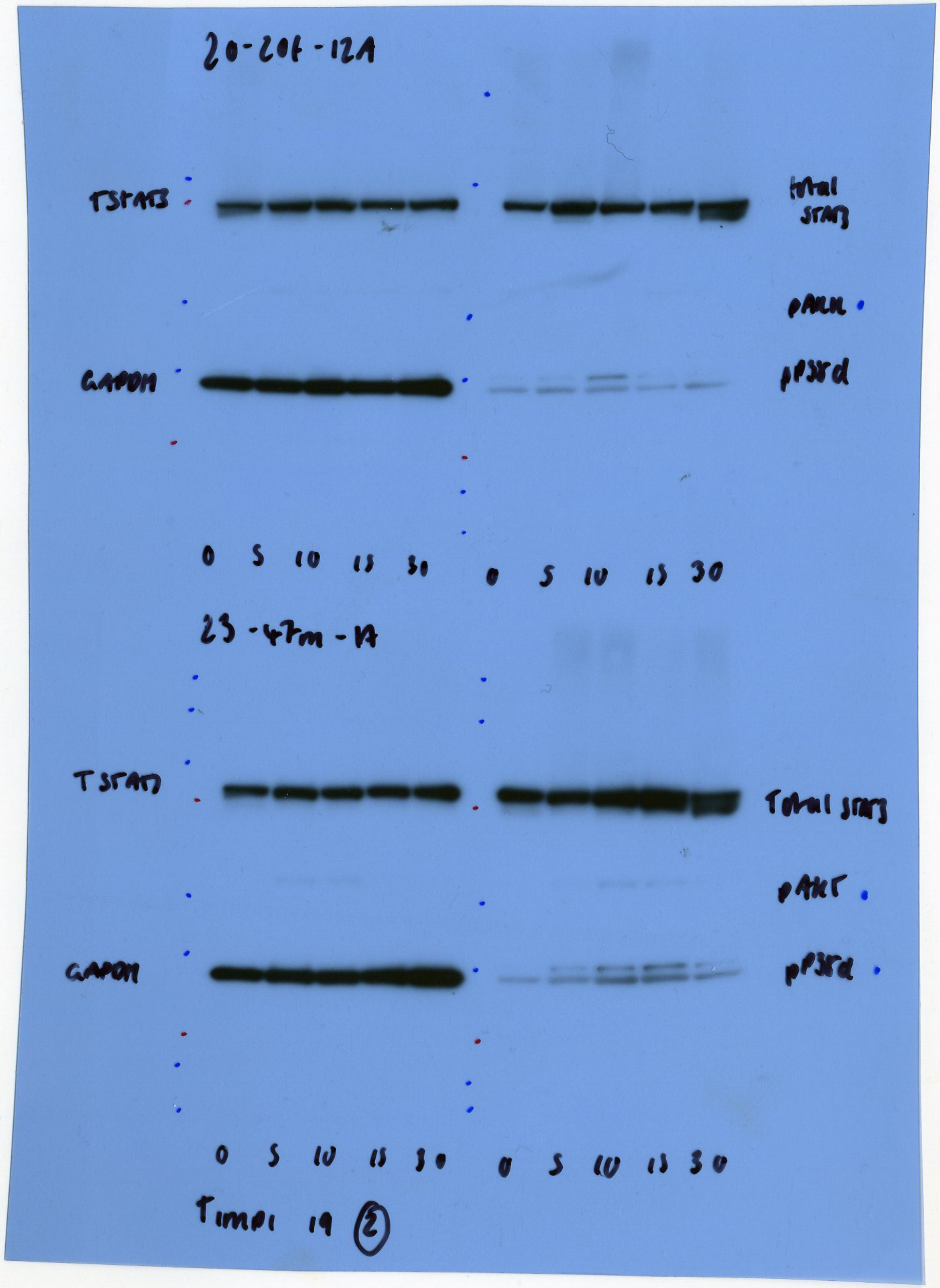
20-20f-12A
TSTAT3
total STAT3
pAKt
GAPDH
pP38α
0 5 10 15 30
0 5 10 15 30
23-47m-1A
TSTAT3
Total STAT3
pAKT
GAPDH
pP38α
0 5 10 15 30
0 5 10 15 30

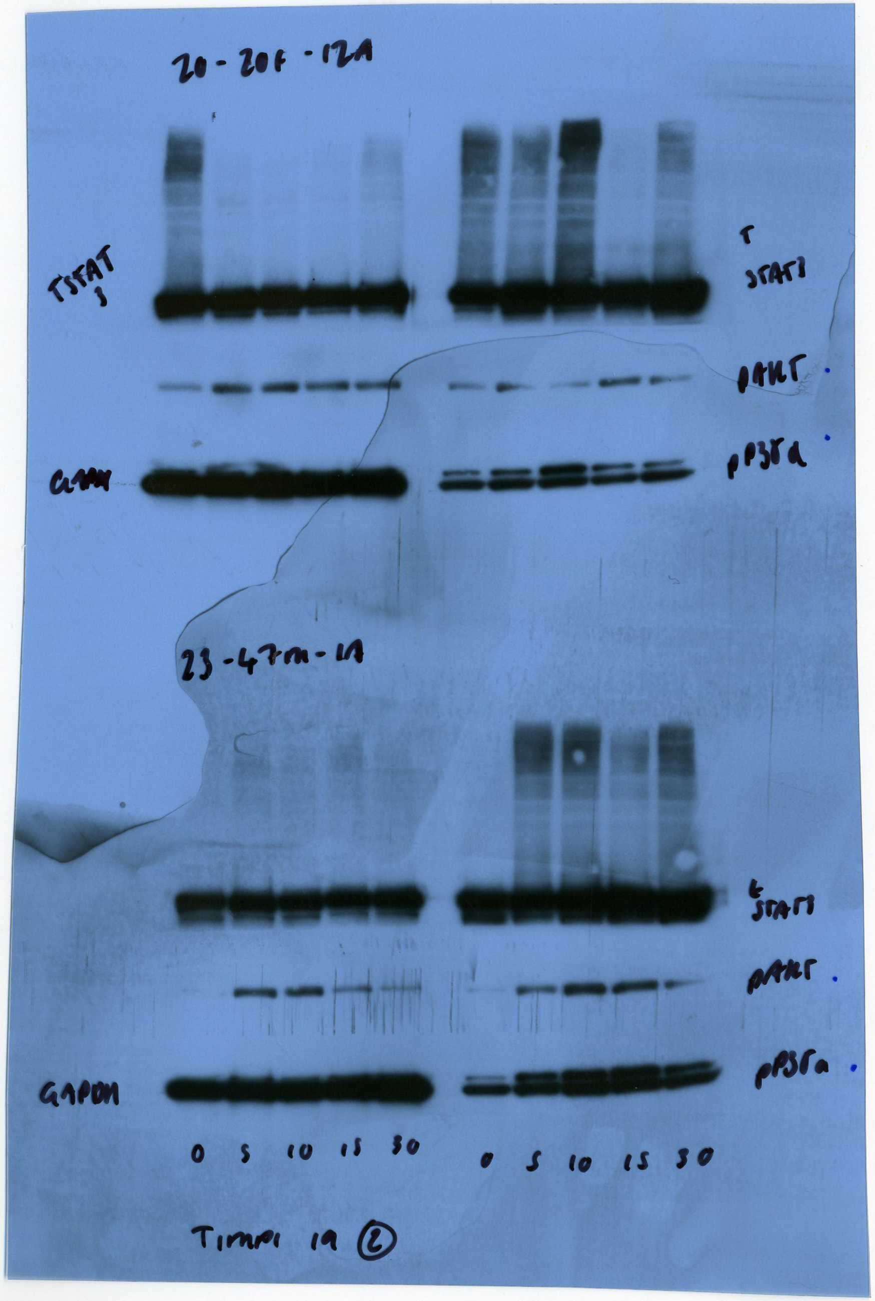
20-20f-12A
TSTAT3
T STAT3
pAKT
GAPDH
pP38α
23-47m-1A
t STAT3
pAKT
GAPDH
pP38α
0 5 10 15 30
0 5 10 15 30

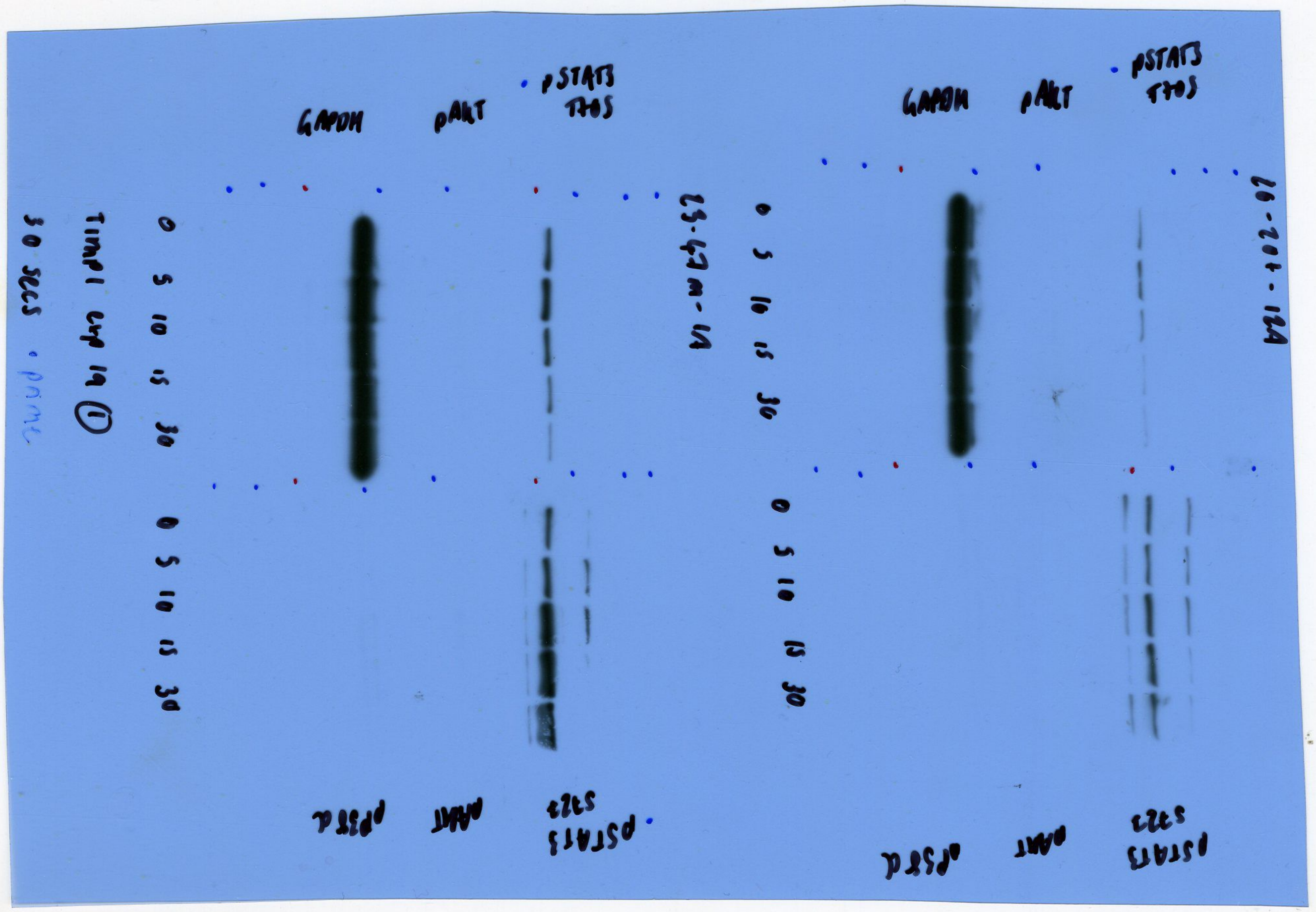
GAPDH
pAKT
pSTAT3 T705
23-47m-1A
20-207-12A
0 5 10 15 30
Timp1 exp 19
30 secs
pSTAT3 S727

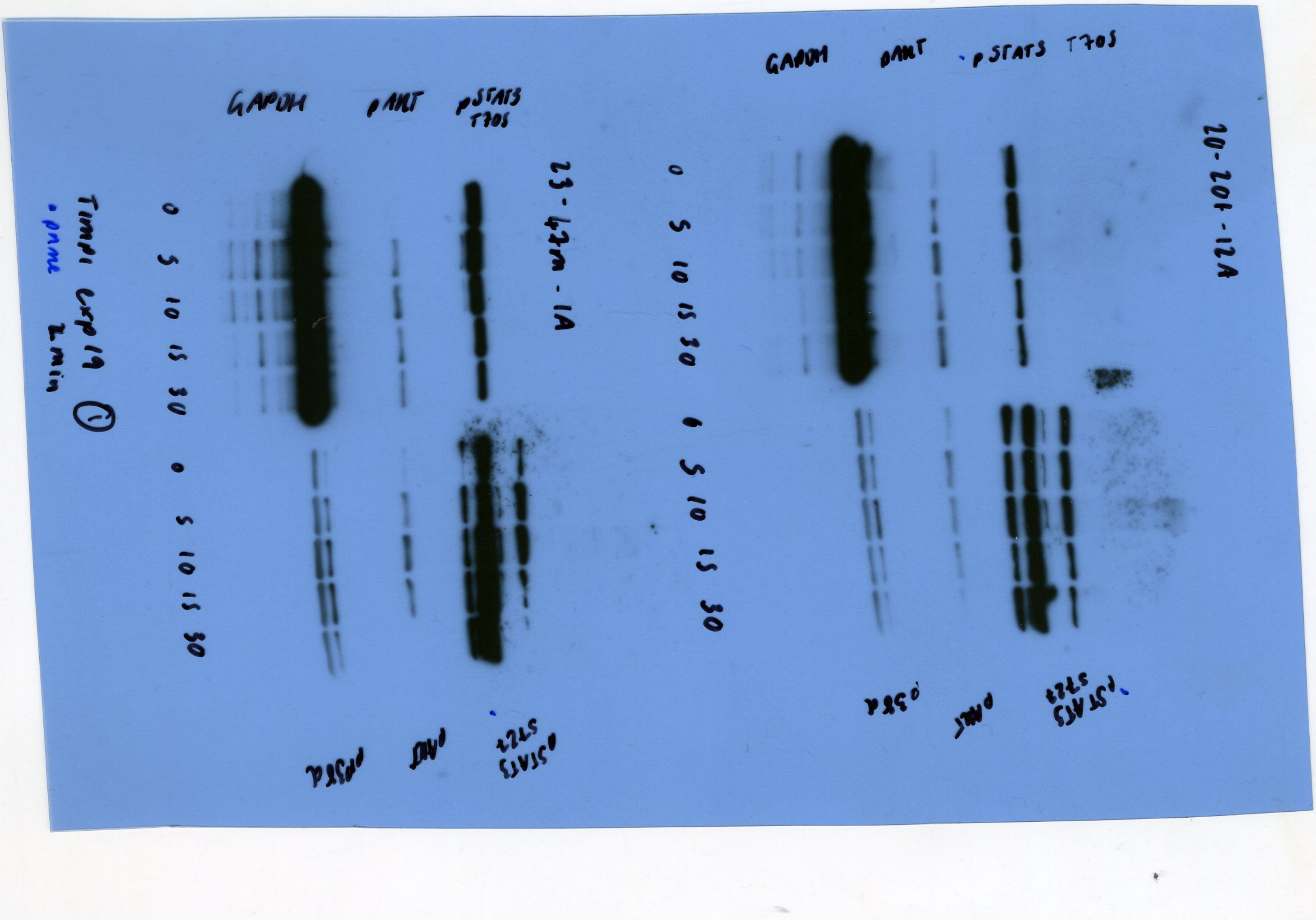
GAPDH
pAKT
pSTAT3 T705
23-47m-1A
20-207-12A
0 5 10 15 30
Timp1 exp 19
2 min
pSTAT3 S727

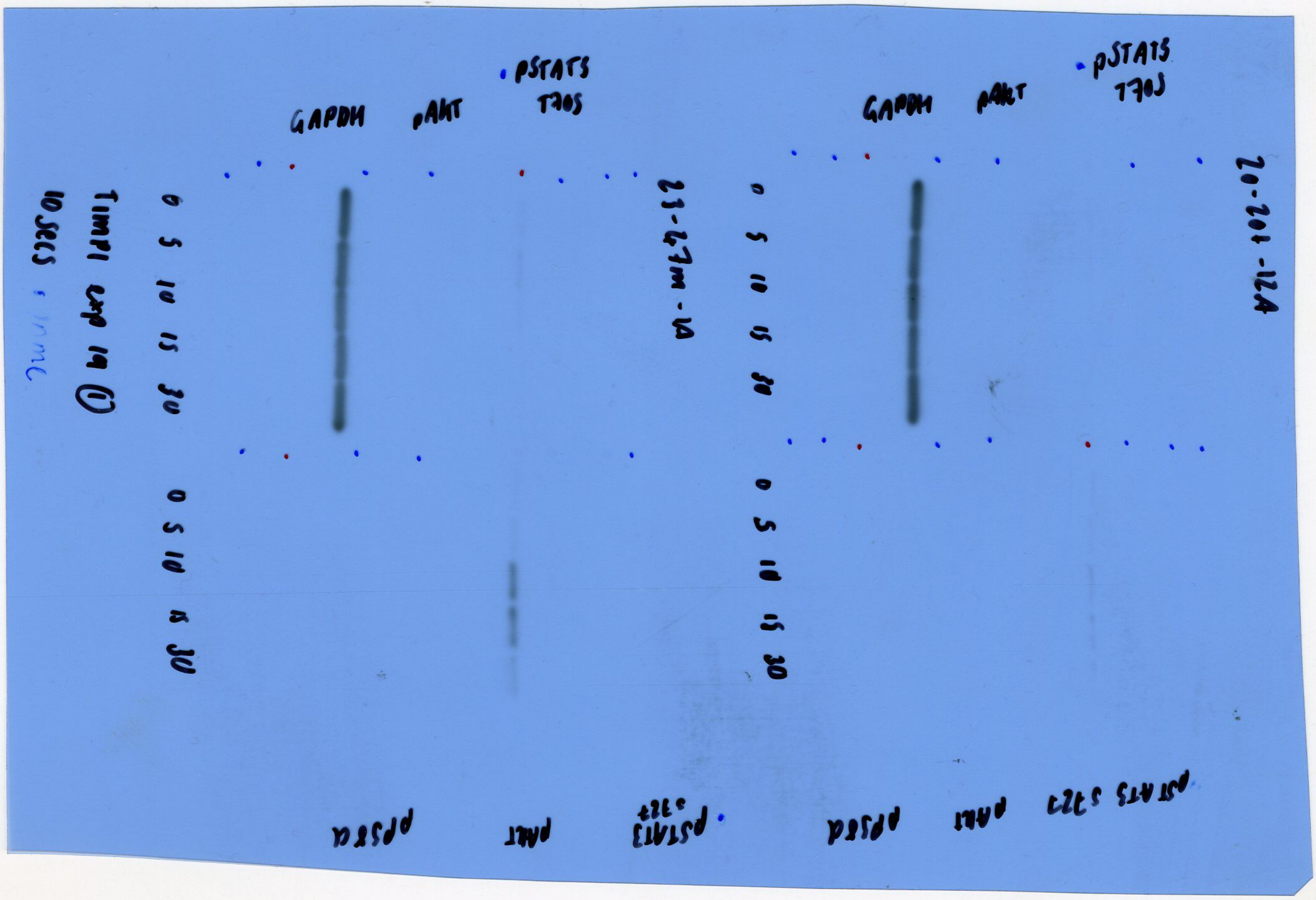
GAPDH
pAKT
pSTAT3 T705
20-207-12A
23-47m-1A
Timp1 exp 1a (1)
10 secs
0 5 10 15 30
pSTAT3 S727
pAKT
p38α

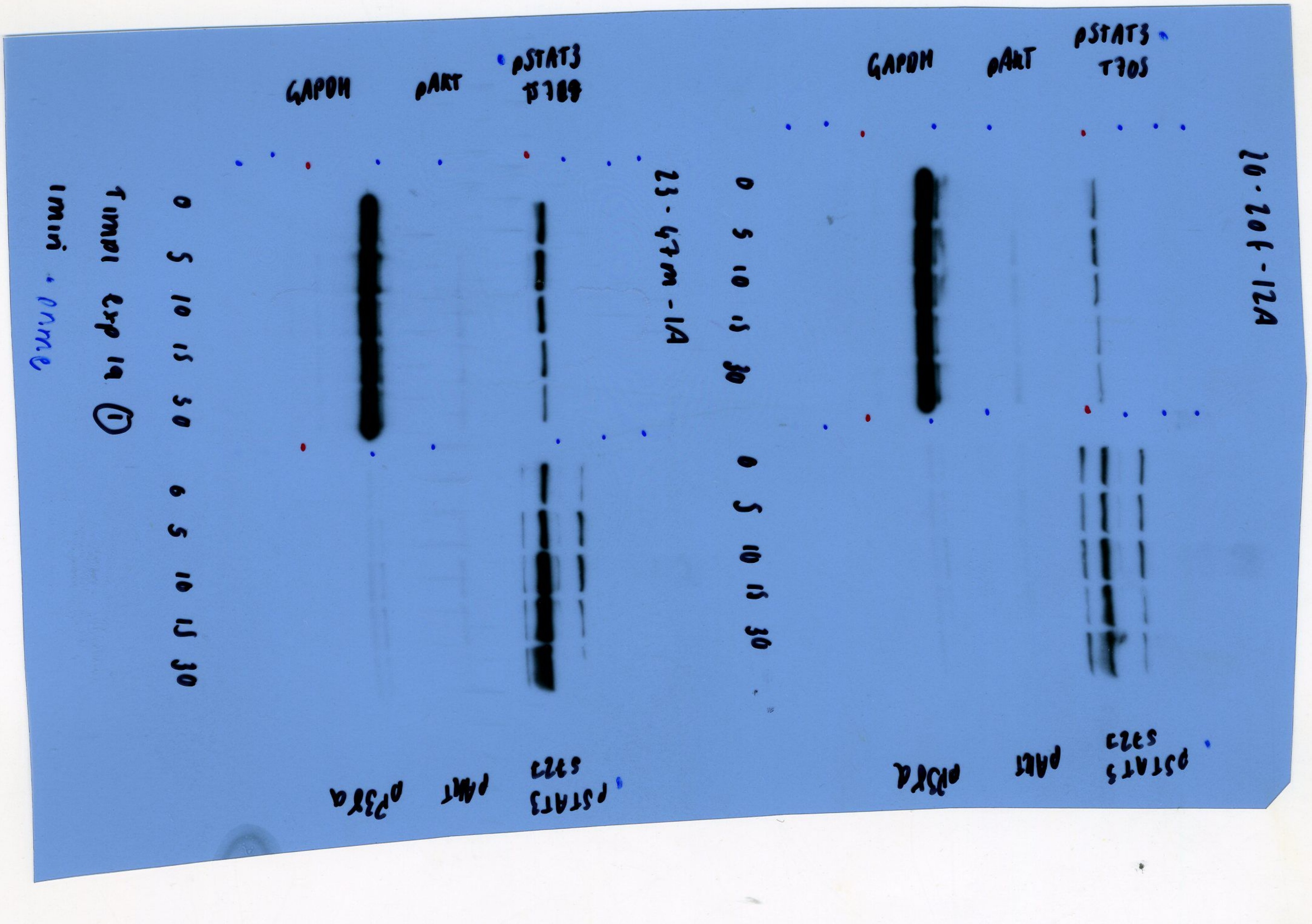
GAPDH
pAKT
pSTAT3 T705
20-207-12A
23-47m-1A
Timp1 exp 1a (1)
1min
0 5 10 15 30
pSTAT3 S727
pAKT
p38α

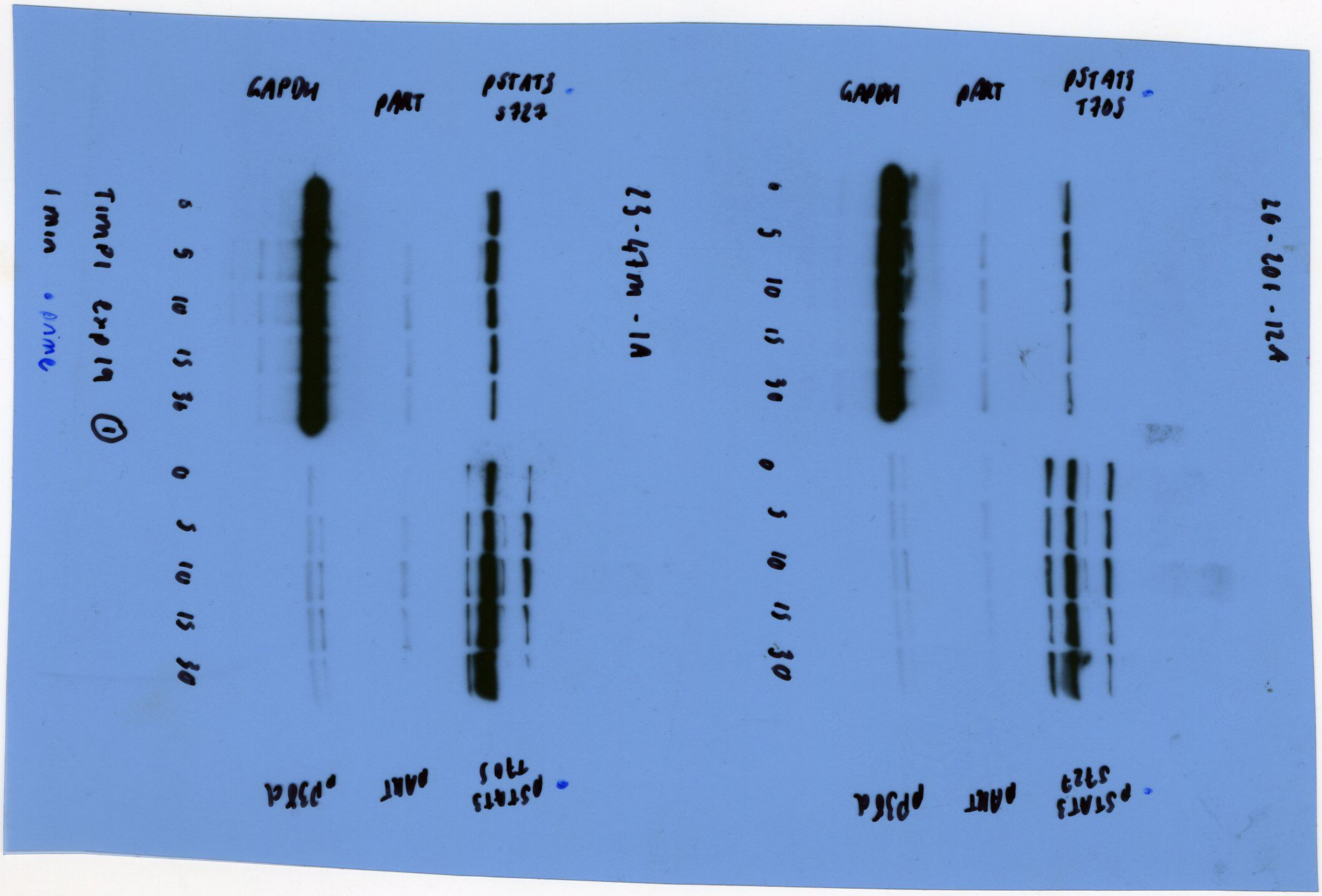
GAPDH
pAKT
pSTAT3 S727
pSTAT3 T705
0 5 10 15 30
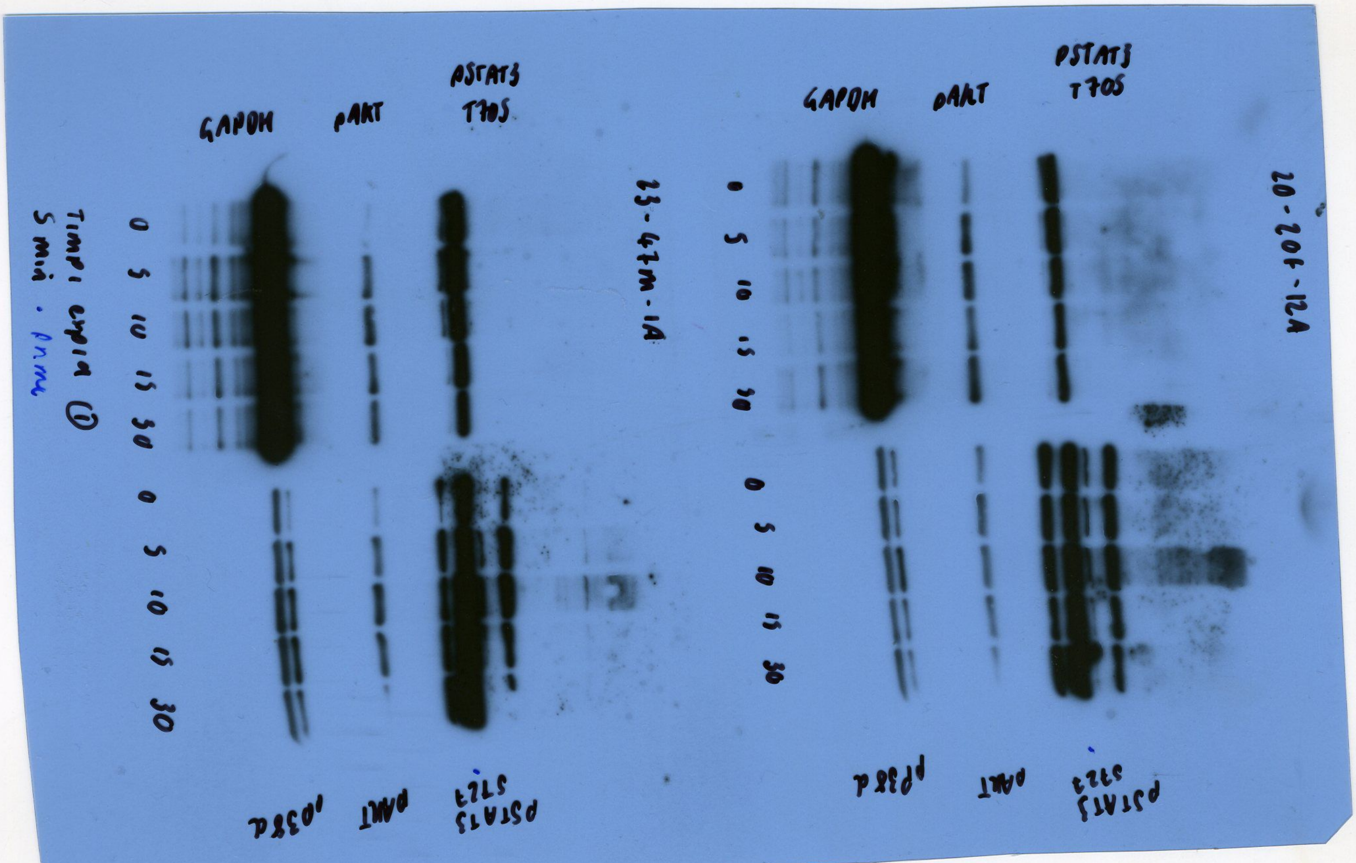
GAPDH
pAKT
pSTAT3 T705
pSTAT3 S727
0 5 10 15 30
