# Supplementary material for: Network-based prioritization and validation of regulators of vascular smooth muscle cell proliferation in disease
Source: Nat Cardiovasc Res. 2024 Jun 6;3(6):714–33. doi: 10.1038/s44161-024-00474-4 (PMC11182749; doi:10.1038/s44161-024-00474-4)
Supplement: Supplementary file 7 [file 44161_2024_474_MOESM12_ESM.zip › Lambert_Oc_SourceData_fig8/Lambert_Oc_unmodified_westerns_fig8.pdf]

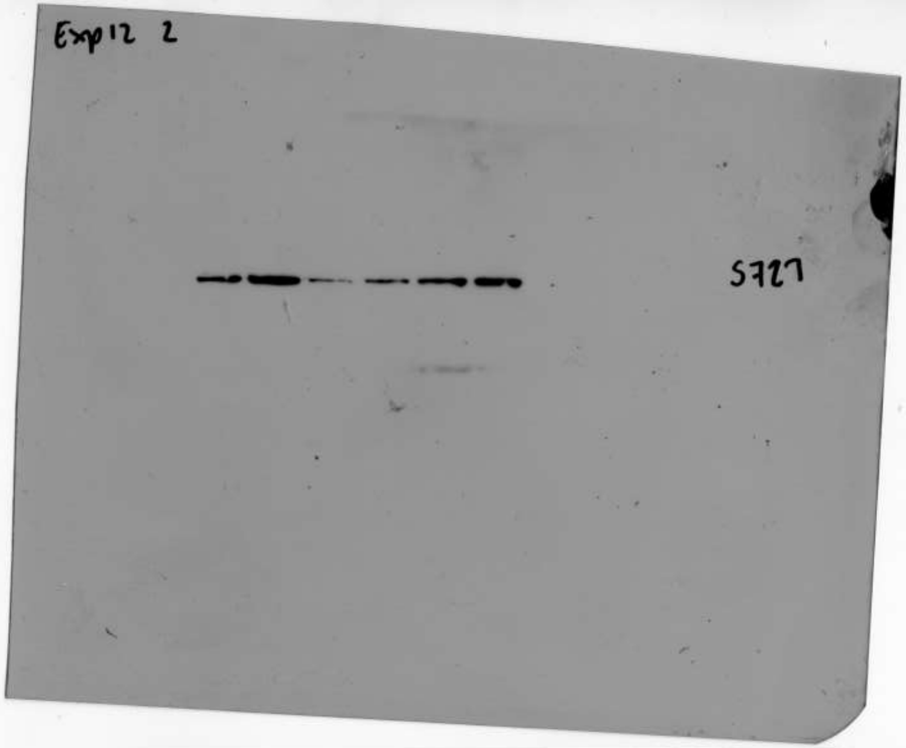
Exp12 2
S727

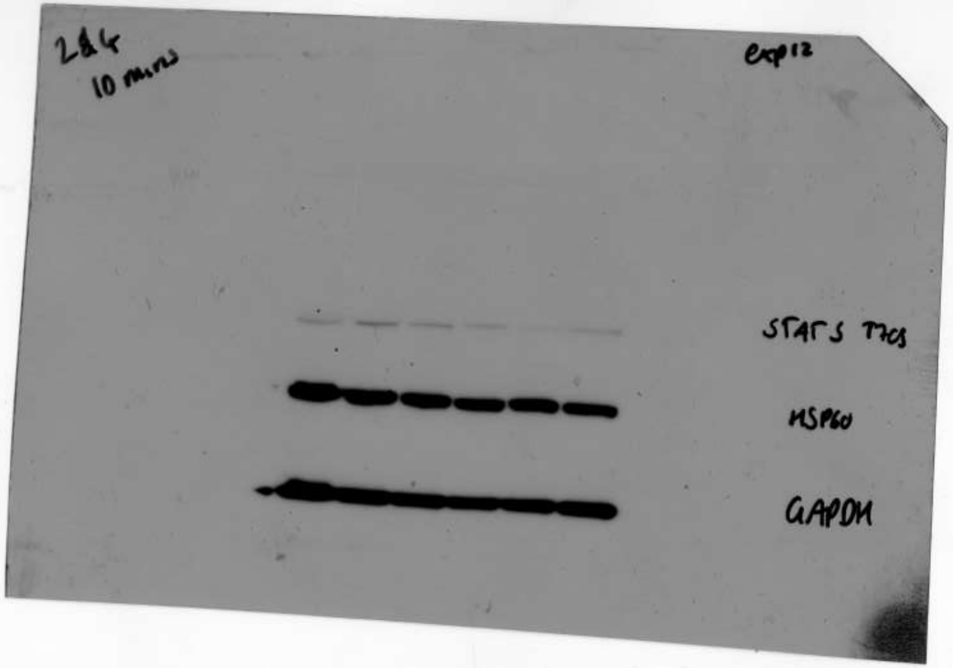
10 mins
Exp12
STAT5 T705
HSP60
GAPDH

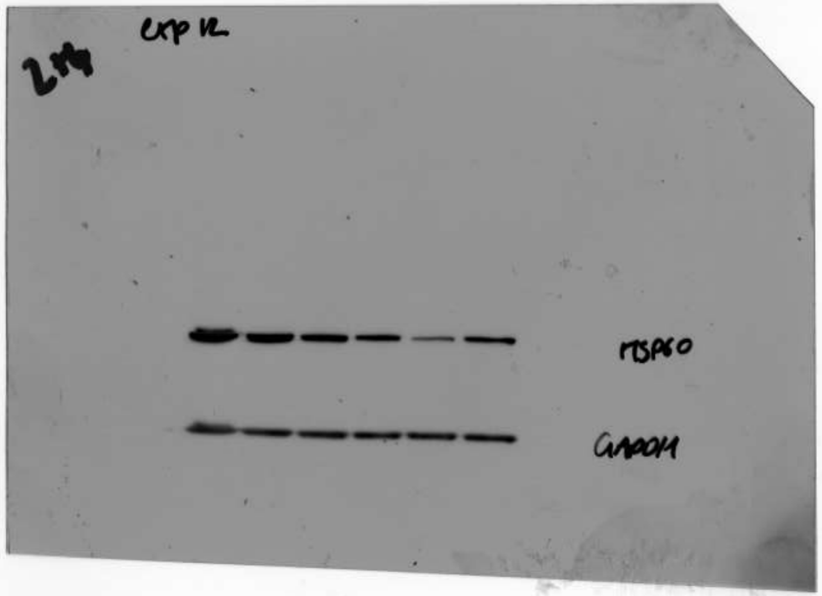
Exp 12
HSP60
GAPDH

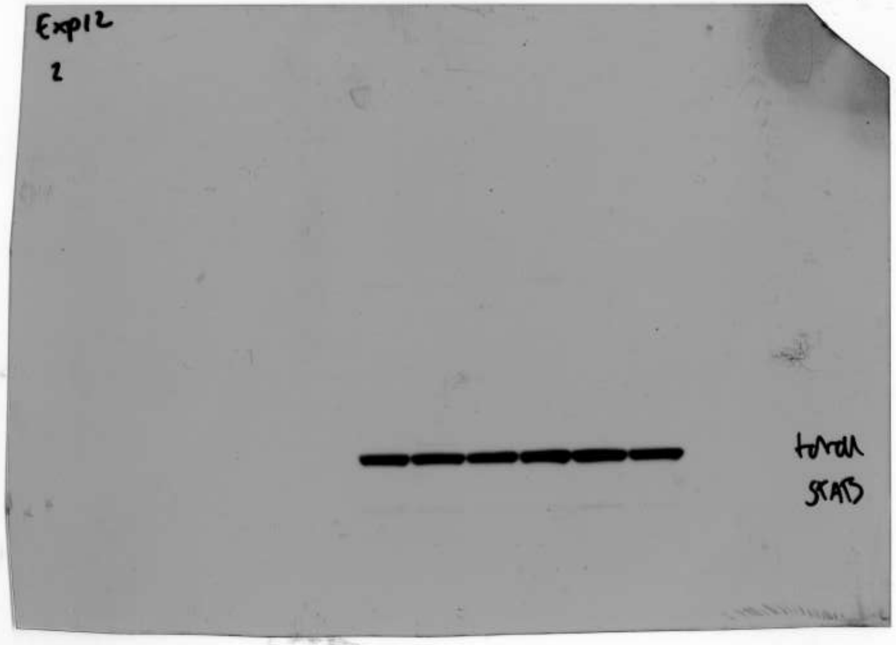
Exp12
2
total
STAT3

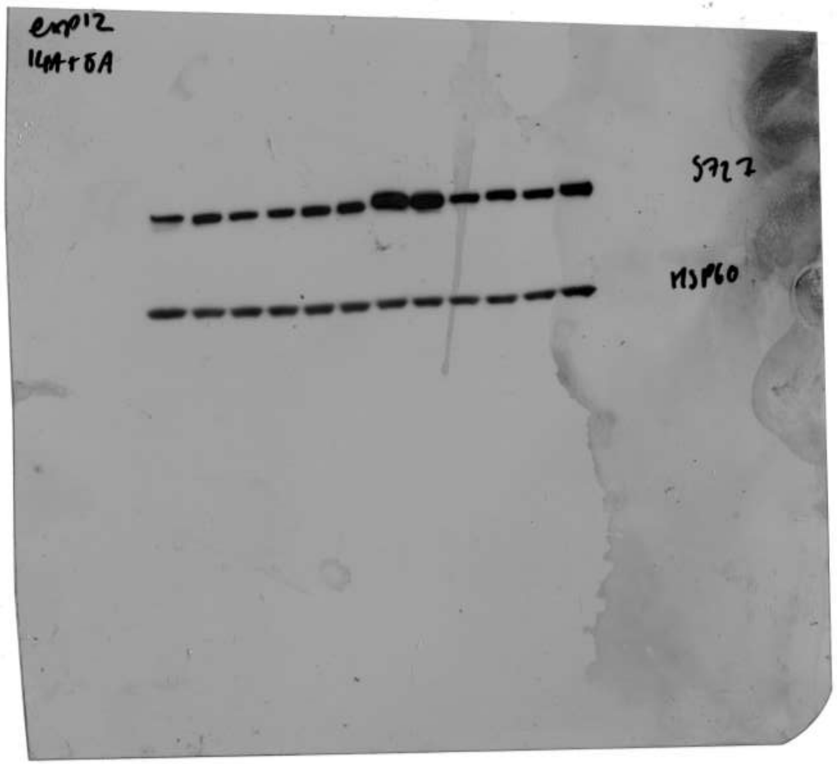
exp12
14A+8A
S722
HSP60

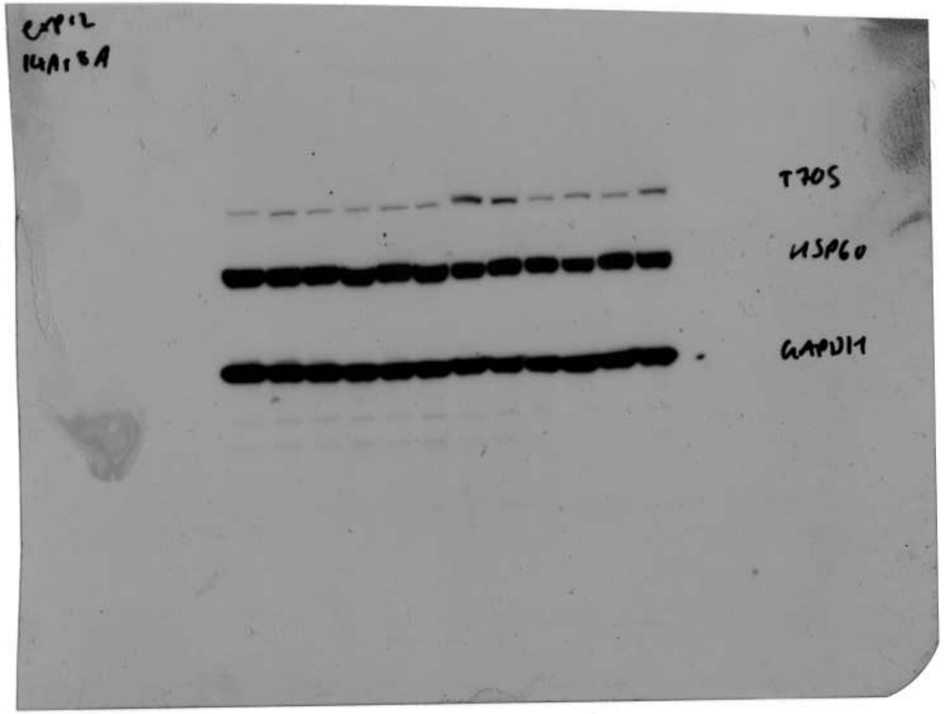
exp12
14A+8A
T70S
HSP60
GAPDH

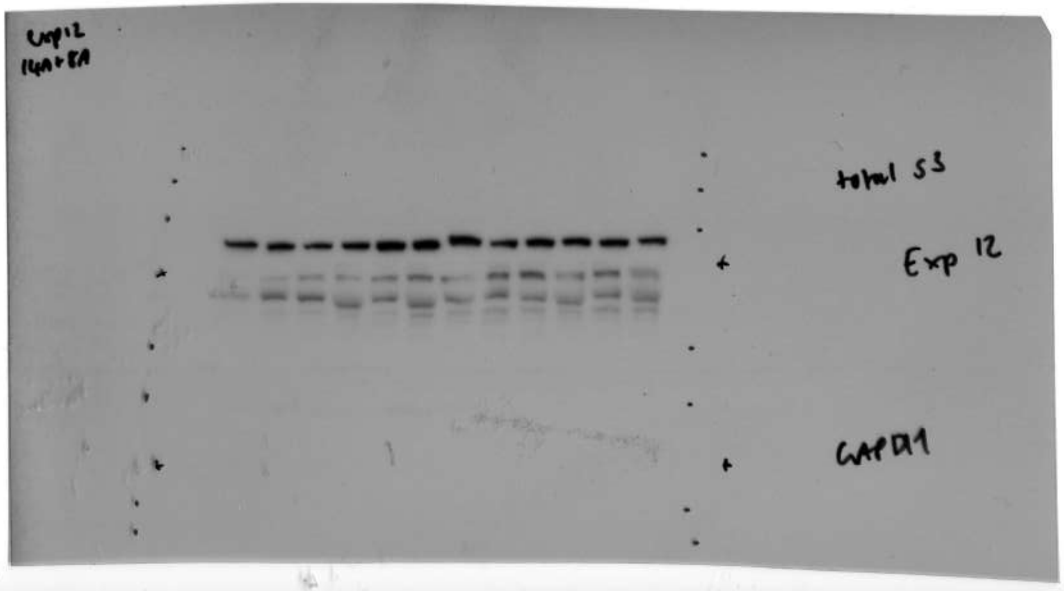
exp12
14A+8A
total S3
Exp 12
GAPDH

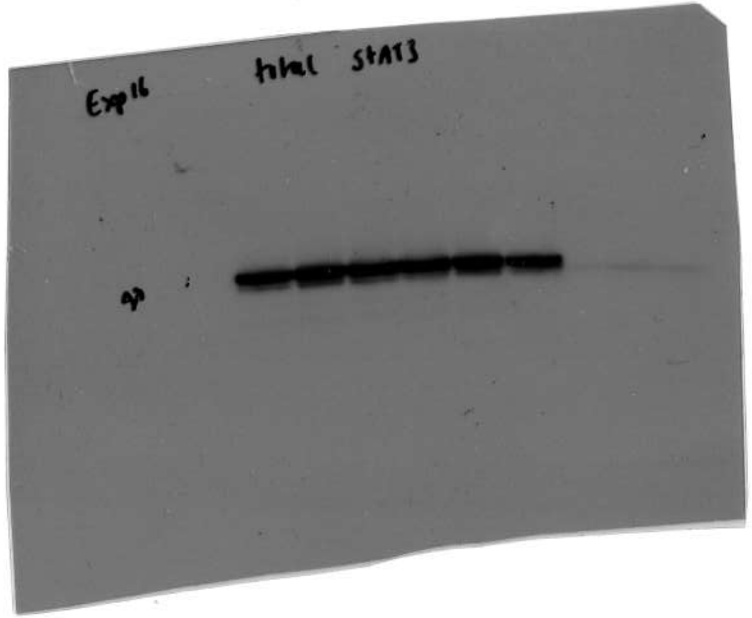
Exp16
total STAT3

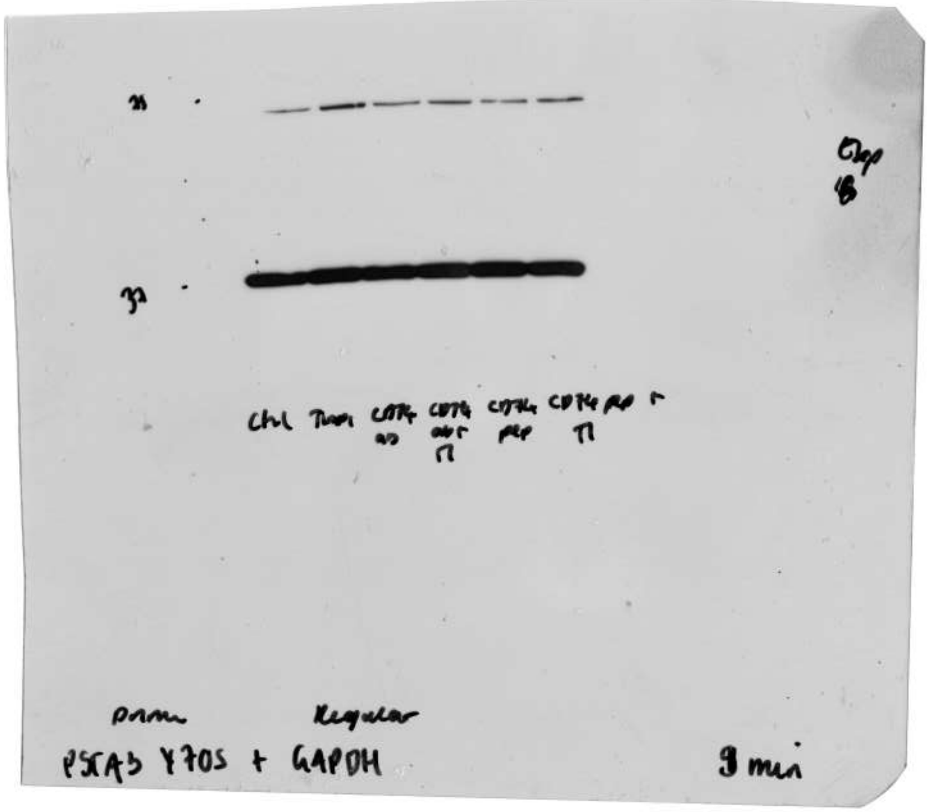
Ctrl
Regular
PSTA3 Y705 + GAPDH
9 min

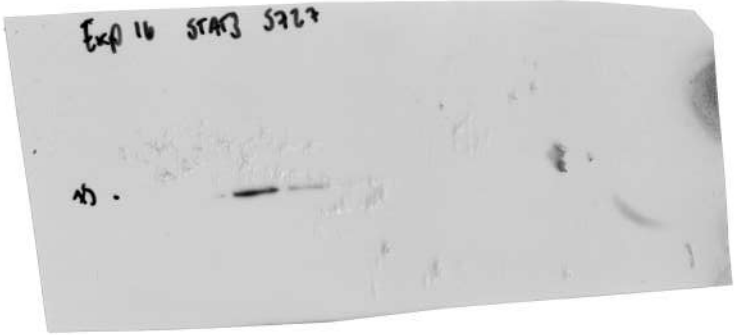
Exp 16 STAT3 S727
